# Spread tools: a systematic review of components, uptake, and effectiveness of quality improvement toolkits

**DOI:** 10.1186/s13012-019-0929-8

**Published:** 2019-08-19

**Authors:** Susanne Hempel, Claire O’Hanlon, Yee Wei Lim, Margie Danz, Jody Larkin, Lisa Rubenstein

**Affiliations:** 1 Southern California Evidence-based Practice Center, RAND Corporation, Santa Monica, USA; 2 Southern California Evidence Review Center, University of Southern California, Los Angeles, USA; 3 RAND Health, RAND Corporation, Los Angeles, USA; 4 Department of Medicine, Yong Loo Lin School of Medicine, National University of Singapore, Singapore, Singapore; 5 Knowledge Services, RAND Corporation, Santa Monica, USA

**Keywords:** Spread, Diffusion of innovation, Quality improvement, Toolkit, Implementation

## Abstract

**Background:**

The objective was to conduct a systematic review of toolkit evaluations intended to spread interventions to improve healthcare quality. We aimed to determine the components, uptake, and effectiveness of publicly available toolkits.

**Methods:**

We searched PubMed, CINAHL, and the Web of Science from 2005 to May 2018 for evaluations of publicly available toolkits, used a forward search of known toolkits, screened references, and contacted topic experts. Two independent reviewers screened publications for inclusion. One reviewer abstracted data and appraised the studies, checked by a second reviewer; reviewers resolved disagreements through discussion. Findings, summarized in comprehensive evidence tables and narrative synthesis addressed the uptake and utility, procedural and organizational outcomes, provider outcomes, and patient outcomes.

**Results:**

In total, 77 studies evaluating 72 toolkits met inclusion criteria. Toolkits addressed a variety of quality improvement approaches and focused on clinical topics such as weight management, fall prevention, vaccination, hospital-acquired infections, pain management, and patient safety. Most toolkits included introductory and implementation material (e.g., research summaries) and healthcare provider tools (e.g., care plans), and two-thirds included material for patients (e.g., information leaflets). Pre-post studies were most common (55%); 10% were single hospital evaluations and the number of participating staff ranged from 17 to 704. Uptake data were limited and toolkit uptake was highly variable. Studies generally indicated high satisfaction with toolkits, but the perceived usefulness of individual tools varied. Across studies, 57% reported on adherence to clinical procedures and toolkit effects were positive. Provider data were reported in 40% of studies but were primarily self-reported changes. Only 29% reported patient data and, overall, results from robust study designs are missing from the evidence base.

**Conclusions:**

The review documents publicly available toolkits and their components. Available uptake data are limited but indicate variability. High satisfaction with toolkits can be achieved but the usefulness of individual tools may vary. The existing evidence base on the effectiveness of toolkits remains limited. While emerging evidence indicates positive effects on clinical processes, more research on toolkit value and what affects it is needed, including linking toolkits to objective provider behavior measures and patient outcomes.

**Trial Registration:**

PROSPERO registration number: PROSPERO 2014:CRD42014013930.

**Electronic supplementary material:**

The online version of this article (10.1186/s13012-019-0929-8) contains supplementary material, which is available to authorized users.

## Background

Diffusion of innovations is a complex process. While research studies continue to show successful interventions to improve healthcare, their dissemination is slow [1–3]. Implementations of proof of concept studies and adoption of interventions shown to be effective in research studies into routine clinical practice is delayed or not achieved at all.

In recent years, a number of organizations have developed “toolkits” for healthcare quality improvement [4]. Toolkits are resource and tool collections designed to facilitate spread across settings and organizations and to ease the uptake and implementation of interventions or intervention bundles and practices. They are a resource for documentation of interventions, for implementation of successful interventions, and for scaling up initiatives developed in pilot or demonstration sites into large-scale rollouts. Toolkits may include a variety of materials useful to organizations to help introduce an intervention, practical tools to help incorporate best practices into routine care such as pocket cards for healthcare providers, or patient education materials. There is currently no definition of nor standard approach to toolkit contents or formats.

A variety of healthcare research agencies publish toolkits. The US Agency for Healthcare Research and Quality (AHRQ) alone has published a large number, on topics ranging from allergy and immunologic care to urologic care. The AHRQ Healthcare Innovations Exchange website has tracked the development of tools or toolkits to improve quality and reduce disparities (website maintenance ended in 2017). Users may browse the resources online or download them free of charge. Little is known, however, about uptake of published toolkits. While exact copying of the intervention is possible, a process of re-invention in the new context is also likely to occur. Re-invention may change the intervention to some extent during the diffusion process as it transitions from the developer to the adopter, with or without the help of a toolkit [5], potentially resulting in decreased but still significant effort for toolkit adaptation [6]. To date, we know very little about successful components that may be useful across toolkits, about the toolkit adoption process, or about what makes toolkits easier or harder to adopt.

Furthermore, little is known about the effectiveness of published toolkits. A scoping review describing toolkits assembled for individual research projects concluded that the toolkits often did not specify the evidence base from which they draw and their effectiveness as a knowledge translation strategy was rarely assessed [1, 7]. The effectiveness of a toolkit is likely to depend on its quality, the effectiveness of the intervention, and the setting characteristics. However, for published toolkits, an additional consideration is apparent. Toolkits applied in new settings may not be as effective as seen in the original implementation of the intervention bundle that led to the development of the toolkit. Potential reasons include diminished healthcare provider motivation, reduced staff buy-in, or other aspects of low readiness (e.g., healthcare providers were not instrumental in initiating and shaping the interventions).

Our objective was to conduct a systematic review on the spread of interventions intended to improve healthcare quality through toolkits. This systematic review aims to determine the following key questions:

- Key question 1: What are the components of published quality improvement toolkits?
- Key question 2: What is the uptake and utility of published quality improvement toolkits?
- Key question 3: What is the effectiveness of published quality improvement toolkits?

The review explores the types of tools included in toolkits, measures and results that describe the uptake and utility, and the effectiveness of published toolkits to inform users and developers of toolkits.

## Methods

We registered in PROSPERO, registration number PROSPERO 2014:CRD42014013930. The reporting follows the PRISMA guidelines (see Additional file 1).

### Searches

We searched the databases PubMed, CINAHL, and Web of Science for evaluations of toolkits in May 2018. The PubMed search strategy is given in full in Additional file 2. The strategy searched for the term “toolkit” in the title, abstract, keywords, or full text of the publication (Web of Science only). We did not limit the search to publications using the MeSH term “diffusion of innovation” because the pilot search strategy showed that known toolkit evaluations were not systematically tagged with this term. We limited to English-language citations published since 2005 to identify current toolkits readily applicable to US settings.

In addition, we searched resources from nine organizations dedicated to healthcare improvement to find published toolkits: AHRQ, World Health Organization (WHO), Institute for Healthcare Improvement [IHI], Robert Wood Johnson Foundation [RWJF], Association of perioperative Registered Nurses [AORN], Emergency Care Research Institute [ECRI], Centers for Disease Control and Prevention (CDC), Centers for Medicare and Medicaid Services (CMS), and Department of Veterans Affairs (VA). We also screened the category “QualityTool” in AHRQ’s database of innovations. A “forward search” identified any publication that had cited the titles of the toolkits we located. We screened included studies and relevant reviews and contacted content experts to identify additional relevant publications.

### Study inclusion and exclusion criteria

Two independent reviewers screened titles and abstracts to avoid errors and bias. We obtained publications deemed as potentially relevant by at least one reviewer as full text. Full text publications had to meet the outlined criteria to be eligible for inclusion in the review. Discrepancies were resolved through discussion in the review team. In the absence of a universally agreed definition of a toolkit, the project team developed the outlined working definition.

- Participants and condition being studied: Publications evaluating toolkits in healthcare delivery organizations were eligible. The review was not limited to toolkits targeting specific clinical conditions, but toolkits had to be aimed at healthcare. Toolkits aimed primarily at other than healthcare provider professions (e.g., policy makers in non-healthcare delivery settings), or aimed at students not yet involved in healthcare delivery (e.g., nursing students) were excluded. Toolkits only aimed at patients, such as patient education material or patient self-management programs, were excluded.
- Intervention and toolkit definition: Studies evaluating the use of toolkits designed to aid healthcare delivery organizational were eligible. A “toolkit” was defined as an intervention package, or set of tools. Toolkits had to be aimed at quality improvement (an effort to change/improve the clinical structure, process, and/or outcomes of care by means of an organizational or structural change) [8] of healthcare; toolkits to increase research capacity or workforce issues were excluded. Test batteries, image processing protocols, or computer software termed “toolkit” were not eligible. Toolkits had to be either publicly or commercially available.
- Comparator/study design: Studies evaluating the use of existing toolkits were eligible. Studies supporting the development of toolkits and reporting on earlier versions rather than the currently available toolkits were excluded. Controlled and uncontrolled studies with historic (e.g., pre-post studies) or concurrent comparators (e.g., randomized controlled trials, RCTs) were eligible. Comparators could include active controls (a different intervention) or passive controls (e.g., status before the introduction of the toolkit).
- Outcome: Publications reporting on patient, provider, or organizational findings were eligible. Studies had to report on structured evaluations (e.g., surveys); informal or anecdotal evaluation statements were not sufficient.
- Timing: To capture current and relevant toolkits developed in accordance with current standards and applicable material, evaluated toolkits must have been published in 2005 or more recently, or be still available.
- Setting: Implementations of toolkits were included regardless of the setting, but the original toolkits had to be aimed at quality improvement in health care. Toolkits developed for other than healthcare delivery organizations such as school settings or laboratories as well as toolkits primarily focusing on health system improvements in conflict zones or disrupted healthcare systems were excluded.

**Fig. 1:**
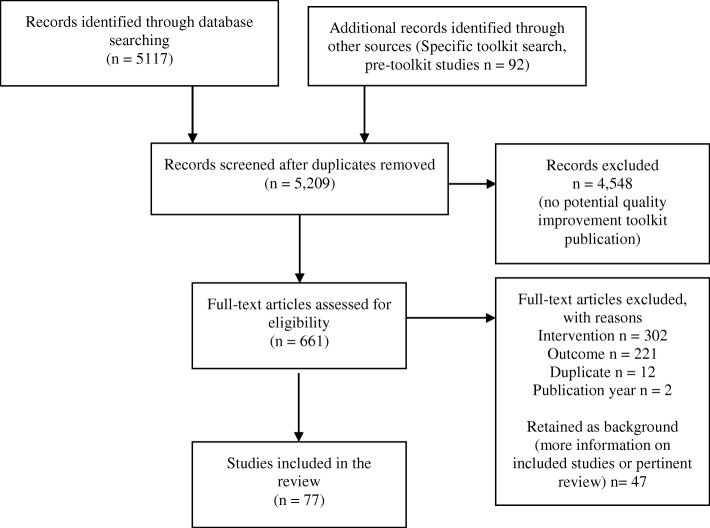
Literature flow diagram

We consolidated publications reporting on the same sample of participants. Evaluations published in academic journals as well as gray literature (conference abstracts, dissertations) were eligible. The literature flow diagram is shown in Fig. 1.

### Potential effect modifiers and reasons for heterogeneity

The review included a large number of study designs and study outcomes to allow a comprehensive overview of the available evidence on toolkits. In particular, the study design (e.g., comparative studies, post-only study) and the study outcomes (e.g., feasibility, patient health outcome) were sources of heterogeneity across studies.

### Data extraction strategy

One reviewer abstracted and a second experienced systematic reviewer checked the data; disagreements were resolved by discussion. We determined categories based on the initial review of publications and used a piloted-tested data extraction form to ensure standardized data abstraction.

We extracted the toolkit name, the developing organization, the general area of application, the toolkit components, and type of availability (publicly or commercially). In addition, information on the evaluation—including study design, participants, setting, and additional non-toolkit components—were extracted.

We documented the uptake and adherence to toolkit components (e.g., number of downloaded toolkits); utility and feasibility; healthcare provider measures including knowledge, attitudes, and barriers; procedural, structural, and organizational changes (e.g., number of ordered tests); and patient outcomes including patient health outcomes and patient-reported satisfaction. We added effectiveness results from the development phase of the toolkit where available.

### Study quality assessment

We used the Quality Improvement Minimum Quality Criteria Set (QI-MQCS) to assess studies [9]. The QI-MQCS is a 16-item scale designed for critical appraisal of quality improvement intervention publications; the domains are described in Additional file 2. The synthesis for the primary outcome integrates the appraisal finding; results for all included studies are documented in Additional file 2.

### Data synthesis and presentation

We documented the included studies in an evidence table (with supporting tables in the appendix) and summarized evaluation results in a narrative synthesis. Given the diversity of the identified studies, the quality of evidence assessment was limited to assessing inconsistency in study results across studies and study limitations of identified studies. The synthesis followed the key questions. Key question 1 was organized by the developed framework of components. Key question 2 was organized by outcome category: uptake and utility. Key question 3 was organized by provider outcomes, procedure/organizational results, and patient outcomes. The primary outcome of the review was patient health outcomes. The synthesis differentiated evidence from studies with concurrent and with historic comparator. For each toolkit, the evaluation of the intervention spread (i.e., using an available toolkit to disseminate practices and tools included in the toolkit) was also contrasted with initial results obtained in the organization where the toolkit had been first developed (where information was available).

## Results

### Review statistics

The electronic search for “toolkit” publications and a forward search for 156 specific toolkits (see Additional file 2) published by AHRQ, CMS, WHO, IHI, RWJF, AORN, ECRI, CDC, VA, or on the AHRQ Innovation Exchange identified 5209 citations. We obtained 661 citations as full text articles; of these, 77 studies were identified that met inclusion criteria (Fig. 1).

#### Study characteristics

Four included evaluations of groups randomized to an intervention or a control condition. Six studies provided a comparison to concurrent (non-randomized) control groups that did not participate in toolkit implementation. Forty-two studies presented pre- and post-intervention data for at least one outcome but did not include a concurrent comparator to account for secular trends independent of the intervention. Twenty-five studies reported only post-intervention data and provided no comparison to the status before or without the toolkit. Assessment methods and reported details varied widely and included online and written staff surveys, administrative data, medical chart review data, and web statistics.

The range of healthcare organizations involved in the evaluation varied widely from single hospital evaluations (10%) to studies with data on 325 institutions; and 22% of studies, often those that reported on web download statistics, did not report on the number of institutions. The number of participating staff members, often healthcare providers asked to use tools contained in the toolkit in clinical practice, ranged from 17 to 704, but the number of participants was only reported in 47% of studies. Of those studies reporting patient data, 59% reported the number of patients the data were based on; the number varied and ranged from 43 to 337,630.

Sixty-nine percent of included evaluations described elements in addition to the toolkit such as workshops and presentations to introduce the toolkit or the intervention promoted in the toolkit. The developer of the toolkit was part of the evaluation of the toolkit in more than half of the included studies (59%); toolkits were evaluated by independent study groups in 27% of studies (14% unclear).

Most evaluations were conducted in the USA (75%); other countries contributing to the study pool were Canada, the UK, Australia, Mongolia, and an international evaluation with multiple countries. In 34% of studies, the evaluation setting was a hospital; in 32%, toolkits were evaluated in primary care facilities; other organizations included community health centers, ambulatory care clinics, long-term care facility, specialty clinics (e.g., multiple sclerosis clinic), a hospice, and in some cases the characteristics were not reported.

The details of the included studies are shown in the evidence table (Table 1).

**Table 1:** Evidence table

Study ID Country Topic^a^	Setting *N* hospitals *N* providers *N* patients Study design	Toolkit name and components Other intervention evaluated in the study	Results Uptake Feasibility Provider Procedures Patient
Ashiru-Oredope 2016 [56] UK Antimicrobial stewardship (AMS)	Primary care and acute trusts # hospitals: 211 cinical commissioning groups (primary care), 146 acute trusts # providers: NR # patients: NR Post-only	Treat Antibiotics Responsibly, Guidance, Education, Tools (TARGET) (primary care) and Start Smart Then Focus (SSTF) (trusts) Implementation: TARGET: Guidance (local/national antibiotic treatment recommendations), suggested antibiotic practice audits. SSTF: Written materials Provider: TARGET: Educational materials and tools for providers to share with patients. SSTF: Examples of audit tools, review stickers, and drug charts Patient: Patient leaflets (Treating Your Infection), resources for clinical and waiting areas Other intervention: None	Uptake: Of the 82 responding groups, 60% had formally or informally reviewed TARGET and 13% had an action plan to implement AMS interventions recommended by TARGET. Fifty percent had implemented use of the patient information leaflet, 59% were using the TARGET educational presentation, 58% promoted TARGET during practice prescribing visits, 46% promoted the use of TARGET to GPs for use in CPD/revalidation. Groups that had reviewed TARGET were more likely to have implemented an action plan (OR 8.68, CI 1.06, 71.48, *p* = .044), and more likely to have implemented the use of the patient information leaflet (OR 4.38, CI 1.70, 11.27, *p* = .002). Of 100 responding trusts, 87% had reviewed national AMS toolkits, and 46% had implemented an action plan to deliver national toolkit AMS interventions. Acute trusts that had undertaken a review of SSTF were more likely to have implemented an action plan (OR 3.33, CI 1.00, 11.06, *p* = .050). Feasibility: NR Providers: NR Procedures: Few groups had implemented suggested audits or collated data in prevalence surveys; however, 69% groups had used local antibiotic audits within the past 2 years. The most frequent recommended audits in trust-wide point prevalence surveys included: adherence to guidelines of dose, route and duration (82%); clinical indication and treatment duration documented on drug chart (82%); and intravenous to oral switch at 48 h (49%). Other audits, such as review of prescription/evidence of documenting decision at 48 h and time to first dose in severe sepsis, were less commonly implemented (42% and 40% of acute trusts). Patients: NR
Jones 2017 [69] UK Antimicrobial stewardship (AMS)	Primary care # hospitals: 56 # providers: 269 (surveys), 24 (focus groups), 29 (interviews) # patients: NA Post-only	TARGET (Treat Antibiotics Responsibly; Guidance, Education, Tools) Antibiotics Toolkit Implementation: see Ashiru-Oredope, 2016 Provider: see Ashiru-Oredope, 2016 Patient: see Ashiru-Oredope, 2016 Other intervention: 1 h workshop covering AMR, guidance, how to optimize antibiotic prescribing, use of resources in the Toolkit, reflection on their own antibiotic prescribing data and some action planning. Workshop participants completed a five-point Likert scale eval	Uptake: Around half of GPs reported using the TARGET resources to varying degrees. Feasibility: Most GP staff and stakeholders described the TARGET Antibiotics Toolkit as a useful resource, which addressed their own prescribing behavior and patient expectations. They felt that it complemented existing efforts and was relevant to all practice staff in developing a consistent approach to patient enquiries about antimicrobials. Providers: 88% responded that the workshop helped them understand how to optimize antimicrobial prescribing, 88% responded that the workshop helped them to understand why responsible antimicrobial prescribing is important. All stakeholders were promoting its use. Procedures: The self-assessment checklist is a key resource that can be used for monitoring but was infrequently mentioned by participants. Patients: NR
Bender 2011 [19] US Asthma management	Primary care # hospitals: 58 # providers: 372 # patients: ~15,508 Pre-post	Colorado Asthma Toolkit Implementation: Training manual Provider: Spirometer Patient: Toolkit for patients, peak flow meter, guide for self-management support, telephone outreach enrollment form, asthma action plan, "understanding asthma" booklet, other educational materials Other intervention: On-site training/coaching, optional interactive voice response program for patients	Uptake: 98% of practices offered the toolkit took it up; median action plan use increased from 0% to 20% after coaching (53% of practices increased) Utility: NR Providers: NR Procedures: Patients with asthma using inhaled corticosteroids increased from median 25 to 50% (*p* < .0001), patients with an action plan 0 to 20% (*p* < .001), use of spirometry 0 to 40% (*p* <. 0001); cost of training each practice was $4194 Patients: NR
Taylor 2017 [87]; Tapp 2017 [108] US Asthma management	Community-based practices # hospitals: 6 # providers: NR # patients: 92 CT	Shared Decision Making (SDM) Toolkit (asthma management) Implementation: Asthma half day clinic flow, initial and follow-up scripts Provider: Spirometry technique, documentation template, follow-up patient information sheet, controller reliever posters, blank control dial, treatment goals and medication preferences form, prior authorization forms Patient: Facts about asthma, allergy information, smoking cessation information, severity and control dials, medication options, medication planner, general types of asthma medications, how to use your inhaler handouts, asthma diary Other intervention: A family medicine provider who was also part of the research team facilitated and tailored the implementation of the toolkit to the individual needs of each practice. The rollout typically consisted of hour-long weekly sessions with practice providers and staff. Adoption of the SDM intervention was reinforced with monthly meetings with representatives from participating practices and a refresher training at the end of year one.	Uptake: NR Feasibility: NR Providers: NR Procedures: Patient surveys asking, “Who made the treatment decision today?” were collected from 319 of the SDM toolkit visits. 87% reported a shared decision was made between patient and provider with 6% indicating the patient mostly made the decision, 74% indicating it was shared equally and 7% indicating the provider mostly made the decision. Patients: The toolkit intervention was associated with higher quality of life scores when compared to usual care (MD 0.9; CI 0.4–1.4). Models assessing the impact on individual quality of life domains (activities, emotions, symptoms) revealed similar results. Similarly, models examining differences in asthma control showed lower asthma control problems for children in the SDM toolkit intervention compared to usual care (MD − 0.9; CI − 1.6–0.2).
Nicolaidis 2016 [79] US Autism communication	Primary care # hospitals: NR # providers: 51 # patients: 259 Pre-post	Academic Autism Spectrum Partnership in Research and Education (AASPIRE) Healthcare toolkit Implementation: Autism information/diagnosis/referrals, legal and ethical information, other educational resources Provider: Autism Healthcare Accommodations Tool (AHAT), checklists and worksheets Patient: Personalized accommodations report, patient information, checklists, worksheets Other intervention: Patients recruited, sent AHAT to patient's PCP with a cover letter, information sheet about the study,	Uptake: NR Feasibility: Most PCPs rated it as moderately or very useful and indicated that they would recommend it to their patients. Most answers from PCPs to open-ended questions were positive and provided examples of the tool's utility. However, several PCPs noted that they already were doing what was recommended in the report, and two PCPs felt that they did not have time to implement accommodations. Providers: NR Procedures: NR Patients: Almost all autistic participants and supporters felt that the AHAT and the toolkit were easy to use, important, and useful. Over 90% said they would recommend the toolkit to a friend or their healthcare provider. The total number of barriers encountered by patients decreased significantly (*p* < .001). Participants’ self-efficacy in navigating the healthcare system also increased (*p* = .02). Participants described the toolkit as giving them a means to clarify and communicate their needs. Many participants felt that the toolkit validated their experience and empowered them to self-advocate more effectively. Participants also often gave examples of how the toolkit improved their self-efficacy, especially by helping them prepare for visits. Most participants were enthusiastic about how the AHAT report might affect their PCPs' behavior. Among the 43 patients who saw their PCP within the 1-month follow-up period, satisfaction with PCP communication improved significantly (*p* = .03). Participants described many concrete positive changes in providers or their staff. Several participants voiced frustration that their providers did not read the AHAT report or did not make any changes based on the report.
Chrisman 2011 [18] US Brain injury symptom management	Primary care # hospitals: NR # providers: 414 # patients: 0 RCT	Heads Up: Brain Injury in Your Practice Implementation: Booklet describing diagnosis and management, CD with additional resources, online resources such as posters and custamizable handouts available Provider: Palm card, Acute Concussion Evaluation (ACE) form Patient: Patient information in English and Spanish Other intervention: NR	Uptake: NR Utility: NR Providers: No difference in general concussion knowledge between intervention and control groups; intervention physicians less likely to recommend next day return to play after concussion (OR 0.31, CI 0.12, 0.76) Procedures: NR Patients: NR
Latsko 2015 [72] US Cancer care	Hospital # hospitals: NR # providers: 165 # patients: NR Post-only	Treating Myelodysplastic Syndrome (MDS) Toolkit Implementation: Summary of the patient survey, counseling guide Provider: Diagnostic spectrum reference card, mobile app Patient: Patient information sheets, free patient booklet order form Other intervention: None	Uptake: 24% of responding oncology nurses reported having a toolkit. Feasibility: NR Providers: Comparison of the responses of nurses in possession of the toolkit: For MDS Education, oncology nurses reported on education at the following times: During the diagnostic workup (53 vs 41%); at each follow-up appointment (75 vs 51%); prior to a change in treatment (85 vs 74%); at completion of treatment (53 vs 33%) and at the time of disease progression (78 vs 68%). For side effect education, respondents reported: prior to administration of each treatment (93 vs 86%); at each treatment appointment (75 vs 70%); at the completion of each treatment (50 vs. 39%); and during follow-up calls after treatment (45 vs 40%). Procedures: NR Patients: NR
Gulati 2015 [67] UK Cancer screening	Primary care # hospitals: 3374 practices that used toolkit between March 2012 and June 2013 # providers: 8163 users, 1002-1007 survey responders, 276 GPs making referrals # patients: NR Post-only	General Practitioner (GP) Skin Cancer Toolkit Implementation: NR Provider: Online resources including referral guidelines, real-life case histories, accredited quiz Patient: NR Other intervention: Promoted though regular email notifications to GPs and on the host home page	Uptake: Accessed by 8163 GPs 12586 times viewing 127036 page views and spending a median time of 4 min each (mean 5.37 min) over 255 days. 1/3 users used the site more than once; there were 6% more unique users and 23% more unique users than the next most popular education campaign on Doctors Net UK (DNUK) in 2012. Clinical case studies were used most commonly (3082 times by 2587 users), followed by the referral decision aid (2935 times) and the lesion recognition resource (2215 times). Feasibility: Focus grousp principal themes included that GPs considered themselves to be gatekeepers for referring suspicious lesions, but were concerned about avoiding unnecessary referrals; existing resources were useful, but prior dermatology teaching had been poor; the toolkit was useful for diagnosing skin lesion and allaying patient anxiety, but did not influence referral rates. Criticisms of the toolkit included lack of information and images for borderline and atypical skin lesions. Suggested improvements to the toolkit included more clinical images, online forums for discussing uncertain cases and a zoom function for clinical images. Providers: Reported confidence in recognizing different skin lesions suspicious of skin cancer was lower in 2013 compared to 2011 (*p* < 0.01) but there were no differences between toolkit users and non-users (*p* = 0.798). Confidence in knowledge of the appropriate referral pathways for malignant skin lesions was higher in respondents who used the toolkit (*p* < 0.01). There was no difference in perceived information and training about skin cancer recognition (*p* = 0.786). However, more respondents who had used and recalled using the toolkit said they had received adequate training and information compared to non-users (*p* < 0.05). The proportion of appropriate referrals increased from 21 to 32% (*p* < 0.0001). Procedures: Although the absolute numbers of urgent skin cancer referrals and melanoma and non-melanoma diagnoses increased, there were no significant changes in the number of urgent GP referrals for suspected skin cancer (*p* < 0.001), diagnoses of melanoma (*p* < 0.001) or diagnoses of non-melanoma skin cancer (*p* < 0.001) between the toolkit user and non-user groups. Patients: NR
Spruce 2012 [17] US Cancer screening	Primary care # hospitals: NR # providers: 30 out of 106 invited # patients: NR Post-only	Improving Colon Cancer Screening in Nevada with a Primary Care Toolkit Implementation: Sample chart audits, tracking sheets, decision aids, office strategies to improve screening, resources Provider: Phone scripts, care flow sheets, recommendations, algorithm Patient: Reminder and results letters, postcards Other intervention: NR	Uptake: NR Utility: All participants were very satisfied or satisfied with the overall usefulness of the toolkit, 97% were very satisfied with the educational content, all were very satisfied that the info was presented clearly, 83% were very satisfied with office strategies to improve screening, all were very satisfied with algorithms and tools Providers: 95% stated they would increase practice use of fecal immunochemical tests for those patients who are not eligible for or refuse a colonoscopy and would implement many of the toolkit recommendations Procedures: NR Patients: NR
Adsett 2014 [41] Australia Cardiac care	NR # hospitals: NR # providers: 340 # patients: NR Post-only	HEART (Heart Education Assessment Rehabilitation Toolkit) Online Implementation: Literature, references, links, videos, glossary Provider: Assessment tools, clinical calculators (e.g. BMI), educational materials and tips for initiating patient education Patient: Self-help resources Other intervention: NR	Uptake: NR Utility: Ratings of the toolkit on a five point scale (5 = strongly agree) were > 4 on 8 survey items related to content accuracy, ease of use, and relevance to practice Providers: NR Procedures: NR Patients: NR
Callard 2012 [11] UK Care quality	Hospital # hospitals: 2 trusts # providers: NR # patients: NR Post-only	15 Steps Challenge toolkit Implementation: Slide sets, briefing notes, action plan template Provider: NR Patient: Ward notice Other intervention: NR	Uptake: 2000 electronic versions have been downloaded from May to December 2012 Utility: NR Providers: NR Procedures: NR Patients: NR
Kemertzis 2018 [70] Australia Clinical decision making	Hospital # hospitals: 1 # providers: 59 # patients: 11 Pre-post	Fertility Preservation Toolkit Implementation: Instruction booklet Provider: Referral forms, information and consent forms, research information and consent forms Patient: Information sheets, booklet for patients and families, leaflets on fertility preservation options and brochure on treatments and fertility (male and female version) Other intervention: Education session to introduce toolkit	Uptake: 59 of 104 (56.7%) agreed to participate in the evaluation Feasibility: Clinicians were satisfied in 7/11 (64%) discussions, extremely satisfied or satisfied in 11/11 (100%). Reasons for dissatisfaction were missing documents within the toolkit, organization of the documents within the toolkit, and perception that there was too much written information which could overwhelm families and clinicians. The clinician perceived patient or family to have extremely or reasonably well understood the fertility preservation discussion in 10/11 (91%) cases and were perceived to be satisfied in all cases. Providers: There was an overall improvement in participant confidence levels in providing up-to-date fertility preservation information (*p* = 0.005). Procedures: There was an overall improvement in the provision of verbal (*p* = 0.003) and written (*p* = 0.02) information post toolkit use. Patients: NR
Pierce 2016 [81] US Critical care	Hospital # hospitals: 6 # providers: NR # patients: NR CT	Critical Care Protocol Toolkit (CCPT) Implementation: Written materials (list and description of steps involved in process), summary of key points Provider: None Patient: None Other intervention: NR	Uptake: The toolkit group followed all 9 steps in the toolkit and used 14.3 ideas from the toolkit on average. The non-toolkit group missed or weakly addressed 5.3 steps on average. Feasibility: The toolkit group experienced 13 barriers on average compared to 5.7 in the non-toolkit group (*p* = 0.512). Of the barriers encountered, the toolkit could have helped with barriers 62% of the time in the toolkit group compared to 77% in the non-toolkit group. Providers: NR Procedures: The control group missed or weakly addressed on average 3.3 of 9 key steps. The CCPT reduced implementation time from 56.4 days per step in the non-toolkit group to 46 days in the CCPT group (*p* = 0.327)NR Patients: NR
Han 2013 [31] US, UK, Australia, New Zealand, Canada, Ireland Depression care	Primary care # hospitals: NR # providers: 666 # patients: NR Post-only	MDPC (MacArthur Foundation Depression and Primary Care) Depression Toolkit Implementation: Slide presentations, training manuals, literature references, cost calculator Provider: Patient health questionnaire (PHQ-9), guides to diagnostic and treatment approaches, specialty care referral form Patient: Educational materials Other intervention: NR	Uptake: Since its launch, more than 20,000 users registered as members to read and download depression care resources Utility: Respondents generally rated resources as useful but ranged from 68% (PHQ-9) to 10% (cost calculator). Eight-four percent rated information on screening and diagnosis as good or excellent (70% for treatment, 66% for patient education materials, and 67% for care management material). Sixty-three percent were very confident in the content; more than half indicated that expert answers to clinical questions on depression, expert discussions research findings, and content on common mental illnesses would improve the tool Providers: 60% of respondents answered they had somewhat changed their practice after viewing the web site resources Procedures: NR Patients: NR
Gray 2017 [65] Canada Diabetes care	# hospitals: NR # providers: 462 (baseline), 132 (follow-up) # patients: NR Pre-post	Building Competency in Diabetes Education: Physical Activity and Exercise Implementation: see Fowles, 2014 Provider: see Fowles, 2014 Patient: see Fowles, 2014 Other intervention: Training workshops offered in which each component of the manual was presented, and participants were given time to practice instructions in motivational interviewing and how to perform and instruct patients in resistance exercises.	Uptake: NR Feasibility: Participants found the resources helpful (M 5.1, SD 1.49). Frequency analyses of the 7 specific resources provided in the toolkit revealed that brochures concerning resistance training were referred to on a regular basis during sessions with patients (58%). Less than half reported referring regularly to the remaining 6 resources (informational brochures, counseling worksheets, decision tree, at-a-glance summary sheets, data collection sheets, resistance training brochures). A large majority reported that they most often used the resources when working with patients who were inactive but were ready for or preparing for physical activity (80%). Reported challenges included: time (37%), patients’ resistance (36%), patients’ physical limitations (27%), patients’ personal barriers (23%), participants’ lack of expertise (16%), lack of physical-activity resources (12%), and other (24%). Analysis of the 4-point impact ranking scale revealed that the impact of the challenges was moderate (M 2.4, SD 0.82). Providers: Immediately following the workshop, particiapnts intended to implement the resources in their sessions with clients (M [median] 4.5/5, SD 0.64); 93% reported that they would very likely or definitely use the resources. Participants intended to increase the priority given to physical activity, employ the tools provided in the toolkit, incorporate resistance training into practice, and engage in novel ways to promote physical activity. The majority reported an increase in confidence across physical activity counseling. The most frequently reported areas were confidence in providing information and advice regarding the benefits of activity (86%); providing advice and instruction regarding resistance training (86%); and providing a physical activity program that accommodated patients’ individual needs or limitations (78%). Sixty-four percent reported an increase in confidence in their typical patients’ abilities to perform physical-activity behaviors appropriate for their fitness levels. The majority indicated that their confidence in making exercise referrals had not changed since the workshop training. Procedures: Prior to having attended the workshop, 58% included physical-activity content in more than half of their sessions, while 29% addressed physical activity in < 25% of patient sessions. In the sessions in which physical activity was discussed, 73% spent < 25% of the session on physical activity content, and 30% discussed physical activity for < 10% of each session. At the 8–12-month follow up, 66% included physical-activity content in more than half of their sessions, while only 18% addressed physical activity in < 25% of sessions. However, in the sessions in which physical activity was discussed, 78% spent < 25% of the session on this topic. 8 themes emerged: more frequently and confidently discussing physical activity in sessions (27%); increasing focus on resistance training (26%); providing patients with physical-activity procedures and written information (14%); feeling better equipped to assess current physical-activity levels (7%); assisting patients in working around barriers to being involved in physical activity (5%); recommending specific ctivities (4%); encouraging other health professionals to integrate physical activity into practice (4%) and other (12%). Patients: NR
Fowles 2014 [44] Canada Diabetes management	Community health center # hospitals: 7 # providers: NR # patients: 198 CT	Diabetes Building Competency in Diabetes Education: Physical Activity and Exercise Implementation: Resource manual, literature review Provider: Guidelines for risk stratification, assessments of readiness for exercise, referral process guide, clinical decision tree Patient: Sample exercise programs, goal setting worksheets, decisional balance sheets, informational brochures, online material, resistance exercise video Other intervention: 3-h training and 3-h regional workshop for diabetes educators	Uptake: NR Utility: NR Providers: Increased confidence in ability to provide physical activity and exercise counseling in intervention group (*p* < .001); greater knowledge about physical activity (*p* <. 03) but perceived physical activity counseling to be more difficult after receiving the trai Procedures: NR Patients: No significant difference in patient reported physical activity and exercise levels, efficacy perceptions, or mean glycated hemoglobin; no differences in relative use of medication or changes in medications or diet between toolkit and standard care groups
Alidina 2015 [55] US Elective delivery	Hospital # hospitals: 1 # providers: NR # patients: 1065 Pre-post	Elimination of Non-medically Indicated (Elective) Deliveries Before 39 Weeks Gestational Age Implementation: Written materials, decision support flow chart, scheduling flow chart, sample scheduling form Provider: Patient education talking points, patient education poster Patient: Patient education brochure, flyer Other intervention: NR	Uptake: Used toolkit to implement new scheduling processes. Feasibility: NR Providers: NR Procedures: In the study group there were 4 patients versus 42 patients in the control group (*p* < 0.0001) delivered between 37/0 and 38/6 weeks without an indication. Patients: There were 0 transfers to the NICU compared to 5 preintervention (*p* < .022) for non-medically indicated deliveries between 37/0 and 38/6 weeks.
Chesis 2015 [58] US Elective delivery	Hospital and staff obstetrician offices # hospitals: 1 # providers: NR # patients: NR Pre-post	Elimination of Non-medically Indicated (Elective) Deliveries Before 39 Weeks Gestational Age Implementation: see Alidina et al., 2015 Provider: see Alidina et al., 2015 Patient: see Alidina et al., 2015, flyer Other intervention: Additional measuring tool developed by the Advocate System Obstetric Safety Committee to help drive appropriate patient scheduling and data collection	Uptake: NR Feasibility: NR Providers: NR Procedures: Rate for non-medically indicated elective delivery was 25.0% pre-implementation in 2011; post-implementation by the end of 2011 it was 11%. In the 18 months prior to publication, it was 0.0%. Patients: NR
Clancy 2012 [13] US Emergency preparedness	Hospital # hospitals: 116 # providers: NR # patients: NR Post-only	NY State Department of Health Pediatric and Obstetric Emergency Preparedness Toolkit Implementation: Training material stratified by hospital type, links to online courses, educational material and clinical guidelines, glossary Provider: Safety checklists, triage algorithm, assessment tools, dosage guidelines Patient: Fact sheet for parents/caregivers Other intervention: NR	Uptake: 91% were aware of the toolkit, 86% had reviewed it Utility: Reasons for not appointing pediatric physician/nurse coordinators: implementation not started, no person available, cost Providers: NR Procedures: 1 year after toolkit distribution, 60% of facilities had appointed a pediatric physician coordinator, 49% a pediatric nurse coordinator. Toolkit review was not associated with the presence of an emergency management plan Patients: NR
Wyte-Lake 2016 [89] US Emergeycy preparedness	Home-based primary care # hospitals: NR # providers: 77 # patients: NR Post-only	Home-Based Primary Care/Home Health Agency Disaster Preparedness Toolkit Implementation: Written materials, source documents Provider: Checklists, suggestions, examples of tools Patient: NR Other intervention: Introduced over a national call for VHA HBPC program directors, posted to VA pulse, written invitation to participate in web-based toolkit evaluation, reminder emails and letters	Uptake: NR Feasibility: Of those respondents who found the toolkit very helpful (for clarity of design, comprehensiveness of information, and overall impression of the toolkit) approximately 60% had been part of the HBPC program for 5 years or less. The percentage of respondents who reported the toolkit to be helpful decreased as length of time in the HBPC program increased (22–25% for 6–10 years and 15–18% for ≥ 11 years). These results indicate that helpfulness of the toolkit was associated with fewer number of years with the HBPC program (*p* < 0.05). Length of time in the HBPC program manager role was not found to be associated with perceived helpfulness of the toolkit. On a 4-point Likert scale, respondents were asked if they agreed or disagreed that the topics covered in the toolkit were relevant to their preparedness protocol. Of those who implemented their disaster preparedness protocol more frequently (3–5 times/year or 1–2 times/year), two-thirds (66–67%) strongly agreed that the topics covered in the toolkit were relevant. Conversely, of those who implemented their protocol very infrequently or never, only 23% strongly agreed that the topics covered in the toolkit were relevant to their work (*p* < 0.05). When asked, How often do you see yourself using this toolkit?, 8% indicated that they will never use the toolkit. The rest indicated that they would use the toolkit moderately or extensively (data not shown). HBPC program representatives were asked to describe the types of support they would need to implement the toolkit. They suggested speaking with others who have implemented the toolkit, sharing it with leadership and hospital-wide committees, collaborating with local law enforcement and receiving online training, especially discipline-specific training. They also would appreciate reminders about the toolkit. Providers: NR Procedures: NR Patients: NR
Henry 2012 [14] Mongolia Emergency/surgery capacity	Hospital # hospitals: 338 (hospitals in 12 provinces) # providers: NR # patients: NR Pre-post	Integrated Management of Emergency and Essential Surgical Care (IMEESC) toolkit Implementation: WHO recommendations for minimum standards to improve quality and safety, equipment lists and needs assessment, manual, trainer's guide, detailed training material, model training workshops, research tool Provider: Best practice protocols, care guides, diagnostic tools, safety checklists, link to videos on surgical procedures Patient: NR Other intervention: 5-day training program and site visits given by participants in WHO Training of Trainers workshop	Uptake: 67% of provinces and 53% of hospitals implemented the program Utility: NR Providers: NR Procedures: Increase in number of surgical procedures performed, decrease in number of surgical procedures referred to other facilities Patients: NR
Cox 2017 [60] UK End-of-life care	Care homes # hospitals: 6 # providers: 78 (pre), 103 (post) # patients: NR Pre-post	End of Life Care Toolkit (part of Care Homes and hOspitals Innovating Collaboratively to increase End of life care options [CHOICE] Project) Implementation: Written materials Provider: Written materials Patient: NR Other intervention: Three training sessions (1 h each) in each care home	Uptake: NR Feasibility: NR Providers: After the intervention, there was a trend for staff to report feeling more supported in terms of emotional and clinical support in the care home and feeling able to source external support GP/district nurse; Q11 hospice/palliative care nurse), even out of hours. The results suggested confidence in ability to discuss death and dying with residents was lower post-intervention, although this change in confidence did not reach statistical significance (*p* ≥ .05). Mean scores suggest interventions did not affect staff confidence in terms of discussing death and dying with relatives, identifying end of life, or the creation of EoLC plans. Staff confidence in managing each of the 24 end of life symptoms including pain, anxiety, nausea and vomiting, and mouth care increased post-intervention, however, this trend did not reach statistical significance. Procedures: NR Patients: A comparison of a 5-month period before and after the intervention indicated a 59% reduction in the number of residents who died in the local NHS hospital in comparison to a 21% reduction from the comparison care homes.
Carroll 2012 [16] US Fall prevention	Hospital # hospitals: 8 # providers: NR # patients: 364 RCT	Fall TIPS (Tailoring Interventions for Patient Safety) Implementation: NR Provider: Fall risk assessment scale, individualized bed poster, plan of care Patient: Peronalized patient/family education handout Other intervention: NR	Uptake: NR Utility: NR Providers: NR Procedures: Patients on the intervention units were more likely to have fall risk documented (89 vs 64%; *p* < .0001); there were significantly more comprehensive plans of care for the patients on the interventions documented but no difference were found regarding documentation of completed interventions Patients: NR
Coe 2017 [59] US Fall prevention	Clinical and community-based organizations # hospitals: 23 clinical and 27 community-based organizations # providers: NR # patients: 20317 Post-only	Stopping Elderly Accidents, Deaths, and Injuries (STEADI) Implementation: Stories about falls prevention programs and successes Provider: Tests, fact sheets, case studies, additional resources Patient: Educational materials and brochures Other intervention: Statewide falls prevention learning collaborative (includes full day statewide learning sessions twice per year, expert-led falls prevention webinars are offered several times per year, ongoing trainings on each intervention in multiple modalities), referral of qualified patients to community-based interventions (Tai Chi, Matter of Balance [MoB], and Assisted Home Safety Assessment and Modification [AHSA]).	Uptake: Over a period of 21 months of implementation clinical sites assessed patients using the STEADI protocol and referred 4726 individuals to PWTF community sites for falls prevention interventions. Of those, 44% enrolled in the PWTF-sponsored community interventions and of those enrolled 45% completed the interventions. Organizations also recruit individuals directly. There were 2256 “walk-ins” and 989 “completers.” Overall, > 4359 individuals enrolled, and ~ 1945 completed interventions. Feasibility: Stopping Elderly Accidents, Deaths and Injuries implementation was challenging for the primary care sites as falls risk assessment was a new area and requires systems change. PWTF sites experienced challenges such as securing support from senior leadership and clinical staff; the lack of reimbursement for specific clinical components; no data fields in EMR to capture or assess falls assessments; and lack of workflows and processes for implementing STEADI. Clinical and community staff faced challenges in referring and enrolling individuals into community interventions due to reluctance due to the time commitment, lack of understanding of risk, and unfamiliarity with programs or organizations running the programs. Partnerships tested multiple strategies to overcome these issues. Providers: NR Procedures: During a 9-month period 48% of patients were screened for falls risk and 30% of those who screened positive received an evaluation of their gait, strength and balance. Of those who screened positive, 37% received a plan of care and a multifactorial clinical risk assessment. Of the patients screened, 6% received referrals to a community falls prevention intervention. Of those referred, 44% enrolled in the community interventions. Patients: NR
Dykes 2009 [61]; Zuyev 2011 [109] US Fall prevention	Hospital # hospitals: 4 # providers: NR # patients: 685 Pre-post	Fall TIPS (Tailoring Interventions for Patient Safety) Toolkit Implementation: see Carroll et al. (2012) Provider: see Carroll et al. (2012) Patient: see Carroll et al. (2012) Other intervention: NR	Uptake: Adherence with toolkit adoption measures ranges from 72% (bed poster) to 97% (fall risk assessment completed), varying by site and component Utility: Feedback from end users is positive Providers: NR Procedures: Mean number of fall risk assessments completed per day increased from 1.7 to 2.0 one month after implementation (*p* < .003) Patients: The mean fall rate decreased from 3.28 to 2.80 falls per 1000 patient-days post intervention and the mean fall with injury rate decreased from 1.00 to 0.54 per 1000 patient-days.
Fisher 2013 [10] UK Fall prevention	Hospice # hospitals: 1 # providers: NR # patients: NR Pre-post	Falls prevention and management toolkit Implementation: Policy document (framework for multi-factorial assessment and definition of fall) Provider: Assessment and care plan tool, incident report form Patient: NR Other intervention: NR	Uptake: There are hospices that continue to use the toolkit, some have made small or substantial adaptations to meet local requirements Utility: NR Providers: NR Procedures: NR Patients: The author's hospice reduced falls to 4.3 per occupied bed per year in 2011–2012 from 5.1 in 2007
Stalhandske 2008 [52] US Fall prevention	Hospital # hospitals: 65 out of 70 initial volunteers # providers: 42 # patients: NR Pre-post	National Falls Toolkit Implementation: CD, informational brochures, posters, flyers, sample buttons for identifying advocates or resources, online resources Provider: Morse Fall Scale pocket card, video on performing a balance assessments Patient: Videos on hip protectors for patients/caregivers Other intervention: Falls data monitoring	Uptake: NR Utility: NR Providers: NR Procedures: Changed or implemented a fall risk assessment (14%), changed the system of tracking falls data (12%), honed in on an area of vulnerability (10%), changed or implemented a falls policy (7%), changed or implemented a falls team (7%), increased use of documentation or falls prevention interventions (5%), became more proactive in falls prevention (5%), other ways such as implementing specific interventions (21%) Patients: Over the course of the 2 years, there was a reduction in major injuries (e.g., 64% in behavioral health setting); fall rate remained relatively stable
Ryan 2013 [30] Canada Geriatric care	Primary care # hospitals: 181 out of 220 invited # providers: NR # patients: NR Post-only	Geriatrics, Interprofessional Practice, and Interorganizational Collaboration (GiiC) Toolkit Implementation: Topic overviews, FAQs, materials for broader reading, interprofessional and interorganizational collaboration elements (eg, team assessment, recognizing states of team development, team problem solving, understanding organizational outcome expectations, v Provider: Pocket guides, algorithms and clinical tools, self-directed learning materials downloadable to personal computers or flash drives Patient: Bilingual patient handouts Other intervention: 16-h “train-the-facilitator” workshops, small-group discussions to support implementation, 2–4 months coaching from consultants, 6 months refresher day and practice review, annual meeting	Uptake: 79% of identified family health teams and 85% of community health centers participated in the initiative; the toolkit has been downloaded 41,556 times; 48% of participants reported moderate or a lot of change in their team's care of frail seniors Utility: NR Providers: Participant ratings of knowledge gain and confidence in geriatric competencies was 3.46 (1 not at all, 5 a great deal). Participant ratings of perceived learning and facilitator confidence were 3.67, and 4.15 in interprofessional and 4.02 Procedures: NR Patients: NR
Dore 2013 [34] US Health literacy	Rheumatology practice # hospitals: NR # providers: 18 # patients: NR Pre-post	Health Literacy Universal Precautions Toolkit for Rheumatology (HLUPTK-R) Implementation: Material on forming teams, raising awareness, organizational assessment, Plan-Do-Study-Act worksheets, educational material, videos Provider: Quick start guide for providers, medication dosing form, checklists, tips for addressing language differences and patient education Patient: Links to online resources for patients Other intervention: 20-min introductory presentation	Uptake: 72% participants stated HLUPTK-R Qick Start techniques were incorporated into their practice Utility: 54% of users thought that incorporating health literacy techniques added time to the patient's visit, but all thought the time was worthwhile; all believed the techniques were helpful in their practice Providers: 77% of users agreed that their knowledge of health literacy was improved and that incorporating the quick start techniques had a positive impact on patient care Procedures: All toolkit users used the encouraging questions technique, 62% used the teach-back method, 23% used the medication review technique Patients: NR
Mabachi 2016 [75] US Health literacy	Primary care # hospitals: 12 practices # providers: 3 individuals/site # patients: NR Post-only	Health Literacy Universal Precautions (HLUP) Toolkit Implementation: NR Provider: NR Patient: NR Other intervention: Practices identified and ranked the top four of 11 priority tools they wanted to implement; practices were assigned to implement two tools. Technical assistance providers from the research teams conducted chec-in calls at 2 ,4, 8, and 16 weeks.	Uptake: Practices used the Toolkit “flexibly.” They did not always implement all portions of their assigned tools. Practices found that some tools were best implemented in tandem and recognized the efficiencies in implementing them in this manner. Feasibility: Specific implementation barriers were noted by the participating practices, including (1) competing demands/staff capacity, (2) bureaucratic challenges, (3) technological challenges, (4) limited quality improvement experience, and (5) limited support from leadership. Linking health literacy implementation activities to other practice-wide QI initiatives (e.g., patient-centered medical home accreditation) raised staff awareness and increased engagement. Providers: While practices worked independently on toolkit implementation, they benefited from having external support and accountability. Three-fourth of the practices reported that they plan to continue to use the Toolkit as a resource to guide their health literacy-related QI work. 8/12 practices indicated they would continue with their 2 assigned tools and the improvement implemented during the study period. Procedures: NR Patients: NR
Koelling 2006 [28] US Heart failure	Hospital # hospitals: 14 # providers: NR # patients: 1806 CT	Guidelines Applied in Practice - Heart Failure (GAP-HF) Tool Kit Implementation: Quality performance charts Provider: Heart failure standard admission orders, heart failure specific clinical pathway, heart failure patient discharge contract Patient: Self-management diary Other intervention: 6 monthly learning sessions	Uptake: NR Utility: NR Providers: NR Procedures: NR Patients: Baseline-adjusted 30-day readmission rate was statistically reduced (*p* = .003) but not 30-day mortality (*p* =. 101) comparing intervention and control group
Perumalswami 2016 [80] US Hepatitis C care	Primary care # hospitals: NR # providers: NR # patients: NR Post-only	HepCure (Hepatitis C education and patient engagement) Implementation: NR Provider: Open access toolkit (a dashboard) that enhances providers’ ability to deliver guideline-based HCV care; and a tele-education platform for medical providers Patient: Linked patient app that provides education, medication reminders, and a platform for tracking adherence and symptoms Other intervention: None	Uptake: Weekly tele-education sessions have been conducted 57 times since February 2015, with 322 unique attendees and an average of 22 (plus or minus 9) attendees a week. Five hundred forty-six downloads of patient app from November 2014 to May 2016. Feasibility: NR Providers: NR Procedures: NR Patients: NR
Adams 2014 [45] US Hospital readmission	Hospital # hospitals: 1 # providers: NR # patients: 336 Pre-post	Project Re-Engineered Discharge (Project RED) Toolkit Implementation: Detailed implementation guide, workbooks Provider: After Hospital Care Plan forms, post-discharge follow-up phone call script Patient: Booklet, checklist for appointments Other intervention: Patient discharge questionnaire, contact sheets (in English and Spanish)	Uptake: NR Utility: NR Providers: NR Procedures: 94–95% of patients reported having received written information regarding medications, their condition, and when to seek medical attention Patients: Re-admissions were reduced by 32%; 99–100% of patients reported knowing their medication regiment and when to call the doctor or seek emergency care post-intervention
Mitchell 20015 [78] US Hospital readmission	Hospital # hospitals: 10 # providers: NR # patients: NR Pre-post	Project Re-Engineered Discharge (Project RED) Toolkit Implementation: Detailed implementation guide, workbooks Provider: After Hospital Care Plan forms, post-discharge follow-up phone call script Patient: Booklet, checklist for appointments Other intervention: 8-hour training, monthly telephone-based technical assistance calls for 1 year	Uptake: 7/10 successfully implemented the RED program as planned. Eight hospitals chose to initiate RED implementation in 1 or 2 units or wards and/or for patients with a particular diagnosis (i.e.,CHF patients only). Feasibility: The 7 implementing hospitals had the following common features: highly visible commitment from senior leadership, empowered interprofessional implementation team, established methods for sharing results and assessing accountability, buy-in from staff and stakeholders, and flexible in-house IT support. Nine of the participating hospitals implemented a site-specific adaptation of the RED protocol during the study period. All 10 hospitals revised the 2-day postdischarge telephone call script included in the RED Toolkit. Providers: Members of the implementation teams believed that the RED processes enhanced patient care, provided tools to help patients better manage their medical conditions, and had an important impact on job satisfaction, staff morale, and engagement. Procedures: Four hospitals did not hire personnel to perform RED responsibilities and instead used unit nurses for discharge education; two teams used nonclinical personnel or third-party vendors to conduct the 2-day post-discharge phone call. Patients: All 7 hospitals reported modest reductions in 30-day readmissions for at least one of the 3 diagnostic areas targeted by CMS for payment penalties (congestive heart failure [CHF], acute myocardial infarction [AMI], and pneumonia [PNA]). All but 2 hospitals reported a 0.5% or greater reduction in 30-day all-cause readmissions after the implementation. 5 hospitals achieved a greater net decrease in readmission rates than the national average for CHF patients, 4 surpassed the national average decrease for AMI readmissions, and 5 exceeded the national average decrease for PNA readmissions.
Enfield 2014 [40] US Hospital-acquired infections	Hospital # hospitals: 1 # providers: NR # patients: NR Pre-post	Center for Disease Control and Prevention's Carbapenem-resistant Enterobacteriaceae (CRE) Toolkit Implementation: Research overview, material to raise awareness, implementation strategies (e.g., regional approach to CRE), organizational risk assessment tool, references Provider: Laboratory standards chart, care algorithm Patient: NR Other intervention: Limited access to rooms and common areas, terminally clean room policy enhanced, monitoring of environmental cleaning and feedback	Uptake: All components implemented, but some were already in place before the intervention Utility: NR Providers: NR Procedures: Compliance with hand hygiene increased from 71% before to 86% after (*p* < 0.001); surface testing revealed 39% of tested items exceeding threshold levels before and 12% after (with 6% and 14% in subsequent periods, showing a sustained effect) Patients: Before, incidence rate of CRE was 7.77 cases/1000 patient-days, after 1.22 cases/1000 patient-days (*p* = .001); rate of extensively drug-resistant Acinetobacter baumannii (XDR-BA) was 6.79 cases/1000 patient-days before, after no cases were identified
Randle 2006 [26] UK Hospital-acquired infections	Hospital # hospitals: 6 # providers: 127 # patients: 43 Pre-post	Clean-Your-Hands Campaign Toolkit Implementation: Educational material, posters for staff and patients, marketing materials (aprons, badges, etc.), evaluation tool Provider: Alcohol hand rubs Patient: Leaflet, posters Other intervention: Campaign project manager trained pilot teams in evaluation tool use	Uptake: NR Utility: Respondents indicated the framework for implementation was helpful and the major success element were the alcohol hand rubs Providers: 70% of nurses and 60% of doctors agreed that the presence of wipes encouraged them to clean their hands, 76–84% indicated posters made staff think about their hand hygiene, 74% indicated they cleaned their hands more frequently Procedures: Compliance with hand cleaning increased from 32% (before toolkit) to 41% at 3 months and 63% at 6 months Patients: 16/43 patients had asked staff to clean their hands, 35/43 found posters etc. useful
Septimus 2016 [84]; Huang 2013 [91] US Hospital-acquired infections	Hospital # hospitals: 136 ICUs in 95 hospitals # providers: NR # patients: 305583 admissions (pre-period)/ 102220 (post) Pre-post	Universal ICU Decolonization Toolkit: An Enhanced Protocol Implementation: Protocol overview, scientific rationale, flow chart, readiness assessment and FAQ, training and educational materials, chlorohexane bathing skills assessment Provider: Nursing protocol, safety information Patient: NR Other intervention: Five coaching calls	Uptake: NR Feasibility: Challenges identified included concerns about mupirocin resistance and questions about peer review of the original trial results, Most facilities were able to easily implement daily CHG bathing, as this practice, fit within normal nursing work flow and did not require a physician order. Providers: NR Procedures: NR Patients: The raw CLABSI rate (CLABSI events divided by number of central line–day) dropped from 1.1/1000 to 0.87/1000 central line–days postintervention. There were 672 CLABSIs per 587 891 central line–days in the 24-month preintervention period, and 181 CLABSIs per 208 175 central line–days in the 8-month post-intervention period. After implementation, the rate of CLABSI decreased by 23.5% (CI 9.8–35.1%; *p* = .001).
Kuhlman 2014 [47] US Infant safe sleep	Pediatric and obstetric clinics # hospitals: 2 # providers: NR # patients: 309 Post-only	Safe Sleep Toolkit Implementation: NR Provider: Brief healthcare provider script Patient: Parental checklist, nationally available resources (links to videos, posters, brochures, door hangers) Other intervention: NR	Uptake: NR Utility: NR Providers: NR Procedures: Providers engaged in discussion regarding safe sleep with most parents who reported intentions/behavior in opposition to the recommendations for safe sleep Patients: NR
Haley 2015 [68] US Kidney disease care	Primary care and nephrology practices # hospitals: 9 primary care and 5 nephrology practices # providers: 25 pre, 24 post interviews # patients: 292 chart audits Pre-post	Advanced Chronic Kidney Disease (CKD) Patient Management Toolkit Implementation: Guide to tool selection, patient identification tools, patient management tools, physician education materials. slide presentation, clinical practice guidelines, awareness letter, Provider: Identification and action plan card, identification and action plan poster, glomerular filtration rate (GFR) calculator, CKD chart flags/stickers, referring physician faxback form, CKD post-consult letters, advanced CKD management flow sheet and algorithm Patient: CKD patient diary, CKD patient education resources, venipuncture reminder card, vascular access passport Other intervention: Education sessions at each site, made modifications to some tools and one additional tool created (CKD Screening Protocol/When to Refer)	Uptake: Site champions and physician leaders were contacted 3 years after completion of the study and asked whether the improvements in awareness of CKD, communication, the referral process, and comanagement of patients with CKD had been sustained and whether tools were still in use. Of those who had remained active in their respective practices over that time frame, 5 responded with 14 of 15 answers being affirmative. Feasibility: Increased communication between practices was associated with enhanced satisfaction scores. On a Likert scale, satisfaction with comanagement reported by nephrologists improved from 2.6 to 4.3. Corresponding satisfaction levels of PCPs were 4.3 and 4.7. Of the 16 respondents with preimplementation levels less than “satisfied,” 15 noted improvement, with 6 improving from “somewhat unsatisfied” to “satisfied” or “very satisfied.” Providers: Preimplementation, few practices reported familiarity with CKD clinical practice guidelines, and CKD screening was limited mostly to diabetic patients. Postimplementation, all practices reported increased awareness of risk factors for kidney disease. Pre-implementation, few used specific triggers for nephrology referrals, although several cited creatinine level. Then, 2.0 mg/dL or when dialysis questions arose. Postimplementation transcripts revealed increased consistency of referral timing, with practices providing more vigilant monitoring of high-risk patients: managing CKD up to stage 3 and all reporting referral by stage 4. Several nephrologists and their site champions noted the need for timely nephrology appointments. Postintervention nephrology interviews revealed heightened attention to communication and comanagement. Practices reported the project altered the content of nephrology postconsult letters, advanced comanagement goals, and improved teamwork among office staff. Procedures: At the outset, care processes and mechanisms varied among practices. Postimplementation improvement was observed for CKD identification, referral, and communication and execution of comanagement plans. 166/171 tasks and 124/144 subtasks were in place postimplementation, compared with 78 and 51 pre-implementation. Nephrology practices likewise improved postimplementation, particularly in their referral processes and communication. Postintervention questionnaires confirmed that patients with CKD were being referred earlier—none later than stage 4. Analysis of audits revealed improvement in GFR documentation (p=.01); most performed well with respect to ordering creatinine levels within 1 year for these high-risk patients preimplementation and all postimplementation (*p* = .2). The referral rate of patients with CKD was 24% pre-implementation and 39% postimplementation (*p* = .4). 4/7 showed an average 45% increase and a 5th showed a 100% increase in referral of patients with CKD stage 4. The rate of patients with GFRs ≤ 30 mL/min who were referred to nephrology was higher than the overall rate of referral of patients with CKD and did not increase (*p* = .7); the percentage of those with GFRs ≤ 30 mL/min was similar pre- and postimplementation (*p* = .3). Although there were too few practices for a formal comparison, those consistently using all tools performed better in terms of achieving project goals or improvements than those that did not. Patients: NR
Fernald 2015 [63] US Medical errors	Primary care # hospitals: 24 recruited, 22 actively participated # providers: NR # patients: NR Pre-post	Quality Improvement for Laboratory Testing Processes in Primary Care: Implementation Guide and Toolkit Implementation: Written materials, priorities worksheet, process mapping guide and example Provider: NR Patient: NR Other intervention: Initial site visit to introduce toolkit and study requirements and conduct initial process mapping and observation activity	Uptake: 20/22 actively participating practices were able to initiate a laboratory testing improvement process using the toolkit as a guide; 4 practices completed their activities withing the 6-week time period allotted, but many continued their effort after the study period. Feasibility: Comments on the toolkit were consistently positive, noting that the toolkit was straightforward, organized helpfully, and provided good talking points to align staff and providers. The process mapping exercise was very helpful. Providers: Practices rapidly acknowledged that ongoing problems exist in laboratory testing processes, that they needed help addressing these problems, and that they faced challenges in finding patient-centered solutions compatible with practice priorities and available resources. Procedures: NR Patients: NR
Leape 2006 [27] US Medical errors	Hospital # hospitals: 58 # providers: 76 # patients: NR Post-only	Reconciling Medications (RM) Toolkit and Communicating Critical Test Results (CCTR) Toolkit Implementation: Safe practice recommendations, implementation strategies, sample forms, sample flow charts, sample policies, example 'failure modes effects and criticality analysis' (FMEC), data collection tool, spreadsheet to track progress, example audit tool, workshe Provider: Test tracking log sheet, communication tools for reporting critical test results, reconciling medication form, patient medication card Patient: NR Other intervention: Collaborative groups met 4× over 18 months	Uptake: 88% of hospitals participated in one or both collaboratives; of 58 participating hospitals, 50 enrolled teams for RM and 40 enrolled teams for CCTR; 10–15% of teams were unable to get beyond Utility: 91% of RM teams and 75% of CCTR teams found implementing the recommended practices difficult; human and institutional factors were cited as more significant barriers than costs Providers: NR Procedures: NR Patients: NR
Mueller 2013 [49] US Medication management	Hospital # hospitals: 2 # providers: NR # patients: NR Post-only	Medication Reconciliation Implementation toolkit Implementation: Educational and instructional materials including teach-back technique; didactic slide deck, videos, and case study for role-playing to practice best possible medication history taking; return on investment spreadsheet; process map Provider: Pocket cards, discharge instructions, medication reconciliation forms, vendor lists, social marketing materials, AHRQ pharmacy health literacy assessment tool Patient: NR Other intervention: Formal training program or automated screening tool	Uptake: NR Utility: Limited resources were cited as a challenge to implementation; positive feedback on videos, other implementation tools less positively rated Providers: Perception of the study’s impact on patient outcomes to date varied by site, site 1 was more positive about the perceived impact on the quality and efficiency of medication reconciliation Procedures: NR Patients: NR
McHugo 2007 [38] US Mental health	Community mental health center # hospitals: 53 centers approached, 49 completed # providers: NR # patients: NR Pre-post	Evidence-Based Practices Implementation Resource Kits Implementation: User's guide, implementation tips, introductory videos, slide presentations, brochures, fidelity scales with protocols Provider: Practice demonstration video and workbook Patient: NR Other intervention: Consultant-trainers provided training and clinical supervision to program leaders and implementers	Uptake: 92% sites completed the project Utility: NR Providers: NR Procedures: 55% scored at least 4/5 on evidence-based practice fidelity scale at 2 years Patients: NR
MacDonald-Wilson 2017 [110] US Mental health decision support	Community mental health centers # hospitals: 52 # providers: NR # patients: NR Time series	Toolkit: Decision Support Implementation: Viewer’s guide, video tutorials, guide to reliable information resources, leadership team pledge Provider: Decisional balance worksheet, affirmation posters, adoption milestones and worksheets Patient: Information on self-care strategies Other intervention: 12-month learning collaborative with monthly webinars and 3 in-person learning sessions, electronic workbooks to collect process data, implementation milestones, and quality improvement activities through the Plan, Do, Study, Act process.	Uptake: Participation was high and remained high over time, 88% of CMHCs attended monthly support calls, 80% completed a PDSA cycle each month, and 80% delivered a completed workbook on time each month. A total of 469 PDSAs were completed over the collaborative ranging from 2 to 38 PDSAs per agency with the expectation of 11 PDSAs per agency. The majority of the PDSA content focused on improvements around access and training of staff on the Recovery Library and using the Decisional Balance Worksheet. Fewer PDSAs focused on improvements to the quality improvement process within agencies or general provision of decision support in care. 52% completed the tenth and final implementation milestone,.a large percent reached milestone eight (17%) and milestone nine (17%). Progress in meeting the goal for the process aim improved significantly over time (*p* < .0001). The average percent of staff meeting either the Research or Support process aim in the first quarter of the collaborative as reported by CMHCs was 38% which progressed steadily over the next three quarters, 65, 69, 75%, respectively. Feasibility: Across CMHCs, the importance of the agency in supporting implementation in order to ensure success was evident. Despite the majority of activity and responsibility of the learning collaborative placed on the QIT, staff members emphasized the importance of a top down approach in which success of implementation of new practices is more likely if agency leadership is involved. Providers: 31% of providers rated themselves a 4.5 (indication that practices were moving toward sustainability) and 29% rated themselves 5.0 (all project goals completed; organizational changes permanent). For the outcome aims of the learning collaborative, progress in meeting the goal for high ratings of confidence, Involvement, and competence by individuals in service improved (*p* < .0001). The percent service users meeting goal at baseline was 47% which increased over the next 4 quarters (49, 56, 59, and 64%). Competence in supporting shared treatment decisions was higher than confidence (*p* < .001) and involvement (*p* = .01). Confidence in accessing reliable health information over the internet was lower than ratings of involvement (*p* = .02). Procedures: NR Patients: NR
Miller 2014 [43] US Multiple sclerosis symptom management	Multiple Sclerosis clinic # hospitals: 10 # providers: 37 # patients: 405 Pre-post	Toolkit: Constructing an Adaptive Care Model for the Management of Disease-Related Symptoms Throughout the Course of Multiple Sclerosis Implementation: Implementation instructions and tips; sample data collection form; slide deck Provider: Clinical management algorithm, clinical practice guidelines, best practices for symptom management, online continuing medical education activities Patient: NR Other intervention: Training for intervention heads, planning meetings, live workshop	Uptake: NR Utility: Providers reported time and personnel constraints as a barrier to implementation, providers reported the intervention allowed for comprehensive management of the disease, and improved patient care overall Providers: Providers reported increased awareness of MS symptoms Procedures: 6% (*p* = .003) improvement in documented symptom assessment for mobility impairment/falls and 10% (*p* < .001) for spasticity assessment documentation; documented care plan performance measures improved for fatigue (13%, *p* = .007) and mobility impairment/falls (13%, .040). Use of the timed 25-foot walk test increased from 25 to 46% (*p* < .001), use of clinic questionnaire for spasticity improved from 3 to 9% (*p* < .001). Use of behavioral modifications, pharmacotherapy, and physical therapy remained unchanged Patients: NR
Guillory 2017 [66] US Newborn screening	Hospitals # hospitals: 13 # providers: 117 physicians, hospital administrators, and other health care professionals, 215 nurses # patients: 11322 Pre-post	Critical Congenital Heart Deiseas (CCHD) Toolkit Implementation: PP presentation for physicians, PP presentation for nurses, sample nursery policy, Taryn's Story (4 minute video script), Lifesaving Newborn Screen (30 s PSA script) Provider: Algorithm card, sample physician order, sample screening log, wall poster for newborn nursery Patient: Brochure (English/Spanish) for positive screen, brochure (English/Spanish) for families Other intervention: A hospital nurse champion (HNC) was identified for each facility to serve as a leader, with responsibilities of training personnel, facilitating implementation, and identifying and addressing barriers. Nurse educators and HNCs conducted an accredited, 1-hour training session at each hospital to ensure standardization of education for the newborn nursing staff.	Uptake: NR Feasibility: NR Providers: The pretest average was 71% and improved to 92.5% (*p* < .0001). In hospitals with CCHD NBS implemented prior to TxPOP, nurses’ pretest scores showed a relative lack of knowledge on the subject. In all trainings, questions requiring application of the clinical algorithm were missed most frequently, particularly on the pretest. Procedures: There were 1236 admissions to neonatal intensive care unit (NICU) and 11,710 admissions to newborn nursery or mother/baby unit, with 39 screened prior to 24h, 239 transferred from the newborn nursery before 24h, 32 with echocardiograms (ECHO) for other reasons, 3 parental refusals, and 75 with unknown screening status. 97% were screened after 24 h of age as recommended. There were no missed cases of CCHD. Missed screenings opportunities represented 0.66% of eligible patient encounters. Most occurred in the early phase of implementation of the project. The causes for missed screens, such as lack of awareness, lost probes, process problems, and timing of screening were collected, addressing these issues resulted in improvement in overall screening rates, from 97 to 99% (*p* < .0001). Patients: Eleven newborns had a positive screen. One patient was confirmed with CCHD (tricuspid atresia with hypoplastic right heart). Other important nontargeted diagnoses included seizure, abdominal distension, and other noncritical cardiac conditions. Two newborns were found to be healthy. Three were transferred from the birthing hospital to an urban facility for high level of care.
Dobbins 2005 [29] Canada Nursing best practices	NR # hospitals: 11 # providers: 41 # patients: NR Post-only	Toolkit: Implementation of Best Practice Guidelines Implementation: Self-assessment guide, action plan template, expense worksheet, stakeholder scenario worksheet, barriers and facilitators resources, guide to social marketing, sustainability action plan Provider: NR Patient: NR Other intervention: 1-day launch workshop	Uptake: 85% respondents reported reading the toolkit, 43% of users completed the worksheets, > 1000 toolkit copies requested, > 1600 nurses have signed up for workshop Utility: 85% of those who read the toolkit found it to be helpful during implementation Providers: NR Procedures: NR Patients: NR
Main 2017 [77] US Obstetric care	Hospital # hospitals: 147 # providers: NR # patients: 337630 CT	Comprehensive quality improvement tool kit for hemorrhage Implementation: Obstetric Hemorrhage Safety Bundle, Rounding/Huddles Templates, Placenta Accreta Protocol Sample, slide decks/presentations/webinars, sample forms, resource guide, Code C materials Provider: Debrief forms, evaluation form Patient: Patient, family, and staff support bundle Other intervention: Physician and nurse pairs mentored groups of 5–8 hospitals (groups were often geographic or system based). The mentors were not from the facilities they supported and served as facilitators leading the monthly telephone calls, providing small group leadership and personal accountability. A CMQCC staff member also supported the mentor groups and attended all telephone calls to coordinate and share lessons and ideas from all the groups. In-person full-day meetings for learning and sharing involving all hospital teams were held toward the beginning and the end of the project. Additionally, hospitals were encouraged to share resources and discussion on a collaborative electronic mailist list/resource sharing service. A key feature of the collaborative was the use of the CMQCC Maternal Data Center for data collection of structure, process, and outcome measures.	Uptake: Overall, 54% of hospitals completed 14 of 17 bundle elements, 76% reported regular unit-based drills, and 65% reported regular posthemorrhage debriefs. Feasibility: NR Providers: NR Procedures: NR Patients: Women in hospitals engaged in the hemorrhage CMQCC CPMS experienced a 21% reduction in severe maternal morbidity among hemorrhage patients over baseline. Not participating hospitals showed a nonsignificant 1.2% reduction over the same time period. Women with prior hemorrhage experience averaged a 29% reduction (without prior experience 15% reduction (both significant changes).
Fine 2014 [42] US Pain management	Long-term care facility # hospitals: 7 # providers: 51 # patients: 350 Pre-post	Toolkit: Enhancing the Management of Neuropathic Pain in the Long-term Care Setting Implementation: Algorithm and instructions for implementation, sample data collection form, performance measure instructions, slides, list of resources Provider: Assessment videos, clinical guidelines, assessment scales, pocket guide Patient: NR Other intervention: In-service meeting led by an expert faculty member	Uptake: NR Utility: NR Providers: NR Procedures: Patients with documented pain care plan increased from 86 to 93%; documented pain assessments on admission, a physical exam to assess pain, documented causes of pain symptoms, and assessment of effectiveness of pain management by a doctor did not change significantly Patients: NR
Pulver 2012 [12] Australia/New Zealand Pain management	Hospital # hospitals: 73 # providers: NR # patients: 716 Pre-post	Acute Postoperative Pain Management (APOP) Toolkit Implementation: Slide deck, informational resources such as evidence-based information on key messages to structure interactions with hospital staff, further reading list, electronic drug use evaluation audit tools, posters Provider: Pain scales, pain assessment and pain management plan discharge checklist (can be laminated for use at ward stations and medication folders) Patient: Patient guide Other intervention: NR	Uptake: NR Utility: NR Providers: NR Procedures: Patients who had a pain score used to assess pain at rest and movement 48–52 vs previously 24% (*p* < .0001), patients with documented pain management plan 58–65 vs 40% (*p* < .0001) Patients: NR
Parkman 2013 [32] US Patient safety	Hospital # hospitals: 4 # providers: 25 out of 87 initially interested # patients: NR CT	Professional Conduct Toolkit Implementation: Online educational modules focusing on teamwork, communication, management support, and reporting of errors (slides, videos), TeamSTEPPS action planning guide Provider: Tip sheets for connecting, conflict competence checklist Patient: NR Other intervention: NR	Uptake: NR Utility: 50% of respondents found the information “some” or “very much” helpful, 95% stated that their expectations for the course were met or exceeded Providers: No difference in safety perception between groups, no increased culture of safety awareness (knowledge), sensitivity (attitude), and competence behaviors (skills and actions); communication openness mean scores were unchanged at time 1 and 2 Procedures: NR Patients: NR
Schauberger 2006 [25] US Patient safety	Ambulatory care clinics # hospitals: 7 # providers: NR # patients: NR Pre-post	Ambulatory Patient Safety Toolkit Implementation: Description of 11 evidence-based practices, descriptions of methods to accomplish goals, forms, worksheets, tools, criteria for accomplishment, suggestions for reassessment Provider: NR Patient: NR Other intervention: Half-day videoconference, collaborative meeting before improvement phase	Uptake: All teams implemented best practices in all 11 categories Utility: Patient partnering, allergy list accuracy, and medication list accuracy were perceived as the most difficult and longest to implement Providers: NR Procedures: Medication list accuracy improved from 61 to 75% (*p* < .001), sustained at 1 year (76%); allergy list accuracy was 89% at baseline and did not change significantly (87%, 90%) Patients: NR
Thomason 2016 [88] US Patient Safety	Veterans Health Administration Spinal Cord Injury/Disorder (SCI/D) Centers # hospitals: 23 # providers: 51 # patients: NR Pre-post	Spinal Cord Industry Pressure Ulcer Monitoring Tool (SCI-PUMT) Toolkit Implementation: Flyer, video-recorded presentations, knowledge and competency verification tests, two manikins, facility implementation plan, guidelines for overcoming barriers Provider: SCI-PUMT, quick reference guidelines, healing continuum and graph, pocket guide Patient: NR Other intervention: Introduced at a conference, implementation supported by learning collaborative’s clinical champions and networking calls.	Uptake: 3254 downloads of SCI-PUMT toolkit items. The average number of potential SCI-PUMT users per SCI/D center was 11 (most report 1-3 certified wound care specialists available. 65% reported that SCI-PUMT training resulted in > 50% of potential users at their sites. 30% of sites were classified as high adopters (76-100% of staff using the SCI-PUMT); 52% reported < 50% of PrUs were assessed using the SCI-PUMT. Three sites used all 15 toolkit elements. Feasibility: 24 facilitators and 38 barriers were identified. Most barriers to implementation reflected “contextual” factors (time constraints, availability of the certified wound care nurses and documentation, training, leadership issues). Documentation was viewed as both a facilitator and barrier. One-third of clinical champions identified their ability to document the SCI-PUMT as a facilitator to implementation, whereas many sites identified the inability to document SCI-PUMT elements in the VA electronic healthcare record as a major barrier to implementation. Other facility-reported barriers included challenges in educating staff, the time required for SCI-PUMT completion, and staff buy-in. Providers: Response rate for the knowledge verification of conference participants was high: pretest 94 and posttest 84%. Only 3 of the 10 knowledge-based questions were answered correctly by > 85% of participants on the pre-test; all 10 questions were answered correctly by at least 95% of participants on the post-test. Procedures: NR Patients: NR
Lannon 2008 [24] US Pediatric preventive care	Primary care # hospitals: 15 # providers: NR # patients: NR Pre-post	Bright Futures Training Intervention Project toolkit Implementation: Planning guides, progress reports, inventory tools, planning worksheets, slide presentations, Plan-Do-Study-Act cycle tool, user guides to tools Provider: Screening tools, care algorithms, visit documentation forms Patient: Handouts Other intervention: 2 workshops, training in quality-improvement methods, monthly conference calls and data feedback, and listserv moderated by faculty	Uptake: Tested or implemented: recall/reminder systems (87%), community linkages (80%), and identification of children with special health care needs (80%); no practice implemented all 6 components; 9 practices tested or implemented 1 or 2 components Utility: Practice teams reported that facilitators of office system implementation included the perception that a component could be implemented quickly and/or easily, especially when a tool or template was immediately available. Barriers included time, cost, lack of agreement with recommendations, and lack of belief that changes would lead to improved health outcomes Providers: NR Procedures: Increases in the use of a preventive services prompting systems and the proportion of families asked about special health care needs (*p* < .0001); proportion of children who received a structured developmental assessment and use of strength-based approaches were not statistically significant Patients: NR
Byrne 2011 [36] US Perinatal care	Hospital # hospitals: 1 # providers: NR # patients: 27,737 births, 60,015 triage evaluations Pre-post	California Perinatal Quality Care Collaborative Antenatal Corticosteroid Therapy (ANS) Toolkit Implementation: Evidence-based guidelines, user’s guide, rationale, examples of hospital based quality improvement tools, problem identification worksheets Provider: NR Patient: NR Other intervention: Part of quality improvement initiative	Uptake: NR Utility: NR Providers: NR Procedures: Performance on quality measure for antenatal steroid administration increased from 77% to 100% (*p* < .01), significantly higher than the mean state-wide performance (*p* < .01) Patients: NR
Kohler 2015 [71] US Perinatal care	Hospital # hospitals: NR # providers: NR # patients: NR Pre-post	Improving Health Care Response to Preeclampsia Implementation: Patient care and treatment recommendations, simulations/drills, sample nursing management policy and procedure, classification of evidence grading, slideset for professional education, etc. Provider: Suspected preeclampsia and eclampsia algorithms, preeclampsia early recognition tool, sample medication boxes, ICD-9 coding, etc. Patient: Patient information (information, sheet, sample discharge instructions) Other intervention: Participation in the California Maternal Quality Care Collaborative Preeclampsia Collaborative	Uptake: NR Feasibility: NR Providers: NR Procedures: At baseline 14% of women with blood pressures meeting the criteria were treated, post-intervention 84% were treated within 30 minutes and 90% within 60 min. Patients: NR
Lyndon 2016 [74] US Perinatal care	Hospital # hospitals: 31 # providers: 22 # patients: NR Post-only	Improving Health Care Response to Obstetric Hemorrhage Version 2.0 A California Quality Improvement Toolkit ("Obstetric Hemorrhage Toolkit") Implementation: Emergency management plans (checklist, table chart, flow chart, pocket card), carts/kits/trays checklist, educational tools, educational information, professional education slideset Provider: Poster, debriefing tool, massive transfusion event protocols, blood loss calculator and quantification tools, etc. Patient: Patient education resources Other intervention: Part of year long quality care collaborative with phone calls	Uptake: NR Feasibility: Common issues.that served to either help or hinder implementation related to organizational context, including local culture within the organization; local structure and experience of the implementation team; degree of administrative support there was for the team and the project in terms of resources for equipment, personnel, and data collection; existing resources already in place in a given institution; clinician engagement that was affected by relationships between different departments; quality of communication; and degree of hierarchy in existing relationships. Most respondents rated the 10 components as “very useful—critical to retain.” Two recommended practices were rated lower in both degree of implementation and usefulness: routine active management of the third stage of labor, and hemorrhage debriefings. Each of these two recommended practices was rated as critical to retain by 60% of participants. Providers: NR Procedures: 77% indicated they had implemented or implemented and sustained each of the 10 recommended practices. Patients: NR
Ezzat 2017 [62] Canada Physical therapy	Practices # hospitals: NR # providers: 238 # patients: NR Post-only	Achilles tendinopathy Toolkit (ATT Implementation: Summary of the evidence of physical therapy interventions with clinical implications, details (e.g., methods and findings) of the individual articles that informed the evidence summaries Provider: Treatment algorithm, relevant outcome measures and supporting resources specific to: exercise prescription, LASER dosage calculation, major medical and surgical interventions Patient: NR Other intervention: To support its adoption by PABC members, a webinar demonstrating how to use the ATT involving a series of case studies was conducted in June 2012 with over 100 participating PTs and subsequently over 800 views of the recorded session. It has also been presented at national and international conferences.	Uptake: Regarding toolkit awareness, 81% said they were aware of the ATT, the majority learned about the ATT through PABC email/website (95%). Of those who indicated they were aware of the ATT, 53% indicated that they had explored its contents. Feasibility: 86% agreed that the toolkit was helpful in clinical decision-making. The toolkit assisted in informing treatment and exercise progressions, provided confirmation and confidence of treatment approach and acted as a guide with evidence to inform clinical decision-making. 88% would recommend the toolkit to others. Time was the most frequently indicated barrier (45%) followed by: “the quality of some of the evidence in the toolkit is questionable” (23%); “the toolkit provides ‘recipes’ that do not allow for enough decision making by the therapist” (15%) and “I don’t know where to find or access the toolkit” (15%). 31% indicated no barriers. When asked if anything was missing from the ATT, participants suggested some additional treatment strategies, as well as requesting patient handouts or pictures and a more concise summary of the research. Providers: 49% indicated that they had changed their clinical practice based on the knowledge gained from the ATT, 87% agreed that they feel more justified in applying physiotherapy treatments that they were already using. 9% indicated they were very aware of the evidence before exploring the ATT, this increased to 45.5% of respondents after exploring the ATT. 46% agreed that they were better equipped to collaborate with physicians or other health care professionals after exploring the ATT. Procedures: 18% met the full combined criteria for following the toolkit recommendations for treating AT; use of outcome measures 51%; those who explored the ATT had greater odds of following the best practice recommendations for each of the individual questions and all criteria combined (OR 2.8; CI 1.3–6.0). Patients: NR
Brown 2015 [57] US Psychotherapy decision support	Psychotherapy clinic # hospitals: NR # providers: 704 # patients: 9,785 (in first 18 months), 30,410 (after >18 months) Pre-post	A Collaborative Outcomes Resource Network (ACORN) Clinical Decision Support Toolkit Implementation: NR Provider: Database of psychotherapy treatment outcomes, clinical information system, secure web interface Patient: NR Other intervention: None	Uptake: NR Feasibility: NR Providers: NR Procedures: NR Patients: The overall upward trend in psychotherapy outcomes is evident for all users (*d* 0.80 vs 0.87; 9% gain), but particularly among high frequency Toolkit users. Multiple regression with number of months and toolkit use as predictors of severity adjusted effect size revealed that both are separate contributors to the observed gains in effect size (*p* < .00, less than 0.1% variance overlap).
Sopcak 2016 [86] Canada Screening	Primary care # hospitals: 3 # providers: 25 # patients: 91 Post-only	Building on Existing Tools to Improve Chronic Disease Prevention and Screening in Primary Care (BETTER) Implementation: Clinical resources, clinical guidelines, etc. Provider: Spaghetti diagram, BETTER algorithms, BETTER health survey, bubble diagram, prescription, goal sheet, decision support tools, questionnaires, etc. Patient: Life expectancy calculator, risk assessments, action plans, guidelines, patient education information, smoking cessation plan, physical activity videos/toolkit/guide, etc. Other intervention: Before implementation, PPs participated in training provided by the BETTER team, which involved an introduction to the BETTER approach and tools, the prevention visit process and Brief Action Planning.	Uptake: NR Feasibility: Based on respondents’ answers, complexity and cost affected the implementation. In addition to completing forms for the visit, PPs (prevention practitioners) described that they had to complete data collection forms to enable the measurement of study outcomes. Primary care providers (physicians and PPs) identified the complexity of the intervention, particularly the amount of paperwork and the time needed to collect information, as the main barriers of the intervention. A 30–60-min prevention appointment would be too costly. The perceived fit of the intervention within primary care settings varied, some physicians were skeptical. Physicians in Newfoundland and Labrador did not have a billing code for prevention and lifestyle counseling at the time of the study, which was a barrier for implementing a CDPS program. In the context of competing health care demands and scarce resources, respondents expressed that acute care trumps prevention, limited resources, specifically the lack of staff, made it difficult to allocate more time or resources to CDPS. BETTER 2 was also perceived as not being a good fit with primary care providers who believed that they did a good job of CDPS already. The importance of having a local champion to facilitate program implementation was exemplified when one of the local champions left, recruitment and uptake stalled. Two activities emerged as essential for the implementation of BETTER 2: planning and engaging, and interprofessional collaboration. Providers: Data suggested that to be successful in their role, PPs need skills in time management, planning for the PP visit, and prioritizing the medical and clinical information. The PPs also needed to be good communicators and effective listeners, who are comfortable with conducting personalized one-on-one visits with patients. Procedures: NR Patients: Patients commented positively on their prevention visits. Patients appreciated that the visits were personalized as well as the time taken to go over CDPS in a comprehensive way. Patients saw visits as beneficial for a variety of reasons. None expressed that the prevention visit with a PP was a duplication of services or that the information given at the visit was irrelevant. Patients felt that having CDPS visits with the PP helped alleviate the stress on physicians.
Shellhaas 2016 [85] US Screening, quality improvement	Obstetric practices # hospitals: 15 recruited, 12 completed # providers: 17 # patients: 700 (data), 83 (survey) Pre-post	Gestational Diabetes Mellitus (GDM) Toolkit Implementation: None Provider: Three-pocket folder with worksheets and tools for office flow and postpartum care, resources for GDM management, and general prenatal resources Patient: Two separate single-ring bound 5 × 7 in booklets: one for pregnant women at high risk of GDM, one for pregnant women diagnosed with GDM Other intervention: Monthly learning sessions (1-h webinars), four optional individual coaching calls	Uptake: 12/15 practices remained actively engaged during project period. > 70 provider toolkits, 2345 patient toolkits (845 high-risk and 150 diagnosed) disseminated Feasibility: The 3 sites that disengaged reported doing so for inability to commit time (3–5 h/month) and resources. 92% responded that provider toolkit resources were helpful or very helpful. All but one site reported that patient toolkit resources were helpful. Providers: NR Procedures: At project end, 59% of charts indicated a follow-up appointment scheduled within 4 weeks of GDM diagnosis. Of women with a delivery recorded, 69% completed a postpartum visit and 40% had a documented type 2 diabetes screen. Prenatal nutrition, weight gain, breastfeeding, and exercise education were over 90% at baseline. Baseline to 11 month follow up education rates were 67% to 100% for type 2 diabetes risk, 63% to 96% for family planning use, and 40% to 90% for smoking cessation. GDM screening prior to 28 weeks gestation was 87% at baseline and 95% at completion. Patients: 91% reported that resources provided were helpful or very helpful. 99% indicated they would attend their postpartum visit and 87% would probably or definitely be screened for type 2 diabetes.
Sarna 2017 [83] US Smoking cessation	Hospital # hospitals: 8 # providers: 283 # patients: NR Pre-post	Registered Nurses Referral to Quitlines - Helping Smokers Quit Louisiana Toolkit Implementation: 45-min prerecorded webcast and additional downloadable print resources on tobacco dependence treatment, website with national and state-specific tobacco control resources Provider: Clinician pocket guide, brochure, quitline card Patient: None Other intervention: None	Uptake: There are some significant differences in the tobacco dependence intervention outcomes of nurses who viewed the webcast, compared with those who did not (N unclear). Feasibility: NR Providers: NR Procedures: Changes in the proportion of nurses who consistently delivered the 5As significantly increased for all aspects of the intervention 3 months after the intervention, except Asking patients about their smoking status (high level at baseline), at 6 months, most changes were sustained. Nurses were more likely to Advise smokers to quit (OR 1.99, CI 1.23, 3.23, *p* < 0.005), and more likely to Assess willingness to quit, Assist with a quit plan, and to recommend the quitline (OR 4.38, CI 2.73, 7.03, *p* < 0.0001). Assisting with a quit plan and Arranging for follow-up were not sustained at 6 months. Consistent recommendation of a smoke-free home reached statistical significance at 6 months. Patients: NR
Shershneva 2010 [22] US Smoking cessation	NR # hospitals: NR # providers: 37 # patients: NR Post-only	CS2day Toolkit Implementation: Slide decks, patient cases, links to online resources Provider: Care algorithms, medication charts, drug interaction table, role-modeling videos, quit-smoking plans, posters for practice rooms Patient: Handouts in English and Spanish Other intervention: NR	Uptake: NR Utility: Survey results indicated that expectations were met and the tools were well-received Providers: Clinicians reported using tools as education materials for themselves, reminders in practice, patient handouts, resources for teaching others, and references to support system changes Procedures: NR Patients: NR
Pratt 2012 [15] US Staff trauma support	NR # hospitals: NR # providers: 36 # patients: NR Post-only	Clinician Support Tool Kit for Healthcare Implementation: 10 informational modules with tools, references, and examples; organizational assessment tool Provider: NR Patient: NR Other intervention: NR	Uptake: 725 of requested a download (of 6261 people who visited the website within 12 months) Utility: 75% of survey respondents found the toolkit extremely or very helpful, 88.9% found it easy to navigate, and 96.2% liked the format Providers: 62.5% of respondents reportedly used the toolkit to make positive changes in their institution Procedures: NR Patients: NR
Brooks 2013 [50] US Substance abuse	Outpatient substance abuse treatment center # hospitals: 3 # providers: 19 # patients: NR Pre-post	RoadMAP Relapse Prevention Group Counseling Toolkit Implementation: NR Provider: Handbook, clinician's guide to sessions, video vignettes, posters for practice room, worksheets, teaching aids Patient: Worksheets, recovery cards to document progress Other intervention: 3-h training	Uptake: 80% reported they voluntarily used toolkit materials during the time between the post-training and 6-month follow-up Utility: NR Providers: Moderate or large baseline to post-training effect sizes for counselor adherence to toolkit content for 13 of 21 targeted behaviors. Post-training adherence gains were largely maintained at the 6-month follow-up (non sign). Procedures: NR Patients: NR
Levy 2017 [73] US Substance use screening	Primary care # hospitals: NR # providers: 599 # patients: NR Pre-post	Adolescent Screening, Brief Intervention, and Referral for Treatment for Alcohol and Other Drug Use Toolkit for Providers Implementation: Educational materials, confidentiality laws, practice vignette, practice cases for role play Provider: Screening tools, referral resources, naloxone information, treatment program information, decision tree, quick guide Patient: None Other intervention: Distributed toolkit to all physicians in Massachusetts; also made available primary prevention print resources, as well as websites and phone consultation for advice and/or treatment referral information	Uptake: NR Feasibility: Regarding barriers to screening, 2014 respondents were less likely than 2008 respondents to endorse responses related to “lack of knowledge” (36 vs 52%, adjusted odds ratio [AOR] 0.5, *p* = 0.001). Lack of time or staff resources as barriers to screening remained unchanged. Providers: Logistic regression analyses controlling for the demographic characteristics (physicians’ practice setting and gender) found significantly higher rates of physicians in 2014 compared with 2008 reporting annual substance use screening (96 vs 88%, AOR 2.8, *p* = 0.04) and any use of a validated tool (56 vs 43%, AOR 1.8, *p* = 0.006). Procedures: NR Patients: NR
Nace 2011 [20] US Vaccination	Long-term care facility # hospitals: 6 # providers: NR # patients: NR Pre-post	American Medical Directors Association Immunization Toolkit Implementation: Booklet with educational material and resources for increasing the uptake of vaccinations and managing influenza outbreaks, 2 DVDs Provider: NR Patient: NR Other intervention: Email distribution list, half-day of collaborative training offered to 3/6 facilities	Uptake: NR Utility: NR Providers: NR Procedures: Facilities that received the toolkit alone experienced decreases in worker influenza (29.3% to 25.8%), resident influenza (78.9% to 58.2%), and resident pneumococcal (20.2% to 6.3%) immunization rates. Facilities who received the tooklkit and collaborative training increased rates by 10.9, 4, and 29.9 percentage points. Patients: NR
Nowalk 2014 [51]; Nowalk 2013 [51]; Nowalk 2012 [96] US Vaccination	Primary care # hospitals: 4 # providers: 24 # patients: 5592 Pre-post	4 Pillars Toolkit Implementation: Educational materials, case studies, lists of barriers, supplementary ideas, bibliography, organized in 4 pillars (convenient access, notification, office system changes such as immunizations given as part of vital signs, motivation elements such as feedb Provider: Standing order program, prompts in electronic medical record Patient: Leaflet, posters Other intervention: Introduction to toolkit in lunch meeting, weekly progress reports to immunization champion, online refresher course ($20 incentive)	Uptake: All sites reported using at least 5/14 strategies Utility: Most staff at 3/4 sites believed the toolkit improved efficiency for adult vaccinations Providers: NR Procedures: Pneumococcal vaccination rates increased for high-risk adults (25 vs 40%, *p* = .02) but not for older adults (44% vs 52%, *p* = .26) and in 2/4 practices among both high-risk and older adults (p<.05); influenza vaccination rates increased significantly in 3/4 sites and overall (22 vs 33%, *p* < .001) Patients: NR
Zimmerman 2014 [46]; Lin 2016 [111]; Nowalk 2014 [54]; Nowalk 2016 [53] US Vaccination	Primary care # hospitals: 20 # providers: NR # patients: 536-8183 patients/facility RCT	4 Pillars Toolkit for Increasing Childhood Influenza Immunization Implementation: see Nowalk et al. (2013) Provider: see Nowalk et al. (2013) Patient: see Nowalk et al. (2013) Other intervention: EMR programmed to include best practice alert, visited to introduce the toolkit at a meeting, provided feedback on immunization rates, created online videos and a PSA for local TV, community outreach efforts, early delivery of vaccine, donated vaccines	Uptake: All intervention sites used 1 or more elements of the toolkit Utility: NR Providers: NR Procedures: Intervention practices increased vaccination rates more than controls (*p* = 0.034) Patients: NR
Abraham 2007 [39] US Weight management	Primary care # hospitals: 5 # providers: 183 # patients: NR RCT	America-on-the-Move Toolkit Implementation: Tip sheets, posters Provider: BMI charts Patient: Patient readiness assessment, educational patient materials Other intervention: NR	Uptake: NR Utility: 64% of providers felt behavioral counseling training would improve toolkit effectiveness Providers: NR Procedures: Control providers provided nutrition counseling to overweight patients in 40–49% of visits, compared to 30-39% among intervention providers Patients: NR
Gibson 2016 [64] US Weight management	Primary care # hospitals: 2 # providers: NR # patients: 134 Pre-post	5210 Let's Go! Childhood Obesity Resource Toolkit for Healthcare Professionals Implementation: Educational materials and references, toolkit user guide, etc. Provider: Healthy Habits Questionnaire, BMI-for-Age Growth Charts, decision support chart, motivational interviewing materials, etc. Patient: “5210” posters and drink comparison displays, educational brochures, etc. Other intervention: Trackers for chosen; staff education: (a) accuracy with anthropometric measures to facilitate correct diagnosis of overweight and obesity, (b) assessment and evaluation of the child’s lifestyle behaviors through use of a questionnaire, (c) consistent health messaging related to nutrition and physical activity, and (d) use of motivational interviewing to guide a mutually established action plan.	Uptake: Each clinic implemented the “5210” program with all child office encounters and not just for wellness visits. Feasibility: An unexpected finding was the importance of establishing incentives and a reward system for “5210” participants. Providers: NR Procedures: Profound changes occurred with large shifts in documentation of BMI percentile (from 27 to 98%; *p* < 0.05), education and counseling (from 9 to 87%; *p* < 0.05), and accurate diagnosis of overweight or obesity (from 0 to 32%; *p* < 0.05). There was a statistically significant decrease in documentation of blood pressure readings (from 72 to 60%; *p* < 0.05). Use of the screening questionnaire increased from 0 (was not utilized before the project) to 88%. Patients: The education foci that were prioritized and selected by 89% included eat more fruits and vegetables (35%), spend less time watching television and playing video/computer games (25%), and drink more water (21%) and less sugar-based beverages (8%). Parents, especially of younger children, commented that the questionnaire heightened the awareness of the lifestyle habits of the family and motivated the parent to make changes in their diet and physical activity.
Kinsinger 2009 [37] US Weight management	Primary care # hospitals: 17 # providers: NR # patients: 30–70 patients/facility Post-only	MOVE (Managing Overweight/Obesity for Veterans Everywhere) Weight Management Program Implementation: Administrative manuals, posters, banners, pens Provider: Promotional brochures, clinical references, BMI chart, online training modules Patient: Handouts Other intervention: Biannual screening for overweight/obesity, online baseline assessment and summary report, diet/activity logs, pedometers (optional), telephone check-ins, group support sessions (optional)	Uptake: 98.7% of VA facilities have MOVE! programs in place. 3000–4000 patients per month have a first move visit. 100,000 patients have had at least 1 Move!-related visit. Of patients who might benefit, 7.5% have participated Utility: NR Providers: NR Procedures: 66% of primary care patients have been screened for obesity Patients: NR
Rueda-Clausen 2014 [82] Canada Weight management	Primary care # hospitals: 4 # providers: NR # patients: 102 Pre-post	5As of Obesity Management for Adults Implementation: Evidence for use, core principles Provider: 5As (adult, pediatric, pregnancy) practitioner checklist, desktop tool to facilitate discussion, practitioner guide Patient: Patient tear-off pad Other intervention: 90-min standardized training program	Uptake: NR Feasibility: NR Providers: NR Procedures: At baseline, 19% of patients with obesity reported that their provider initiated a conversation about their weight. Implementing the 5As tool and training did not affect the frequency of standard practices such as measuring blood pressure, body weight or waist circumference, but it did cause an increase (19–39%, *p* = 0.03) in the number of participants reporting a dialogue about weight management. The tool also improved the follow-up/and coordination activities and increased the use of all the obesity management 5As components (significant for both Assess and the Assist components). Patients: NR
Sample 2013 [35] US Weight management	Primary care (pediatric) # hospitals: 4 # providers: 24 out of 56 invited # patients: NR Post-only	Pediatric Obesity Toolkit Implementation: Educational materials and tools, child obesity rates by county map of North Carolina Provider: Clinician reference guide, BMI and blood pressure charts, decision support charts, reference lists, motivational interviewing guide, community resources, algorithms, prescription pads Patient: Handouts Other intervention: 1-hour training session	Uptake: Universal assessment and screening tools and patient handouts were used on a daily basis by most respondents; 1/4 reported using the staged treatment, motivational interviewing guide, and quick reference guides weekly; 33% indicated that the ICD-9 codes, Utility: 29% of respondents cited ICD-9 codes and reference articles as the most useful tools; 64% rated ICD-9 codes as very useful and 57% found the reference articles very useful Providers: NR Procedures: 39% stated that they counseled every child and family on weight and healthy lifestyles, 62% counseled only during well-child checks, no providers counseled about weight and healthy lifestyles during sick child visits Patients: NR
Smith 2011 [21] US Weight management	Primary care # hospitals: NR # providers: 226–278 responded out of 1429–1580 invited # patients: NR Pre-post	Americans In Motion (AIM) to Change Toolkit Implementation: Practice manual to develop a culture of fitness, implementing small changes in the practice Provider: BMI calculator, presciption pads Patient: Posters, food and activity journal, fitness assessment Other intervention: NR	Uptake: 46% used the BMI calculator, 10% the fitness prescription pads for at least some patients, 46–48% did not use any toolkit components Utility: 21–24% reported systematic screening was not feasible due to time constraints Providers: Attitudes about the importance and usefulness of BMI increased during the study Procedures: Rates of height measurement (57% to 74%) and BMI calculation (50% to 70%) increased; 19% of providers used the food and activity journal, 11% the personal fitness assessment Patients: NR
Stiff 2014 [21] US Weight management	Primary care # hospitals: 1 # providers: 22 # patients: 88 Pre-post	Promoting Healthier Weight in Adult Primary Care Implementation: Assessment for readiness to change, literature overview and references, implementation tips Provider: Clinical algorithm, motivational interviewing techniques, online resources, charts and definitions (“what is a serving”) Patient: Weight and health profile form, patient informational resources Other intervention: Clinic staff training and support, education packet specific for patient’s stage of change	Uptake: NR Utility: Estimated time spent using the toolkit: < 3 min (23%), 3–7 min (54%), 8–11 min (23%), > 12 min (23%); 85% of providers stated that the extra time was beneficial for the patient, 8% indicated the toolkit took too much time; Over half of providers reported they liked using the tool and thought it should continue to be used in the clinic Providers: 70% thought nursing staff should use the toolkit and refer for follow-up as appropriate Procedures: NR Patients: The average acceptability rate of the toolkit among patients was over 90%
Mulloy 2008 [33] US Wrong site surgery	Hospital # hospitals: 325 # providers: 519 # patients: NR Pre-post	AORN Correct Site Surgery Tool Kit Implementation: Guidelines for implementing universal protocol for Wrong Site Surgery, letters to nurses, physicians, facility chief executive officers, and healthcare risk managers; FAQs Provider: Pocket reference card, educational program on CD, policy template Patient: Information for patients Other intervention: NR	Uptake: NR Utility: 92% of registered nurses and 73% of the hospital respondents found the Correct Site Surgery Tool Kit helpful; 68% of registered nurses stated they had changed their practice after receiving the toolkit Providers: NR Procedures: NR Patients: The reported rate of wrong site surgery per 100,000 surgeries peaked initially (4.27), with declining rates in one (3.67) and 2 years later (3.14)

*CI* 95% confidence interval, *BMI* body mass index, *EMR* electronic medical record, *ICD* international classification of diseases, *MD* mean difference, *N* number of, *NR* not reported, *OR* odds ratio

Toolkit categories: implementation aiming to introduce the intervention, initial awareness campaign; provider: elements supporting healthcare providers during routine care; Patient: material for patients such as handouts;

Result categories: uptake: uptake of toolkit or toolkit components and adherence to toolkit/components; utility: information on the feasibility of using the toolkit, acceptability of the toolkit and its components, reported barriers and facilitators, and staff satisfaction with the toolkit; provider: effects on learning, self-reported confidence, or attitudes, self-reported behavior changes, and intentions; procedures: changes in procedures, organizational results (e.g., tests ordered, costs); patients: patient health outcomes, patient satisfaction, and other patient-reported outcomes

^a^The evidence table is organized by toolkit topic

#### Quality assessment

As a critical appraisal tool, the QI-MQCS targets the informational quality of QI studies and informs decisions about applicability of results to other settings. The number of criteria met per study ranged from 3 to 14 (mean 9.78, SD 3.04). Since the objective of this systematic review was to assess the spread of QI interventions through the use of toolkits, 100% of included publications/studies addressed *Spread* and described the ability of the intervention to be replicated in other settings.

In addition, for ten of the 16 domains, more than 50% of the included publications met the minimum QI-MQCS criteria. The top five described aspects related to study initiation and included *Organization motivation* (description of the organization reason, problem, or motivation for the intervention, 93%); *Intervention rationale* (description of the rationale linking the intervention to the effects, 88%); *Intervention* (description of the processes, strategies, content, and means of achieving the effects associated with the intervention and considered to be permanent as opposed to activities considered to be temporary for the purpose of introducing the intervention, 70%); *Implementation* (description of the approach to designing and/or introducing the intervention, 81%); and *Data sources* (documentation of how data were obtained and whether the primary outcome was defined, 82%). The other five domains, for which more than 50% of studies met minimum QI-MQCS criteria, included *Organizational characteristics* (description of setting demographics and basic characteristics, 68%); *Timing* (clear outline of the timeline for intervention implementation and evaluation so that follow-up time can be assessed, 60%); *Adherence/fidelity* (level of compliance with the intervention, 57%); *Organizational readiness* (description of QI culture and resources available for the intervention, 64%); and *Limitations* (outline of limitations and the quality of the interpretation of findings, 68%).

The five domains, for which less than 50% of studies met minimum QI-MQCS criteria, addressed evaluation of results and included *Study design* (documentation of the evaluation approach with respect to study design, 36%); *Comparator* (description of the control condition against which the intervention was evaluated, 26%); *Health outcomes* (inclusion of patient health outcomes in the evaluation, 17%); *Penetration*/*reach* (reporting of the proportion of eligible units that participated in the intervention, 29%); and *Sustainability* (information on the potential for maintaining or sustaining the intervention with or without additional resources, 40%).

### Key question 1: what are common elements of quality improvement toolkits?

The evaluated toolkits addressed a variety of quality improvement approaches. Most focused on a specific clinical topic rather than general healthcare provider behaviors. Seven toolkits addressed weight management; four toolkits evaluated in five studies addressed fall prevention; three, emergency preparedness; three each patient safety and three perinatal care; and two (evaluated in three studies) were aimed at vaccination. We identified two toolkits each addressing the topics asthma management, cancer screening, elective delivery, health literacy, hospital-acquired infections, hospital readmission, medical errors, mental health, pain management, screening, smoking cessation, and substance use. The other toolkits addressed antimicrobial stewardship, autism communication, brain injury symptom management, cancer care, cardiac care, care quality, clinical decision making for critical care, depression care, diabetes care, end of life care, geriatric care, heart failure, hepatitis C care, kidney disease care, medication management, multiple sclerosis symptom management, newborn screening, nursing best practices, obstetric care, parental education, pediatric preventive care, psychotherapy decision support, staff trauma support, and wrong site surgery.

The toolkits varied in length and complexity and included a large variety of elements. Most toolkits were downloadable online and free of charge. The toolkit format was often a consolidated text document with written material. Some toolkits used a website with downloadable individual tools and links to additional online resources. Some toolkits included other material such as alcohol hand rubs or peak flow meters, in branded packages, and eight toolkits included a software program. Table 1 includes the toolkit components; further details, including the link to a downloadable copy of the toolkit, can be found in Additional file 2.

#### Implementation toolkit elements

As the summary Table 2 documents, the majority of the 72 toolkits evaluated in 77 studies included material designed to help with the introduction and implementation of the specific intervention promoted in the toolkit. This typically included educational material such as research summaries, supporting evidence for healthcare interventions, and further reading lists. Some toolkits included downloadable slide decks for presentations to staff, links to online videos to introduce the clinical issue or the intervention, information on achieving change in organizations such as action plan templates, institutional self-assessment tools, templates to collect performance data to facilitate audits and research, templates or actual material to raise awareness such as posters, and many included practical “implementation tips.” As the evidence table shows, many toolkits included unique additional practical tools such as letters to management staff to raise awareness; briefing notes; detailed material for training courses (e.g., daily timetable or teach-back technique) to facilitate staff education; and other tools useful for staff such as a list of frequently asked questions, cost calculators, worksheets, or example forms.

**Table 2:** Toolkit element and data summary

Study ID Topic	Implementation toolkit element	Provider toolkit element	Patient toolkit element	Other intervention	Uptake data	Feasibility data	Procedure data	Provider data	Patient data
Ashiru-Oredope 2016 Antimicrobial stewardship (AMS)	x	x			x		x		
Jones 2017 Antimicrobial stewardship (AMS)	NA	NA	NA	x	x	x	x	x	
Bender 2011 Asthma management	x	x	x	x	x		x		
Taylor 2017 Asthma management	x	x	x	x					x
Nicolaidis 2016 Autism communication	x	x	x	x		x			x
Chrisman 2011 Brain injury symptom management	x	x	x					x	
Latsko 2015 Cancer care	x	x	x		x			x	
Gulati 2015 Cancer screening		x		x	x	x	x	x	
Spruce 2012 Cancer screening	x	x	x			x		x	
Adsett 2014 Cardiac care	x	x	x			x			
Callard 2012 Care quality	x		x		x				
Kemertzis 2018 Clinical decision making	x	x	x	x	x	x	x	x	
Pierce 2016 Critical care	x				x	x	x		
Han 2013 Depression care	x	x	x		x	x		x	
Fowles 2014 Diabetes care	x	x	x	x				x	x
Gray 2017 Diabetes care	NA	NA	NA	x		x	x	x	
Alidina 2015 Elective delivery	x	x	x		x		x		x
Chesis 2015 Elective delivery	NA	NA	NA	x			x		
Clancy 2012 Emergency preparedness	x	x	x		x	x	x		
Wyte-Lake 2016 Emergency preparedness	x	x		x		x			
Henry 2012 Emergency/surgery capacity	x	x		x	x		x		
Cox 2017 End-of-life care	x	x		x				x	x
Carroll 2012 Fall prevention		x	x				x		
Coe 2017 Fall prevention	x	x	x	x	x	x	x		
Dykes 2017 Fall prevention	NA	NA	NA		x	x	x		x
Fisher 2013 Fall prevention	x	x			x				x
Stalhandske 2008 Fall prevention	x	x	x	x			x		x
Ryan 2013 Geriatric care	x	x	x	x	x			x	
Dore 2013 Health literacy	x	x	x	x	x	x	x	x	
Mabachi 2016 Health literacy				x	x	x		x	
Koelling 2006 Heart failure	x	x	x	x					x
Perumalswami 2016 Hepatitis C care		x	x		x				
Adams 2014 Hospital readmission	x	x	x	x			x		x
Mitchell 20015 Hospital readmission	x	x	x	x	x	x	x	x	x
Enfield 2014 Hospital-acquired infections	x	x		x	x		x		x
Randle 2006 Hospital-acquired infections	x	x	x	x		x	x	x	x
Septimus 2016 Hospital-acquired infections	x	x		x		x			x
Haley 2015 Kidney disease care	x	x	x	x	x	x	x	x	
Fernald 2015 Medical errors	x			x	x	x		x	
Leape 2006 Medical errors	x	x		x	x	x			
Mueller 2013 Medication management	x	x		x		x		x	
McHugo 2007 Mental health	x	x		x	x		x		
MacDonald-Wilson 2017 Mental health decision support	x	x	x	x	x	x		x	
Miller 2014 Multiple sclerosis symptom management	x	x		x		x	x	x	
Guillory 2017 Newborn screening	x	x	x	x			x	x	x
Dobbins 2005 Nursing best practices	x			x	x	x			
Main 2017 Obstetric care	x	x	x	x	x				x
Fine 2014 Pain management	x	x		x			x		
Pulver 2012 Pain management	x	x	x				x		
Kuhlman 2014 Parental education		x	x				x		
Parkman 2013 Patient safety	x	x				x		x	
Schauberger 2006 Patient safety	x			x	x	x	x		
Thomason 2016 Patient Safety	x	x		x	x	x		x	
Lannon 2008 Pediatric preventive care	x	x	x	x	x	x	x		
Byrne 2011 Perinatal care	x			x			x		
Kohler 2015 Perinatal care	x	x	x	x			x		
Lyndon 2016 Perinatal care	x	x	x	x		x	x		
Ezzat 2017 Physical therapy	x	x		x	x	x	x	x	
Brown 2015 Psychotherapy decision support		x							x
Sopcak 2016 Screening	x	x	x	x		x		x	x
Shellhaas,2016 Screening, quality improvement		x	x	x	x	x	x	x	x
Sarna 2017 Smoking cessation	x	x			x		x		
Shershneva 2010 Smoking cessation	x	x	x			x		x	
Pratt 2012 Staff trauma support	x				x	x		x	
Brooks 2013 Substance abuse		x	x	x	x			x	
Levy 2017 Substance use screening	x	x		x		x		x	
Nace 2011 Vaccination	x			x			x		
Nowalk 2014 Vaccination	x	x	x	x	x	x	x		
Zimmerman 2014 Vaccination	NA	NA	NA	x	x		x		
Abraham 2007 Weight management	x	x	x			x	x		
Gibson 2016 Weight management	x	x	x	x	x	x	x		x
Kinsinger 2009 Weight management	x	x	x	x	x		x		
Rueda-Clausen 2014 Weight management	x	x	x	x			x		
Sample 2013 Weight management	x	x	x	x	x	x	x		
Smith 2011 Weight management	x	x	x		x	x	x	x	
Stiff 2014 Weight management	x	x	x	x		x		x	x
Mulloy 2008 Wrong site surgery	x	x	x			x			x
Frequences	89%^a^	88%^a^	63%^a^	69%^b^	55%^b^	56%^b^	57%^b^	40%^b^	29%^b^

Note: NA not applicable (to not count the toolkit twice), MS multiple sclerosis, NICU neonatal intensive care unit

^a^Out of 72 toolkits

^b^Out of 77 publications

#### Provider toolkit elements

Tools that targeted healthcare providers specifically were also included in most toolkits. Tools encompassed care plans, treatment and management algorithms, decision support, or clinical practice guidelines. In addition, many toolkits included assessment scales that providers could apply in clinical practice. Some toolkits also included pocket cards for clinicians, checklists to be used in clinical consultations, written scripts for healthcare providers, practice demonstration videos for providers to perform the intervention, and ready-to-use forms for patient care. A few toolkits included additional tools such as body mass index (BMI) calculators, spirometers, alcohol hand rubs, or prescription pads (see Table 1).

#### Patient toolkit elements

As the evidence and summary tables show, about two-thirds of toolkits included material for direct dissemination to patients. In the large majority, these were informational handouts or more comprehensive educational materials such as treatment brochures. Some toolkits included bilingual material and several contained posters and ward notices directed at patients. Other, less common resources directly targeting patients or caregivers included patient self-assessment tools, checklists (such as for appointments), activity journals and diaries, links to online resources for patients, educational videos, or peak flow meters for patients.

### Key question 2: what is the uptake and utility of published quality improvement toolkits?

A majority of included studies reported on the uptake and/or utility of the evaluated toolkit.

#### Uptake

Fifty-five percent of studies reported information on the uptake and use in practice of and the adherence to the toolkit or its components, but the type and informational value of reported data varied widely.

Several reported download statistics for online tools or requests for the toolkit [11, 15, 29–31, 67, 88, 90], but most studies reported no denominator and reported the total number of downloads at the time of the publication with no further detail. Three studies that reported a point of reference stated that 2000 toolkit copies were downloaded in 7 months [11], that 725 copies had been downloaded in 1 year [15], or that the toolkit had been accessed by 8163 practitioners over 255 days [67]. Some studies tracked which or how many individual tools included in the toolkit had been adopted by the end users [21, 24, 25, 29, 34, 35, 40, 46, 51, 56, 61, 64, 69, 75, 76, 78, 81, 88]. The evidence table shows variable uptake with no studies reporting full uptake of the toolkit. Uptake of components ranged from 10% (fitness prescription pads) [21] to 87% (recall/reminder system installed) [24].

Five studies documented staff awareness of the toolkit and whether the distributed toolkit had been reviewed by eligible users; the studies with numerical results reported high, but not perfect review rates (81–86%) [13, 29, 56, 62, 68]. Two studies reported on the proportion of eligible participating sites that adopted the toolkit; results ranged from 53 to 98% [14, 19]. Several studies reported on adoption of the intervention promoted in the toolkit: 98.7% of VA facilities have MOVE! programs in place [37], 10 to 15% of teams were unable to get beyond the planning stage and 50 to 65% implemented the medical error prevention practices partially or fully [27], 67% of provinces and 53% of hospitals implemented an emergency preparedness program [14], 7/10 sites successfully implemented a discharge program as planned [78], one indicated that all components of a protocol to prevent hospital-acquired infections had been implemented (but some had already been in place before the project) [40], one study reported that 54% of hospitals completed 14 of 17 intervention bundle elements [77], all teams had implemented best practices in all toolkit categories [25], one reported varying results across intervention components (e.g., 80% identification of children with special health care needs) [24], all sites reported using at least 5/14 strategies to increase vaccination rates [51], and one study indicated that each participating clinic implemented a specific weight management program strategy in all child office encounters and not just for wellness visits [64]. Individual studies reported the proportion of adopting hospitals out of those approached [19, 27, 30, 76], tracked the number of sites completing the toolkit evaluation project [38, 76, 85], surveyed how clinicians used the tools [22], or recorded which sites continued to use the toolkit after the implementation period, with or without substantial changes [10, 50].

#### Utility

Half of included studies reported on the utility, feasibility, or acceptability of, the satisfaction with, or the barriers to using the toolkit, its components, or the intervention promoted in the toolkit.

Reported satisfaction with the toolkit was generally high. One study reported that 50% of respondents found the toolkit information “some or very much helpful” [32], another reported 75% of respondents found the toolkit “extremely or very helpful” [15], one study reported ratings of “being helpful to staff” that ranged between 73 and 92% [33], one study documented that clinicians were “extremely satisfied or satisfied” in 11/11 discussions [70], in one study 86% of respondents agreed that the toolkit was helpful in clinical decision-making [62], and another study reported that 85% of staff who had read the toolkit found it helpful [29]. One study reported that most staff at three out of four sites believed the toolkit improved efficiency for adult vaccinations [51], one study found that all participants were “very satisfied or satisfied” with the overall usefulness of the toolkit [17], and one highlighted that the toolkit enabled comprehensive disease management and improved overall patient care [43]. In another study, most staff and stakeholders had described the toolkit as a useful resource [69], and three studies indicated that feedback was “positive” [22, 23, 63]. Two studies reported mixed feedback [67, 79]: while most providers found the toolkit moderately or very useful, several noted that they already were doing what was recommended [79]. One study found that the perceived helpfulness of the toolkit decreased over time after implementation of the intervention [89].

For feasibility, ten studies indicated that the interventions or best practices included in the toolkit were not feasible [13, 21, 25, 27, 34, 59, 73, 84–86]. For example, a quarter of participants in one study reported that systematic screening for obesity was not feasible in clinical practice [21]. Up to 91% of teams found implementing the recommended practices difficult in another study [27], and one study highlighted that 54% of users reported that incorporating health literacy techniques added time to the patient’s visit, although all thought the time was worthwhile [34].

Several studies ranked or rated individual toolkit components and found variation in the utility of different components [17, 26, 31, 35, 49, 63, 65, 85, 89]. For example, one study reported that 29% of respondents found ICD codes and reference articles the most useful tools in a pediatric obesity toolkit [35]. One study reported a wide range of perceived usefulness across components (cost calculator 10%, patient health questionnaire 68%) [31], one study reported that all participants were satisfied with the algorithms while only 83% were satisfied with the included office strategies to improve screening [17], one indicated that the provided frameworks for implementation were helpful and that the major success element was alcohol hand rubs [26], and one study reported on videos as the most positively rated component among individual tools [49]. Four studies assessed how to improve the toolkit or which components were missing [31, 39, 62, 67].

Seventeen studies reported on barriers to staff implementing the toolkit [13, 24, 27, 43, 49, 59, 62, 74–76, 78, 79, 81, 84–86, 88]. Common cited barriers included time constraints [21, 24, 43, 59, 62, 65, 85, 86, 88], no pertinent personnel available [13, 27, 43, 74, 88], culture or institutional factors [27, 74, 75], limited resources or costs [13, 24, 27, 49, 74, 85, 86], competing demands [65, 75, 86], or dissatisfaction with the toolkits content [24, 62]. Some study explored facilitators and barriers such as support from leadership [59] or whether a component could be implemented quickly and/or easily, especially when the tool or template was immediately available [24].

Individual studies reported ratings across dimensions such as ease of use [41], estimated time spent using the toolkit [48], or which intervention components (e.g., patient partnering) were most difficult to implement [25].

### Key question 3: what is the effectiveness of published quality improvement toolkits?

We systematically extracted any information reported on process, provider, and patient effects.

#### Process effects

More than half of the included studies (57%) reported specific effects on clinical practice such as procedural changes [12–14, 16, 19–21, 24–26, 34–40, 42, 43, 45–47, 51, 52, 55, 56, 58, 59, 61–71, 74, 76, 78, 81–83, 85, 87]. In most cases, studies reported on the adherence to procedures suggested in the toolkit such as appointing a pediatric physician coordinator [13], counseling children and their families on weight and healthy lifestyles [86], and documenting symptom assessment for mobility impairment or falls [43]. The evidence table shows the range of findings reported in individual studies.

The randomized controlled trials (RCTs) reported positive results for process outcomes. A Fall TIPS toolkit study reported patients on the intervention units were more likely to have fall risk documented (*p* < .0001) [16]. An evaluation of the America-on-the-Move toolkit reported control providers provided nutrition counseling to overweight patients in 40 to 49% of visits compared to 30 to 39% in intervention providers but the statistical significance of the difference was not reported [39]. Intervention practices increased vaccination rates more than controls (*p* = 0.34) in a study that used the 4-Pillars Toolkit for Increasing Childhood Influenza Immunization [46]. One RCT and five controlled trials did not report procedure outcomes [18, 28, 32, 44, 77, 87]. One controlled trial indicated that the control group missed or weakly addressed on average 3.3 of nine key intensive care unit care but no significant test was reported [81].

Pre-post studies that compared baseline and follow-up performances and that reported a statistical significance test for the difference were generally positive but there was variation across different procedures. The median percent of patients with asthma using inhaled corticosteroids, patients with an action plan, and patients using spirometry increased statistically significantly after introducing the Colorado Asthma Toolkit [19]. In another study, performance on quality measures for antenatal steroid administration increased from 77 to 100% (< .01) [36]. The Fall TIPS toolkit was associated with an increase from 1.7 to 2.0 in the mean number of fall risk assessments completed per day 1 month after implementation (*p* < .003) [61] [23]. An evaluation of an Acute Postoperative Pain Management Toolkit reported statistically significantly improvement in two pain management indicators (patients who had a pain score used to assess pain at rest and movement, patients with documented pain management plan) [12]. Compared to baseline, nurses were almost twice as likely to advise smokers to quit (*p* < .005), and more likely to assess willingness to quit, assist with a quit plan, and to recommend the smoking helpline (*p* < .0001) 6 months after the implementation of a smoking cessation toolkit [83]. One study showed a significant increase (*p* = .03) in the number of patients reporting a dialogue about weight management [82].

Five pre-post studies with numerical data reported mixed results. The Bright Futures Training Intervention Project toolkit was associated with statistically significant increases in the use of a preventive service prompting system and the proportion of families asked about special health care needs, but not the proportion of children who received a structured developmental assessment [24]. A toolkit to support multiple sclerosis management was associated with some improvements in documented assessments and care plan documentation [43]. A pre-post study evaluating the 4 Pillars Toolkit found different results for the different vaccines and different sites [51]. Medication list but not allergy list accuracy improved after introducing the Ambulatory Patient Safety Toolkit [25]. Another study showed improvements in documentation of BMI percentile (*p* < .05), education and counseling (*p* < .05), accurate diagnosis of overweight or obesity (*p* < .05) but a decrease in documentation of blood pressure readings (*p* < .05) [64].

#### Provider effects

Forty percent included studies reported data from healthcare providers. Studies did not separate effects of toolkits versus other intervention elements when these were present. With some exceptions [18, 21, 26, 32, 34, 43, 48, 50, 60, 63, 65, 66, 68, 70, 73, 76, 78, 88], provider effects were studied using post only designs such as asking providers to describe the effects of the toolkit.

The majority of these studies included self-reported provider behavior changes or intentions [15, 17, 18, 22, 26, 30, 31, 33, 48, 50, 65, 68, 69, 72, 73, 75, 76]. Among studies reporting numerical findings, results ranged from 60% of respondents indicating they had somewhat changed their practice after viewing study resources [31] to 95% of providers stating that they would increase use of fecal immunochemical tests for patients ineligible for or refusing colonoscopy [17].

Studies also reported on healthcare provider attitudes [21, 26, 32, 43, 44, 49, 52, 60, 62, 63, 68, 69, 76, 78, 86]. For example, one study reported 76 to 84% of providers indicated that posters made staff think about their hand hygiene [26], one indicated that positive perceptions of the importance and usefulness of body mass index increased [21], one reported increased awareness of multiple sclerosis symptoms [43], one indicated that the impact on patients varied by site [52], and one found no difference in safety perception, culture of safety awareness, sensitivity, and competence behaviors between the toolkit exposed and control groups [32].

Some studies reported on self-reported provider knowledge, confidence and perceived competence, and results were positive throughout [30, 34, 44, 60, 62, 65, 67–70, 76]. Examples included that 77% of users agreed that their knowledge of health literacy was improved [34], participants’ ratings of knowledge gain and confidence in geriatric competencies improved [30], and provider confidence in the ability to provide physical activity and exercise counseling and greater knowledge about physical activity improved [44].

Three studies tested provider knowledge; one found no difference in general concussion knowledge between intervention and control groups but intervention physicians were less likely to recommend next day return to play after concussion [18]. A congenital heart disease toolkit improved knowledge (pretest average score 71% improved to 93%, *p* < .0001) [66], and one study documented that only three of the ten knowledge-based questions were answered correctly by more than 85% of participants on the pre-test but all ten questions were answered correctly by at least 95% of participants on the post-test after implementing a patient safety toolkit [88]. One study reported that adherence to targeted provider behaviors increased significantly for 62% of behaviors but not for counselor competence [50].

#### Patient effects

We identified 22 studies (29% of all included studies) that reported on patient outcomes, the primary outcome of the review. While some studies reported on patient health [10, 28, 33, 40, 44, 45, 52, 55, 57, 61, 77, 78, 84, 87], others reported on patient satisfaction with the toolkit or individual tools [26, 48, 64, 79, 85], or other patient outcomes such as satisfaction with care processes [60, 66, 86].

None of the RCTs reported on patient outcomes. The studies with concurrent control groups reported mixed results within and across studies. A controlled trial (12/16 QI-MQCS domain criteria met) evaluating the impact of shared decision making supported by a toolkit reported higher asthma quality of life (MD 0.9; CI 0.4, 1.4) and fewer asthma control problems (MD − 0.9; CI − 1.6, − 0.2) in the intervention group [87]. Another controlled trial (13/16 QI-MQCS) found a single counseling appointment using the Diabetes Physical Activity and Exercise Toolkit was not associated with significant changes in physical activity or clinical outcomes compared to standard care [44]. The Guidelines Applied in Practice–Heart Failure Tool Kit was associated with a reduction in the baseline-adjusted 30-day readmission rate but not 30-day mortality comparing the toolkit and a control cohort (7/16 QI-MQCS) [28]. A state perinatal quality collaborative reported that women in hospitals engaged in the initiative experienced a 21% reduction in severe maternal morbidity among hemorrhage patients compared to baseline while the non-participating California hospitals showed no changes (1.2% reduction, n.s.); the collaborative used a toolkit to disseminate the intervention bundle (13/16 QI-MQCS) [77].

Two pre-post studies reported a statistically significant reduction in the incidence rate of hospital-acquired infections. One study (14/16 QI-MQCS) reported a reduction in carbapenemase-producing Enterobacteriaceae outbreaks and no further occurrence of extensively drug-resistant *Acinetobacter baumannii* after introducing a CDC toolkit and additional safety procedures such as limiting access to rooms and common areas [40]. A study (13/16 QI-MQCS) evaluating the AORN toolkit accompanying the Universal Protocol for Correct Site Surgery reported that after the introduction of the protocol, the rate of wrong site surgery increased initially [33]. A study (3/16 QI-MQCS) evaluating a toolkit on elimination of non-medically indicated (elective) deliveries before 39 weeks gestational age indicated that there were no transfers to the neonatal intensive care unit compared to five transfers pre-intervention (*p* < .022) for non-medically indicated deliveries between 37/0 and 38/6 pregnancy weeks [55]. A study (13/16 QI-MQCS) evaluating a toolkit-based intervention to reduce central line associated bloodstream infections reported that the rate of infections decreased by 24% (*p* = .001) [84]. The remaining pre-post studies reported improved patient outcomes for some or all outcomes but the statistical significance was not reported (QI-MQCS assessments ranged from four to 14 domain criteria met) [10, 45, 52, 57, 61, 78].

#### Comparison of original intervention and toolkit supported effects

For six toolkits, results of the initial intervention that led to the development of the toolkit had been published. However, no definitive comparison between initial intervention and success of spreading the intervention via the toolkit could be achieved due to the paucity of data and differences in study designs and metrics.

A toolkit intervention to reduce central line associated bloodstream infections referred to a published RCT that had established the effectiveness of the interventions for intensive care unit patients. The toolkit intervention established a 24% infection rate reduction and the authors highlighted the routine practice evaluating achieved results that are comparable to the original trial results (modeled hazard ratio 0.63, 2.1 vs 3.4 isolates per 1000 days, *p* = .01) [84, 91]. A toolkit for postoperative pain management was based on an initiative that had achieved a 13% increase in preoperative patient education and 19% increase in patients with at least one documented postoperative pain score [92]. Corresponding results associated with toolkit-based spread showed a 28% increase of patients with pain assessments [12]. An electronic fall prevention toolkit was tested in two studies [16, 23] and results were also available from the development of the toolkit. The intervention was associated with a reduced rate of falls [93] but the RCT testing the toolkit-assisted spread evaluation did not report on patient outcomes and it is unclear whether the toolkit can replicate the results in different organizations. An antenatal corticosteroid therapy toolkit was developed as part of a quality care collaborative that reported that antenatal steroid administration rate increased from 76 to 86% [94]. The results associated with implementation of the later developed toolkit was 100% performance of state quality measures for antenatal steroid performance administration compared to 77% at baseline [36]. The Project Re-Engineered Discharge toolkit was associated with a readmission rate reduction of 32% compared to baseline but the 30-day readmission rate was not reported [45]. The original hospital discharge program reported reduced hospital utilization within 30 days of discharge in an RCT comparing to usual care (30-day readmission rate 0.149 vs 0.207) [95]. The four pillars toolkit for influenza and pneumococcal vaccinations has been evaluated in multiple publications [46, 51]. The development phase of the toolkit has also been documented, but reported information was limited to areas of improvement that resulted in the final tool [96]. A relapse prevention group counseling toolkit was associated with counselor adherence to toolkit content in 13 out of 21 targeted behaviors [50]. Data from the development phase of the toolkit were available but not directly comparable; one study reported significant improvements in content adherence after 3 h of training [97], the other study reported on acceptability and sustainability of toolkit use [98].

## Discussion

There are few methods other than toolkits to document complex healthcare interventions or to support their use outside of initial intervention sites, yet little theoretical or empirical literature addresses toolkit use. We reviewed over a decade of published evaluations of toolkits used as a method for spreading quality improvement interventions for healthcare delivery organizations. This review documents the frequency of key toolkit elements and the effects of using publicly available toolkits. We hope this review will stimulate further thought on use of toolkits, on toolkit evaluation, and on toolkit reporting.

The toolkits and their evaluations included highly variable sets of information. Among toolkit elements, the toolkits we identified most commonly included introductory and implementation information (e.g., educational material for staff) and healthcare provider tools for clinical practice (e.g., care plans); and two-thirds included material for patients (e.g., information leaflets). Among evaluation elements, studies most often rated satisfaction with the toolkit and/or ratings of the utility of individual tools; while satisfaction was usually high, usefulness ratings varied. Rates of toolkit uptake across eligible users could provide invaluable information on issues such as ease of adoption, needed toolkit improvements, or equity in terms of making toolkit benefits accessible to all eligible subjects. However, only half of studies reported on toolkit uptake; these studies typically showed varied uptake between providers and/or settings. The reported information on toolkit uptake also often lacked a denominator or point of reference, such as the time period of tracked downloads, how many providers or sites were eligible, or how the uptake compared to other toolkits. A qualitative study of clinic and community members perspectives on intervention toolkits highlighted that information on the use of the toolkit is critical; simply disseminating toolkits does not guarantee its use [99].

We found the existing evidence base on toolkit effectiveness to be very limited despite the substantial number of publications on toolkits. We looked for effectiveness information not only in the searched toolkit publication, but in any related studies of the toolkit. While more than half of the included studies reported on adherence to clinical procedures, only some assessed effects on healthcare providers. In addition, the existing evidence base for healthcare provider effects associated with toolkits focuses on self-reported behavior changes or intentions. While reported results were positive and often indicated substantial improvement, objective tests for behavior changes are largely absent from the literature.

Quality improvement theory emphasizes the importance of completing the intervention and evaluation cycle through an assessment of impacts on patient care and outcomes, but we found few such assessments. Few studies reported on patient outcomes and there is a lack of evaluations showing improved health outcomes to be associated with toolkits. Toolkits are commonly aimed at intervention spread; however, the evidence base for their effectiveness for this purpose is limited. Identified RCTs reported positive results for spread sites; however, the number of high-level evidence studies that allow strong effectiveness conclusions is small. While pre-post assessments tended to be positive, studies with concurrent control groups reported mixed results within and across studies. More evaluations of toolkit effects on patient care and outcomes are needed to determine whether the use of toolkits translates into improvements for patients.

Throughout, study results were often insufficiently reported and the assessed outcomes were very diverse. Furthermore, the identified studies were often not designed to assess the effect of the toolkit per se because the intervention included other components in addition to the toolkit. Use of stronger study designs for assessing toolkit effectiveness as a method of spread, such as presenting comparisons to the status prior to their implementation or to a control group, would increase the value of toolkit spread studies.

An optimistic review interpretation is that studies of toolkit effectiveness showed no deterioration when the toolkit was applied in new settings. Very few published studies are available that directly address this comparison, however. While some studies described the development of the toolkit as following a successful intervention implementation, very few studies reported numerical results that allowed a direct comparison between the original intervention and the results of facilitating the spread of the intervention through a toolkit.

The reported detail in the included studies varied widely and no study met all of the QI-MQCS criteria, a critical appraisal tool for quality improvement evaluation publications [9]. We included studies reported in abbreviated form such as conference abstracts, hence some information important to practitioners was sometimes not available but a large majority of studies reported a rationale for implementing the toolkit in their organization and provided information on the intended change in organizational or provider behavior that they were aiming to achieve with the toolkit. We anticipate that future evaluations of toolkits can increase their impacts by focusing on the information most likely to be useful to potential users or to fellow developers of toolkits. These include, for example, uptake rates, resources required for toolkit adoption, and resources required for toolkit maintenance. Information on toolkit adaptations required for adoption in different organizational contexts would also be helpful. Furthermore, while the reported satisfaction with the toolkits was generally reported to be positive, there were often large variations in ratings of the utility of specific components or tools. Further evaluations should consider the merits of assessing individual toolkit components in addition to evaluating the toolkit as a whole.

There is no standard definition of a toolkit and guidance for toolkit developers and users is only beginning to emerge [100]. A strength of this review is our focus on quality improvement interventions in healthcare, using a definition based on our prior experience with quality improvement and implementation research [8, 9, 101–106]. A limitation is that we used a self-applied definition of what constitutes a toolkit and we only searched for studies using the term “toolkit.” A broader review of tools and of similar resources not referenced as “toolkits” would be an important addition to the literature.

The included studies and evaluated toolkits were very heterogeneous, limiting generalizable conclusions that can be drawn across studies, and the diversity is reflected in the evidence and summary tables. Nonetheless, the review was limited to publications and toolkits that used the term “toolkit” and we included only toolkits reported in published literature. Our review included gray literature in that we purposefully included conference abstracts and dissertations; we know, however, that we missed information on unpublished use of toolkits especially in large organizations. Furthermore, the number of studies contributing the effectiveness key question was limited, in particular studies reporting on the primary outcome—patient health. Limitations in the quality of evidence hindered more detailed analyses and conclusions, including answers to the question whether toolkits developed in another context can achieve the same results in a new context.

Finally, our review concentrated on the large number of toolkits that are currently publicly available, free of charge or for purchase. Toolkits not explicitly designed for ongoing spread (e.g., toolkit distributions for one-time interventions) were beyond the scope of the review. A prior systematic review on toolkits reported limited evidence for toolkits as a general intervention component or implementation strategy. Of eight methodologically acceptable evaluations identified by the review, six showed at least partial effectiveness in changing clinical outcomes; however, the review concluded that more rigorous study designs were needed to explain the factors underlying toolkit effectiveness and successful implementation [107].

## Conclusions

This review documents over a decade of evaluations of publicly available quality improvement toolkits and provides insight into the components, the uptake, and the current evidence base of the effectiveness of this tool for spread. Available uptake data are limited but indicate variability. High satisfaction with toolkits can be achieved but the usefulness of individual tools may vary. The existing evidence base on the effectiveness of toolkits remains limited. While emerging evidence indicates positive effects on clinical processes, more research on toolkit value and what affects it is needed, including linking toolkits to objective provider behavior measures and patient outcomes. Considering the potential importance of toolkits as a method for maximizing the impacts of healthcare improvement interventions, a stronger research focus on the conduct and reporting of toolkit intervention and evaluation components is critical.

## Additional files

Additional file 1: PRISMA checklist. (DOCX 26 kb)

Additional file 2: Appendix A: Search terms. Appendix B: Identified publicly available toolkits. Appendix C: Toolkits included in the review. Appendix D: Critical Appraisal QI-MQCS. (DOCX 199 kb)

## Data Availability

The data are displayed in the in-text tables and the online-only appendix. We can convert the data to a spreadsheet upon request.

## Abbreviations

AHRQ: Agency for Healthcare Research and Quality
AORN: Association of Perioperative Registered Nurses
CDC: Centers for Disease Control and Prevention
CMS: Centers for Medicare and Medicaid Services
ECRI: Emergency Care Research Institute
IHI: Institute for Healthcare Improvement
QI-MQCS: Quality Improvement Minimum Quality Criteria Set
RCT: Randomized controlled trials
RWJF: Robert Wood Johnson Foundation
VA: Department of Veterans Affairs (VA)
WHO: World Health Organization
