# Supplementary material for: Spread tools: a systematic review of components, uptake, and effectiveness of quality improvement toolkits
Source: Implement Sci. 2019 Aug 19;14:83. doi: 10.1186/s13012-019-0929-8 (PMC6701087; doi:10.1186/s13012-019-0929-8)
Supplement: Supplementary file 2 — Appendix A: Search terms. Appendix B: Identified publicly available toolkits. Appendix C: Toolkits included in the review. Appendix D: Critical Appraisal QI-MQCS. (DOCX 199 kb) [file 13012_2019_929_MOESM2_ESM.docx]

# Additional files

## Appendix A: Search terms

**PubMed**

Run 5/1/2018

Limits: 2005 -present; English

(toolkit[tiab] OR "tool kit"[tiab])

NOT

(genome or gene OR DNA OR genomic OR molecular OR genetic OR "linkage analysis" OR spectrometry[ti] OR spectrometer OR spectrometric OR NMR OR bioinformatics OR peptide OR protein[ti] OR livestock OR agriculture OR Geant4 OR "Insight Toolkit" OR "Visualization Toolkit" OR "Open Multi-Processing")

NOT

Other Animals; rat OR rats OR butterfly OR butterflies OR Rodent OR rodents OR mice OR mouse OR fish

Note: The MeSH term “Diffusion of innovation” (The broad dissemination of new ideas, procedures, techniques, materials, and devices and the degree to which these are accepted and used. Year introduced: 1991(1980)) was not used because not all known toolkit applications studies used this tag.

**CINAHL**

Run 5/1/2018

Limiters: Date of Publication: 20051001-; Human, Language: English

TI "tool kit" OR AB "tool kit" OR TI toolkit OR AB toolkit OR TI tool N1 kit OR AB tool N1 kit

NOT

TI ( genome or gene OR DNA OR genomic OR molecular OR genetic OR "linkage analysis" OR spectrometry OR spectrometer OR spectrometric OR NMR OR bioinformatics OR peptide OR protein OR livestock OR agriculture OR Geant4 OR “Insight Toolkit” OR “Visualization Toolkit” OR “Open Multi-Processing” ) OR AB ( genome or gene OR DNA OR genomic OR molecular OR genetic OR "linkage analysis" OR spectrometry OR spectrometer OR spectrometric OR NMR OR bioinformatics OR peptide OR protein OR livestock OR agriculture OR Geant4 OR “Insight Toolkit” OR “Visualization Toolkit” OR “Open Multi-Processing” )

**Web of Science**

Run 5/1/2018

Indexes=SCI-EXPANDED, CPCI-S Timespan=2005-2018

AND LANGUAGE: (English)

TOPIC: (“tool kit” OR toolkit)

NOT

TOPIC:( genome or gene OR DNA OR genomic OR molecular OR genetic OR "linkage analysis" OR spectrometer OR spectrometric OR NMR OR bioinformatics OR peptide OR livestock OR agriculture OR Geant4 OR “Insight Toolkit” OR “Visualization Toolkit” OR “Open Multi-Processing”)

OR

TITLE:(spectrometry) OR TITLE:(protein)

[excluding] WEB OF SCIENCE CATEGORIES: ( ENGINEERING ELECTRICAL ELECTRONIC OR COMPUTER SCIENCE SOFTWARE ENGINEERING OR WATER RESOURCES OR COMPUTER SCIENCE INFORMATION SYSTEMS OR COMPUTER SCIENCE THEORY METHODS OR COMPUTER SCIENCE INTERDISCIPLINARY APPLICATIONS OR RADIOLOGY NUCLEAR MEDICINE MEDICAL IMAGING OR COMPUTER SCIENCE ARTIFICIAL INTELLIGENCE OR ANTHROPOLOGY OR ENERGY FUELS OR BIOCHEMICAL RESEARCH METHODS ) AND [excluding] WEB OF SCIENCE CATEGORIES: ( CHEMISTRY MULTIDISCIPLINARY OR PHYSICS MULTIDISCIPLINARY ) AND [excluding] WEB OF SCIENCE CATEGORIES: ( OCEANOGRAPHY OR GEOSCIENCES MULTIDISCIPLINARY ) AND [excluding] WEB OF SCIENCE CATEGORIES: ( ENVIRONMENTAL SCIENCES OR BIOCHEMISTRY MOLECULAR BIOLOGY ) AND [excluding] WEB OF SCIENCE CATEGORIES: ( ENGINEERING AEROSPACE OR ENGINEERING INDUSTRIAL OR ENGINEERING CHEMICAL ) AND [excluding] WEB OF SCIENCE CATEGORIES: ( ENGINEERING MECHANICAL )

## Appendix B: Identified publicly available toolkits

| **Agency** | **Publisher** | **Toolkit Name** | **Link** |
| --- | --- | --- | --- |
| AHRQ | AHRQ | Carbapenem-Resistant Enterobacteriaceae (CRE) Control and Prevention Toolkit | <http://www.ahrq.gov/cretoolkit> |
| AHRQ | AHRQ | Comprehensive Unit-based Safety Program (CUSP) Toolkit | <http://www.ahrq.gov/professionals/education/curriculum-tools/cusptoolkit/index.html> |
| AHRQ | AHRQ | Health Information Exchange (HIE) Evaluation Toolkit | <http://healthit.ahrq.gov/health-it-tools-and-resources/health-information-exchange-hie-evaluation-toolkit> |
| AHRQ | AHRQ | Health IT Evaluation Toolkit | <http://healthit.ahrq.gov/health-it-tools-and-resources/health-it-evaluation-toolkit> |
| AHRQ | AHRQ | Health Literacy Universal Precautions Toolkit | <http://www.ahrq.gov/professionals/quality-patient-safety/quality-resources/tools/literacy-toolkit/index.html> |
| AHRQ | AHRQ | Implementing the Care Model and Business Strategies in the Safety Net | <http://www.ahrq.gov/professionals/prevention-chronic-care/improve/system/pfhandbook/mod18.html> |
| AHRQ | AHRQ | Improving Your Office Testing Process: A Toolkit for Rapid-Cycle Patient Safety and Quality Improvement | <http://www.ahrq.gov/professionals/quality-patient-safety/quality-resources/tools/office-testing-toolkit/index.html> |
| AHRQ | AHRQ | Informed Consent and Authorization Toolkit for Minimal Risk Research | <http://www.ahrq.gov/funding/policies/informedconsent/index.html> |
| AHRQ | AHRQ | Integrating Primary Care Practices and Community-based Resources to Manage Obesity: A Bridge-building Toolkit for Rural Primary Care Practices | <http://www.ahrq.gov/professionals/prevention-chronic-care/improve/community/obesity-pcpresources/obpcp-quick.html> |
| AHRQ | AHRQ | Medications at Transitions and Clinical Handoffs (MATCH) Toolkit for Medication Reconciliation | <http://www.ahrq.gov/professionals/quality-patient-safety/patient-safety-resources/resources/match/index.html> |
| AHRQ | AHRQ | Present on Admission (POA) Indicator Toolkit | <http://hcup-us.ahrq.gov/datainnovations/clinicaldata/poaback.jsp> |
| RAND, ECRI, Boston University School of Public Health | AHRQ | Preventing Falls in Hospitals: A Toolkit for Improving Quality of Care | <http://www.ahrq.gov/professionals/systems/hospital/fallpxtoolkit/index.html> |
| AHRQ | AHRQ | Preventing Pressure Ulcers in Hospitals: A Toolkit for Improving Quality of Care. | <http://www.ahrq.gov/professionals/systems/long-term-care/resources/pressure-ulcers/pressureulcertoolkit/index.html> |
| AHRQ | AHRQ | Quality Indicators™ Toolkit for Hospitals | <http://www.ahrq.gov/professionals/systems/hospital/qitoolkit/index.html> |
| AHRQ | AHRQ | Re-Engineered Discharge (RED) Toolkit | <http://www.ahrq.gov/professionals/systems/hospital/red/toolkit/index.html> |
| AHRQ | AHRQ | Toolkit for Implementing the Chronic Care Model in an Academic Environment | <http://www.ahrq.gov/professionals/education/curriculum-tools/chroniccaremodel/index.html> |
| AHRQ | AHRQ | Toolkit for Reduction of Clostridium difficile Infections Through Antimicrobial Stewardship | <http://www.ahrq.gov/professionals/quality-patient-safety/patient-safety-resources/resources/cdifftoolkit/cdiffover.html> |
| AHRQ | AHRQ | Tools for Reducing Central Line-Associated Blood Stream Infections | <http://www.ahrq.gov/professionals/education/curriculum-tools/clabsitools/index.html> |
| AHRQ | AHRQ | Transitioning Newborns from NICU to Home: A Resource Toolkit | <http://www.ahrq.gov/professionals/systems/hospital/nicu_toolkit/index.html> |
| AHRQ | AHRQ | Venous Thromboembolism Safety Toolkit: A Systems Approach to Patient Safety | <http://www.ahrq.gov/downloads/pub/advances2/vol3/advances-zierler_81.pdf> |
| AHRQ | AHRQ | Workflow Assessment for Health IT Toolkit | <http://healthit.ahrq.gov/health-it-tools-and-resources/workflow-assessment-health-it-toolkit> |
| AHRQ Innovations | Cedar Rapids Healthcare Alliance | A Comprehensive List of Tools to Implement the Anticoagulation Management System | <http://www.crhealthcarealliance.org/Toolkit/Default.aspx?menuID=5> |
| AHRQ Innovations | Massachusetts General Hospital | A Toolkit for the Well Child Screening of Military Children | <http://www.homebaseprogram.org/community-education/~/media/DDA3707BC4A648E89F1A7C619C48FC28.pdf> |
| AHRQ Innovations | California Academy of Family Physicians | Addressing Language Access Issues in Your Practice: A Toolkit for Physicians and Their Staff Members | <http://www.calendow.org/uploadedFiles/language_access_issues.pdf> |
| AHRQ Innovations | Center for Technology and Aging; Public Health Institute | ADOPT Toolkit: Helping Organizations Design and Implement Connected Health Technology | <http://toolkit.techandaging.org/> |
| AHRQ Innovations | Healthcare Information and Management Systems Society | Ambulatory HIE Toolkit | <http://www.himss.org/library/health-information-exchange/ambulatory-hie/toolkit> |
| AHRQ Innovations | Greater New York Hospital Association; United Hospital Fund | Antimicrobial Stewardship Toolkit | [http://www.gnyha.org/6763/Default.aspx](http://www.innovations.ahrq.gov/disclaimer.aspx?redirect=http://www.gnyha.org/6763/Default.aspx) |
| AHRQ Innovations | Forum of ESRD Networks’ Medical Advisory Council | Assurance of Diabetes Care Coordination Toolkit | <http://esrdnetworks.org/mac-toolkits/download/download-write-able-forms-assurance-of-diabetes-care-coordination-toolkit-1/assurance-of-diabetes-care-coordination-toolkit/view> |
| AHRQ Innovations | Family Caregiver Alliance | Caregivers Count Too!: A Toolkit to Help Practitioners Assess the Needs of Family Caregivers | <https://caregiver.org/caregivers-count-too-toolkit> |
| AHRQ Innovations | American Academy of Pediatrics; National Initiative for Children's Healthcare Quality; University of Chapel Hill for its Center for Children's Healthcare Improvement | Caring for Children with ADHD (Attention-Deficit/Hyperactivity Disorder): A Resource Toolkit for Clinicians | <http://www.nichq.org/childrens-health/adhd/resources/adhd-toolkit> |
| AHRQ Innovations | Holy Cross Hospital | Chronic Disease Self-Management Program: A Toolkit for Hospitals | <http://www.ncoa.org/improve-health/center-for-healthy-aging/content-library/Hospital-Toolkit-MD-2012.pdf> |
| AHRQ Innovations | Johns Hopkins University Quality & Safety Research Group | CLABSI Elimination Toolkit | <http://www.onthecuspstophai.org/on-the-cuspstop-bsi/toolkits-and-resources/#clabsi> |
| AHRQ Innovations | The Joint Commission | CLABSI Toolkit – Preventing Central Line–Associated Bloodstream Infections: Useful Tools, An International Perspective | [http://www.jointcommission.org/Topics/Clabsi_toolkit.aspx](http://www.innovations.ahrq.gov/disclaimer.aspx?redirect=http://www.jointcommission.org/Topics/Clabsi_toolkit.aspx) |
| AHRQ Innovations | Arizona Hospital and Healthcare Association | Color-coded Wristband Standardization in Arizona Implementation Toolkit | [http://static.squarespace.com/static/52a62872e4b0cfb0c7cce4b2/t/52acf0a8e4b0cfa18156df35 /1387065512472](http://static.squarespace.com/static/52a62872e4b0cfb0c7cce4b2/t/52acf0a8e4b0cfa18156df35/1387065512472) |
| AHRQ Innovations | American Medical Association | Communication Climate Assessment Toolkit | [http://www.ama-assn.org/ama/pub/physician-resources/medical-ethics/the-ethical-force-program/patient-centered-communication/organizational-assessment-resources.page](http://www.innovations.ahrq.gov/disclaimer.aspx?redirect=http://www.ama-assn.org/ama/pub/physician-resources/medical-ethics/the-ethical-force-program/patient-centered-communication/organizational-assessment-resources.page) |
| AHRQ Innovations | Academy for Continued Healthcare Learning (ACHL) | Constructing an Adaptive Care Model for the Management of Disease-Related Symptoms Throughout the Course of Multiple Sclerosis | <http://achlpicme.org/ms/CMEInfo.aspx> |
| AHRQ Innovations | University of Wisconsin-Madison School of Medicine & Public Health; William S Middleton Memorial Veterans Hospital | Coordinated-Transitional Care Toolkit | <http://www.hipxchange.org/C-trac> |
| AHRQ Innovations | Massachusetts Child Psychiatry Access Project | CRAFFT (Car, Relax, Alone, Forget Friends, Trouble) Toolkit | <http://www.mcpap.com/pdf/CRAFFT%20Screening%20Tool.pdf> |
| AHRQ Innovations | Children's Hospital Boston | Developmental Screening Tool Kit for Primary Care Providers | <http://www.developmentalscreening.org/> |
| AHRQ Innovations | University of Colorado Anschutz Medical Campus, School of Medicine, Behavioral Health and Wellness Program | DIMENSIONS: Tobacco Free Toolkit for Healthcare Providers | <http://www.bhwellness.org/resources/toolkits/> |
| AHRQ Innovations | University of Colorado Anschutz Medical Campus, School of Medicine, Behavioral Health and Wellness Program | DIMENSIONS: Well Body Toolkit for Healthcare Providers | <http://www.bhwellness.org/resources/toolkits/> |
| AHRQ Innovations | Banner Health | Door-to-Doc Patient Safety Toolkit | <http://www.bannerhealth.com/About+Us/Innovations/DoortoDoc/Download+D2D+Toolkit.htm> |
| AHRQ Innovations | Academy for Continued Healthcare Learning (ACHL) | Enhancing the Management of Neuropathic Pain in the Long-Term Care Setting | <http://achlpicme.org/ltc/CMEInfo.aspx> |
| AHRQ Innovations | Healthcare Information and Management Systems Society | Enterprise HIE Toolkit | <http://www.himss.org/library/health-information-exchange/enterprise-hie/toolkit?navItemNumber=16139> |
| AHRQ Innovations | National Association of Nurse Practitioners In Women’s Health | Free HPV (Human Papillomavirus) Testing Toolkits for Clinicians | <http://www.npwh.org/i4a/pages/index.cfm?pageid=3357> |
| AHRQ Innovations | Nancy Steinberg Warren, MS, CGC | Genetic Counseling Cultural and Linguistic Competence Toolkit | <http://geneticcounselingtoolkit.com/default.htm> |
| AHRQ Innovations | Great Plains Telehealth Resource and Assistance Center | Great Plains Telehealth Resource and Assistance Center Toolkit | [http://www.gptrac.org/resource-center/proven-practices-and-models/](http://www.innovations.ahrq.gov/disclaimer.aspx?redirect=http://www.gptrac.org/resource-center/proven-practices-and-models/) |
| AHRQ Innovations | Healthcare Information and Management Systems Society | Health Information Exchange (HIE) Toolkit | <http://www.himss.org/library/health-information-exchange/toolkit> |
| AHRQ Innovations | eHealth Initiative | Health Information Exchange Toolkit | [http://www.ehidc.org/hie-toolkit](http://www.innovations.ahrq.gov/disclaimer.aspx?redirect=http://www.ehidc.org/hie-toolkit) |
| AHRQ Innovations | Stratis Health | Health Information Technology Toolkit for Home Health Agencies | <http://www.stratishealth.org/expertise/healthit/homehealth/index.html> |
| AHRQ Innovations | Stratis Health | Health Information Technology Toolkit for Physician Offices | <http://www.stratishealth.org/expertise/healthit/clinics/clinictoolkit.html> |
| AHRQ Innovations | Johns Hopkins Bloomberg School of Public Health | Hospital at Home Toolkit | <http://www.hospitalathome.org/develop-your-program/toolkit.php> |
| AHRQ Innovations | American Cancer Society; Iowa Cancer Consortium; Iowa Get Screened; Upper Midwest Public Health Training Center | How To Increase Cancer Screening Rates: A Quality Improvement Toolkit for Busy Office Practices | <http://www.canceriowa.org/Files/Health-Provider-Resources/Toolkit-for-Busy-Practices.aspx> |
| AHRQ Innovations | National Colorectal Cancer Roundtable; Thomas Jefferson University Department of Family Medicine | How To Increase Colorectal Cancer Screening Rates in Practice: A Primary Care Clinician’s Evidenced-Based Toolbox and Guide | [http://nccrt.org/about/provider-education/crc-clinician-guide/](http://www.innovations.ahrq.gov/disclaimer.aspx?redirect=http://nccrt.org/about/provider-education/crc-clinician-guide/) |
| AHRQ Innovations | American Association of Critical-Care Nurses; Vanderbilt University | Implementing the ABCDE Bundle at the Bedside | <http://www.aacn.org/wd/practice/content/actionpak/withlinks-ABCDE-ToolKit.content?menu=practice> |
| AHRQ Innovations | United States Breastfeeding Committee | Implementing The Joint Commission Perinatal Care Core Measure on Exclusive Breast Milk Feeding | <http://www.usbreastfeeding.org/HealthCare/HospitalMaternityCenterPractices/ToolkitImplementingTJCCoreMeasure/tabid/184/Default.aspx> |
| AHRQ Innovations | Center for Health Care Strategies, Inc. | Improving Asthma Care for Children Toolkit: Best Practices in Medicaid Managed Care | <http://www.chcs.org/media/IACC_Toolkit.pdf> |
| AHRQ Innovations | Academy for Continued Healthcare Learning (ACHL); Indiana University School of Medicine | Improving Outcomes for an Aging Population: Alzheimer's Treatment in Long-Term Care | [http://www.achlqicme.org/alz/Toolkit.aspx](http://www.innovations.ahrq.gov/disclaimer.aspx?redirect=http://www.achlqicme.org/alz/Toolkit.aspx) |
| AHRQ Innovations | Center for Health Care Strategies, Inc. | Improving Outcomes for Children in Child Welfare: A Medicaid Managed Care Toolkit | <http://www.chcs.org/media/Child_Welfare_Quality_Improvement_Collaborative_Toolkit.pdf> |
| AHRQ Innovations | Access Community Health Centers; University of Wisconsin - Madison | Integrated Primary Care Consulting Psychiatry Toolkit | [http://www.hipxchange.org/Access](http://www.innovations.ahrq.gov/disclaimer.aspx?redirect=http://www.hipxchange.org/Access) |
| AHRQ Innovations | Renal Physicians Association | Keeping Kidney Patients Safe: Patient Safety Improvement Toolkit | <http://www.kidneypatientsafety.org/toolkit.aspx> |
| AHRQ Innovations | Centers for Disease Control and Prevention, Lead Poisoning Prevention Program; Office of Refugee Resettlement | Lead Poisoning Prevention in Newly Arrived Refugee Children: Tool Kit | <http://www.cdc.gov/nceh/lead/Publications/RefugeeToolKit/Refugee_Tool_Kit.htm> |
| CMS Innovations | March of Dimes | Less than 39 weeks toolkit | <http://www.marchofdimes.com/professionals/less-than-39-weeks-toolkit.aspx> |
| AHRQ Innovations | Centers for Disease Control and Prevention Division of Healthcare Quality Promotion; Michigan Antibiotic Resistance Reduction (MARR) Coalition; Michigan Department of Community Health; Michigan Society for Infection Prevention and Control (MSIPC) | Long-term Care Tool Kit | <http://www.mi-marr.org/LTC_toolkit.php> |
| AHRQ Innovations | National Health Care for the Homeless Council | Medical Respite Tool Kit | <http://www.nhchc.org/resources/clinical/medical-respite/tool-kit/> |
| AHRQ Innovations | Tacoma-Pierce County Health Department | MRSA Toolkit for Outpatient Clinics/Offices | <http://www.tpchd.org/health-wellness-1/mrsa-methicillin-resistant-staphylococcus-aureus/mrsa-toolkit-outpatient-clinics-offices/> |
| AHRQ Innovations | Society of Hospital Medicine | Multi-Center Medication Reconciliation Quality Improvement Study (MARQUIS) Toolkit | [http://www.hospitalmedicine.org/Web/Quality___Innovation/Implementation_Toolkit/MARQUIS/Med_Rec_Resources_Medication_Reconciliation.aspx](http://www.innovations.ahrq.gov/disclaimer.aspx?redirect=http://www.hospitalmedicine.org/Web/Quality___Innovation/Implementation_Toolkit/MARQUIS/Med_Rec_Resources_Medication_Reconciliation.aspx) |
| AHRQ Innovations | National Rural Health Resource Center | National Rural Health Resource Center: Health Information Exchange Toolkit | [http://ruralcenter.org/rhitnd/hie-toolkit](http://www.innovations.ahrq.gov/disclaimer.aspx?redirect=http://ruralcenter.org/rhitnd/hie-toolkit) |
| AHRQ Innovations | Beth Israel Deaconess Medical Center; Geisinger Health System; University of Washington, Harborview Medical Center | OpenNotes Toolkit | <http://www.myopennotes.org/toolkit/> |
| AHRQ Innovations | Institute for Patient- and Family-Centered Care | Partnering with Patients and Families to Enhance Safety and Quality: A Mini Toolkit | <http://www.ipfcc.org/tools/Patient-Safety-Toolkit-04.pdf> |
| AHRQ Innovations | Integrated Behavioral Health Project (IBHP) | Partners In Health: Mental Health, Primary Care And Substance Use Inter-Agency Collaboration Tool Kit, 2nd Edition | <http://www.ibhp.org/uploads/file/IBHPIinteragency%20Collaboration%20Tool%20Kit%202013%20.pdf> |
| AHRQ Innovations | MacColl Institute for Healthcare Innovation | Reducing Care Fragmentation: A Toolkit For Coordinating Care | <http://www.improvingchroniccare.org/index.php?p=Care_Coordination&s=326> |
| AHRQ Innovations | Hospital Corporation of America | Safe Critical Care Project: Testing Improvement Strategies | <http://www.mc.vanderbilt.edu/root/vumc.php?site=HCA-VanderbiltPatientSafety> |
| AHRQ Innovations | Pacific Health Consulting Group | Sharing Specialty Services: A Business Guide and Toolkit for Community Clinics | <http://www.chcf.org/publications/2009/07/sharing-specialty-services-a-business-guide-and-toolkit-for-community-clinics> |
| AHRQ Innovations | Pennsylvania Patient Safety Authority; The Hospital and Healthsystem Association of Pennsylvania | Standardized Color-Coded Patient Wristbands | [http://www.tha.com/files/wristbands/toolkit/9-1-2009/complete-toolkit.pdf](http://www.innovations.ahrq.gov/disclaimer.aspx?redirect=http://www.tha.com/files/wristbands/toolkit/9-1-2009/complete-toolkit.pdf) |
| AHRQ Innovations | Health Foundation for Western and Central New York | Step Up to Stop Falls Toolkit™ | <http://www.hfwcny.org/Tools/Broadcaster/frontend/itemcontent.asp?reset=1&ItemID=13> |
| AHRQ Innovations | American Association of Critical-Care Nurses | Strategies for Managing Alarm Fatigue | [http://www.aacn.org/dm/practice/actionpakdetail.aspx?itemid=28337](http://www.innovations.ahrq.gov/disclaimer.aspx?redirect=http://www.aacn.org/dm/practice/actionpakdetail.aspx?itemid=28337) |
| AHRQ Innovations | Studer Group | Studer Group Toolkit: Patient Safety | <http://www.sgna.org/Portals/0/Events/Annual%20Course/2013%20-%20Austin/Karen%20Cook/Toolkit.Patient%20Safety.pdf> |
| AHRQ Innovations | Massachusetts Child Psychiatry Access Project | The Primary Care Behavioral Health Toolkit | <http://www.mcpap.com/pdf/PCCScreeningToolkitUpdate04292010.pdf> |
| AHRQ Innovations | University of Colorado Anschutz Medical Campus, School of Medicine, Behavioral Health and Wellness Program | Tobacco Free Toolkit for Community Health Facilities | <http://www.bhwellness.org/resources/toolkits/> |
| AHRQ Innovations | American College of Chest Physicians | Tobacco-Dependence Treatment Toolkit | <http://tobaccodependence.chestnet.org/> |
| AHRQ Innovations | Center of Excellence in Culturally Competent Mental Health; Nathan Kline Institute for Psychiatric Research; New York University School of Medicine | Toolkit for Modifying Evidence-Based Practices To Increase Cultural Competence | <http://ssrdqst.rfmh.org/cecc/index.php?q=node/86> |
| AHRQ Innovations | MedImmune; National Initiative for Children's Healthcare Quality | Toolkit for the Follow-Up Care of the Premature Infant | [http://www.nichq.org/resources/Premature_Infant_FollowUp_Toolkit.html](http://innovations.ahrq.gov/disclaimer.aspx?redirect=http://www.nichq.org/resources/Premature_Infant_FollowUp_Toolkit.html) |
| AHRQ Innovations | New York City Department of Health and Mental Hygiene | Trainings, Drills & Exercises: Hospital and Primary Care Centers Tabletop Toolki | <http://www.nyc.gov/html/doh/downloads/pdf/bhpp/bhpp-train-hospital-toolkit.pdf> |
| WHO | WHO | Integrated Management for Emergency and Essential Surgical Care (IMEESC) toolkit | <http://www.who.int/surgery/publications/imeesc/en/> |
| WHO | WHO | Age-friendly Primary Health Care (PHC) Centres Toolkit | <http://www.who.int/ageing/publications/upcoming_publications/en/> |
| WHO | WHO | Strengthening midwifery toolkit | <http://www.who.int/maternal_child_adolescent/documents/strenthening_midwifery_toolkit/en/> |
| WHO | WHO | BE AWARE toolkit for health professionals | <http://www.who.int/impact/news/beaware/en/> |
| WHO | WHO | Toolkit for delivering the 5A’s and 5R’s brief tobacco interventions in primary care | <http://www.who.int/tobacco/publications/smoking_cessation/9789241506953/en/> |
| WHO | WHO | Best practices for injections and related procedures toolkit | <http://www.who.int/injection_safety/9789241599252/en/> |
| WHO | WHO | Male circumcision quality assessment toolkit | <http://www.who.int/hiv/pub/malecircumcision/qa_toolkit/en/> |
| WHO | WHO | Monitoring and evaluation toolkit: HIV/AIDS, tuberculosis and malaria | <http://www.who.int/hiv/pub/me/me_toolkit2004/en/> |
| WHO | WHO | Protecting Healthcare Workers: Preventing needlestick injuries toolkit | <http://www.who.int/occupational_health/activities/pnitoolkit/en/> |
| WHO | WHO | WHO QualityRights Tool Kit | <http://www.who.int/mental_health/publications/QualityRights_toolkit/en/> |
| IHI | UnityPoint Health | Always Use Teach Back! Toolkit | <http://www.ihi.org/resources/Pages/Tools/AlwaysUseTeachBack!.aspx> |
| IHI | Purdue University PharmaTAP | Anticoagulant Toolkit: Reducing Adverse Drug Events | <http://www.ihi.org/resources/Pages/Tools/AnticoagulantToolkitReducingADEs.aspx> |
| IHI | Maryland Patient Safety Center | Condition Help (H) Toolkit | <http://www.ihi.org/resources/Pages/Tools/ConditionHelpToolkit.aspx> |
| IHI | IHI | Disclosure Toolkit and Disclosure Culture Assessment Tool | <http://www.ihi.org/resources/Pages/Tools/DisclosureToolkitandDisclosureCultureAssessmentTool.aspx> |
| IHI | Florida Hospital | Global Trigger Tool Implementation Toolkit | <http://www.ihi.org/resources/Pages/Tools/GlobalTriggerToolImplementationToolkit.aspx> |
| IHI | Health Communications | Health Communications Toolkits: Improving Readability of Patient Education Materials | <http://www.ihi.org/resources/Pages/Tools/HealthCommunicationsToolkitsImprovingReadabilityPtEdMaterials.aspx> |
| IHI | Kaiser Permanente | Infection Prevention Plus Measures Toolkit | <http://www.ihi.org/resources/Pages/Tools/InfectionPreventionPlusMeasuresToolkit.aspx> |
| IHI | Northwestern Memorial Hospital | MATCH Medication Reconciliation Toolkit | <http://www.ihi.org/resources/Pages/Tools/MATCHMedicationReconciliationToolkit.aspx> |
| IHI | North Carolina Center for Hospital Quality and Patient Safety | Medication Safety Reconciliation Toolkit | <http://www.ihi.org/resources/Pages/Tools/MedicationSafetyReconciliationToolKit.aspx> |
| IHI | IHI | Partnering in Self-Management Support: A Toolkit for Clinicians | <http://www.ihi.org/resources/Pages/Tools/SelfManagementToolkitforClinicians.aspx> |
| IHI | Child Health Corporation of America (CHCA) | Pediatric Trigger Toolkit: Measuring Adverse Drug Events in the Children’s Hospital | <http://www.ihi.org/resources/Pages/Tools/PediatricADETriggerToolkit.aspx> |
| IHI | IHI | Prevent Surgical Site Infection for Hip and Knee Arthroplasty Presentation Toolkit | <http://www.ihi.org/resources/Pages/Presentations/PreventSSIHipKneePresentationToolkit.aspx> |
| IHI | Kaiser Permanente | SBAR (Situation, Background, Assessment, Recommendation) Toolkit | <http://www.ihi.org/resources/Pages/Tools/SBARToolkit.aspx> |
| IHI | American Hospital Association | Strategies for Leadership: Patient-and Family-Centered Care Toolkit | <http://www.ihi.org/resources/Pages/Tools/StrategiesforLeadershipPatientandFamilyCenteredCareToolkit.aspx> |
| IHI | Care Management Institute at Kaiser Permanente | Toolkit: Getting Started in Video Ethnography — A Catalyst for Guiding and Motivating Quality Improvement | <http://www.ihi.org/resources/Pages/Tools/ToolkitGettingStartedVideoEthnography.aspx> |
| RWJF | RWJF | Engaging Patients in Improving Ambulatory Care: A Compendium of Tools from Maine, Oregon, and Humboldt County, California | <http://www.rwjf.org/en/research-publications/find-rwjf-research/2013/03/engaging-patients-in-improving-ambulatory-care.html> |
| RWJF | RWJF | Expecting Success Toolkit: Excellence in Cardiac Care | <http://www.rwjf.org/en/research-publications/find-rwjf-research/2008/06/expecting-success-toolkit.html> |
| RWJF | RWJF | Health Research & Educational Trust Disparities Toolkit | <http://www.rwjf.org/en/research-publications/find-rwjf-research/2007/01/health-research---educational-trust-disparities-toolkit.html> |
| RWJF | RWJF | More Than Words Toolkit Series | <http://www.hablamosjuntos.org/mtw/default.toolkit.asp> |
| RWJF | RWJF | PREPARE for Pandemic Influenza: A Quality Improvement Toolkit | <http://www.rwjf.org/en/research-publications/find-rwjf-research/2008/01/prepare-for-pandemic-influenza.html> |
| RWJF | RWJF | Speaking Together Toolkit | <http://www.rwjf.org/en/research-publications/find-rwjf-research/2008/06/speaking-together-toolkit0.html> |
| RWJF | RWJF | The National Health Plan Collaborative Toolkit: Toolkit to Reduce Racial & Ethnic Disparities in Health Care | <http://www.rwjf.org/en/research-publications/find-rwjf-research/2008/09/the-national-health-plan-collaborative-toolkit.html> |
| RWJF | RWJF | Transforming Care at the Bedside (TCAB) Toolkit | <http://www.rwjf.org/en/research-publications/find-rwjf-research/2008/06/the-transforming-care-at-the-bedside-tcab-toolkit.html> |
| RWJF | RWJF | Urgent Matters Program Toolkit: Proven Solutions to ED Crowding | <http://www.rwjf.org/en/research-publications/find-rwjf-research/2010/03/urgent-matters-program-toolkit.html> |
| AORN | AAAHC | AAAHC Institute Patient Safety Tool Kit: Ambulatory Surgery and Surgical/Procedural Checklists | <http://www.aaahc.org/Global/pdfs/AAAHC%20Institute%20content/Patient%20Safety%20Toolkits/PST_surgical%20checklists_FINAL.pdf> |
| AORN | AORN | Correct Site Surgery Tool Kit | <http://www.aorn.org/Clinical_Practice/ToolKits/Correct_Site_Surgery_Tool_Kit/Correct_Site_Surgery_Tool_Kit.aspx> |
| AORN | AORN | Environmental Cleaning Tool Kit | <http://www.aorn.org/Clinical_Practice/ToolKits/Environmental_Cleaning_Tool_Kit/Environmental_Cleaning_Tool_Kit.aspx> |
| AORN | AORN | Human Factors in Health Care Tool Kit | <http://www.aorn.org/Clinical_Practice/ToolKits/Human_Factors_In_Health_Care_ToolKit/Human_Factors_in_Health_Care_Tool_Kit.aspx> |
| AORN | AORN | Just Culture Tool Kit | <http://www.aorn.org/Clinical_Practice/ToolKits/Just_Culture_ToolKit/Just_Culture_Tool_Kit.aspx> |
| AORN | AORN | Management of Surgical Smoke Tool Kit | <http://www.aorn.org/Clinical_Practice/ToolKits/Surgical_Smoke_Evacuation_ToolKit/Management_of_Surgical_Smoke_Tool_Kit.aspx> |
| AORN | AORN | Medication Safety Tool Kit | <http://www.aorn.org/Clinical_Practice/ToolKits/Medication_Safety_Tool_Kit/Medication_Safety_Tool_Kit.aspx> |
| AORN | AORN | Patient Hand Off Tool Kit | <http://www.aorn.org/Clinical_Practice/ToolKits/Patient_Hand_Off_Tool_Kit/Patient_Hand_Off_Tool_Kit.aspx> |
| AORN | AORN | Perioperative Efficiency Tool Kit | <http://www.aorn.org/Clinical_Practice/ToolKits/Periop_Efficiency_Tool_Kit/Perioperative_Efficiency_Tool_Kit.aspx> |
| AORN | AORN | Reducing Radiological Exposure in the ASC | <http://www.aorn.org/Clinical_Practice/ToolKits/Reducing_Radiological_Exposure_in_the_ASC/Reducing_Radiological_Exposure_in_the_ASC.aspx> |
| AORN | AORN | Safe Patient Handling Tool Kit | <http://www.aorn.org/Clinical_Practice/ToolKits/Safe_Patient_Handling/Safe_Patient_Handling_Tool_Kit.aspx> |
| AORN | AORN | Sharps Safety Tool Kit | <http://www.aorn.org/Clinical_Practice/ToolKits/Sharps_Tool_Kit/Sharps_Safety_Tool_Kit.aspx> |
| AORN | AORN | Workplace Safety Tool Kit | <http://www.aorn.org/Clinical_Practice/ToolKits/Workplace_Safety/Workplace_Safety_Tool_Kit.aspx> |
| ECRI | ECRI | Quality Improvement Toolkit | <https://www.ecri.org/Forms/Pages/Quality_Week.aspx> |
| ECRI | ECRI | PSO Deep Dive™ on Health Information Technology | <https://eshop.ecri.org/p-140-pso-deep-dive-health-information-technology.aspx> |
| ECRI | ECRI | PSO Deep Dive™ on Medication Safety Events | <https://eshop.ecri.org/p-142-pso-deep-dive-medication-safety-events.aspx> |
| ECRI | ECRI | PSO Deep Dive™ on Laboratory-related Safety Events | <https://eshop.ecri.org/p-171-pso-deep-dive-laboratory-related-safety-events.aspx> |
| VA | VA | Co-Managed Care Toolkit | <http://www.ruralhealth.va.gov/resource-centers/central/comanagement-toolkit.asp> |
| VA | VA | HCRC Teaching Guide: Reducing Alcohol Use with Brief Intervention | <http://www.hepatitis.va.gov/products/brief-intervention-teaching-guide.asp#S8X> |
| VA | VA | Heart Failure Toolkit for Providers | <http://www.queri.research.va.gov/chf/products/hf_toolkit/> |
| VA | VA | HIV Oral Rapid Test Implementation Toolkit | <http://www.queri.research.va.gov/tools/hiv_oral_rapid/> |
| VA | VA | Medication Use Evaluation (MUE) Toolkit | <http://www.pbm.va.gov/PBM/vacenterformedicationsafety/tools/MUEToolkit.pdf> |
| VA | VA | National Center for Patient Safety Falls Toolkit | <http://www.patientsafety.va.gov/professionals/onthejob/falls.asp> |
| VA | VA | National Center for PTSD Community Provider Toolkit | <http://www.mentalhealth.va.gov/communityproviders/#sthash.BpNr5uCl.dpbs> |
| VA | VA | Pain as the 5Th Vital Sign Toolkit | <http://www.va.gov/PAINMANAGEMENT/docs/TOOLKIT.pdf> |
| VA | VA | Patient Care Sling Selection and Usage Toolkit | <http://www.visn8.va.gov/patientsafetycenter/safePtHandling/toolkitSlings.asp> |
| VA | VA | Safe Bariatric Patient Handling Toolkit | <http://www.visn8.va.gov/patientsafetycenter/safePtHandling/toolkitBariatrics.asp> |
| VA | VA | Stroke Quality Improvement Toolkit | <http://www.queri.research.va.gov/tools/stroke-quality/> |
| CMS | CMS | Strong Start for Mothers and Newborns Toolkit | <http://www.cms.gov/Outreach-and-Education/Outreach/Partnerships/StrongStartToolkit.html> |
| CDC | CDC | 2012 CRE Toolkit - Guidance for Control of Carbapenem-resistant Enterobacteriaceae (CRE) | <http://www.cdc.gov/hai/organisms/cre/cre-toolkit/> |
| CDC | CDC | Patient Notification Toolkit | <http://www.cdc.gov/injectionsafety/pntoolkit/> |
| CDC | CDC | CDC CFS (chronic fatigue syndrome) Toolkit | <http://stacks.cdc.gov/view/cdc/11683> |
| CDC | CDC | NHSN Validation Guidance and Toolkit; Validation for 2012 Central Line-associated Bloodstream Infection (CLABSI) in ICUs | <http://www.cdc.gov/cfs/toolkit/> |
| CDC | CDC | Vaccine Storage and Handling Toolkit | <http://www.cdc.gov/vaccines/recs/storage/toolkit/default.htm> |
| CDC | CDC | Living a Balanced Life with Diabetes: A Toolkit Addressing Psychosocial Issues for American Indian and Alaska Native Peoples | <http://www.cdc.gov/diabetes/ndep/living-a-balanced-life.htm> |
| CDC | CDC | Evaluation Toolkit:Evaluation Toolkit: Patient and Provider Perspectives about Routine HIV Screening in Health Care Settings | <http://www.cdc.gov/hiv/pdf/testing_resources_CDC_Evaluation_Toolkit_Routine_HIV_Screening.pdf> |
| CDC | CDC | FASD Prevention Tool Kit for Women's Health Care Providers | <http://www.cdc.gov/ncbddd/fasd/acog_toolkit.html> |
| CDC | CDC | Responding to Influenza: A Toolkit for Prenatal Care Providers | <http://www.cdc.gov/flu/pdf/freeresources/updated/2011_influenza_prenatal_toolkit.pdf> |

## Appendix C: Toolkits included in the review

| **ID** | **Category** | **Link to toolkit** | **Publisher** | **Toolkit** |
| --- | --- | --- | --- | --- |
| Jones, 2017 | Antimicrobial stewardship (AMS) | http://www.rcgp.org.uk/clinical-and-research/resources/toolkits/target- antibiotic-toolkit.aspx | Public Health England, Royal College of General Practitioners | TARGET (Treat Antibiotics Responsibly; Guidance, Education, Tools) Antibiotics Toolkit |
| Ashiru-Oredope, 2016 | Antimicrobial stewardship (AMS) | http://www.rcgp.org.uk/clinical-and-research/resources/toolkits/target- antibiotic-toolkit.aspx  https://www.gov.uk/government/ publications/antimicrobial -stewardship-start-smart-then-focus | Public Health England, Royal College of General Practitioners | Treat Antibiotics Responsibly, Guidance, Education, Tools (TARGET) (primary care) and Start Smart Then Focus (SSTF) (trusts) |
| Bender, 2011 | Asthma management | http://respiratorytoolkit.org/ | Colorado Asthma Toolkit Program (collaboration between National Jewish Health, the University of Colorado School of Medicine, and High Plains Research Network), funded by grants from Colorado Department of Public Health and Environment | Colorado Asthma Toolkit |
| Taylor, 2017 | Asthma management | https://asthma.carolinas healthcare.org/ToolKit | Carolinas HealthCare System | Shared Decision Making (SDM) Toolkit (asthma management) |
| Nicolaidis, 2016 | Autism communication | https://autismandhealth.org/ | Academic Autism Spectrum Partnership in Research and Education, funded by National Institute for Mental Health | Academic Autism Spectrum Partnership in Research and Education (AASPIRE) Healthcare toolkit |
| Chrisman, 2011 | Brain injury symptom management | https://www.cdc.gov/headsup/providers/ | Centers for Disease Control and Prevention | Heads Up: Brain Injury in Your Practice |
| Latsko, 2015 | Cancer care | http://www.aamds.org/treating-mds-toolkit | Aplastic Anemia and Myelodysplastic Syndromes International Foundation (AA&MDSIF) | Treating Myelodysplastic Syndrome (MDS) Toolkit |
| Gulati, 2015 | Cancer screening | http://www.cancerresearchuk.org/health-professional/awareness-and-prevention/ be-clear-on-cancer/skin-cancer-campaign | British Association of Dermatologists and Cancer Research UK | General Practitioner (GP) Skin Cancer Toolkit |
| Spruce, 2012 | Cancer screening | http://nevadacancercoalition.org/ cancer-resources/screening-treatment/ | Nevada Colon Cancer Partnership and American Cancer Society | Improving Colon Cancer Screening in Nevada with a Primary Care Toolkit |
| Adsett, 2014 | Cardiac care | http://www.heartonline.org.au/ | HEART Online, funding from Queensland Health's State-wide Cardiac Clinical Network and National Heart Foundation of Australia | HEART (Heart Education Assessment Rehabilitation Toolkit) Online |
| Callard, 2012 | Care quality | http://www.qualitasconsortium.com/ index.cfm/programs-services/15-steps /15-steps-for-clinic-and-outpatient-settings /the-15-steps-challenge-toolkit/ | NHS Institute for Innovation and Improvement | 15 Steps Challenge toolkit |
| Kemertzis, 2018 | Clinical decision making | https://download.lww.com/wolters kluwer_vitalstream_com/PermaLink/JPHO/ A/JPHO_2018_01_18_KEMERTZIS _JPHO-17-49R2_SDC1.pdf | Royal Children’s Hospital Fertility Preservation Taskforce | Fertility Preservation Toolkit |
| Pierce, 2016 | Critical care | http://sccmmedia.sccm.org/documents /LearnICU/CPP-Protocol-Toolkit-2014.pdf | Society of Critical Care Medicine | Critical Care Protocol Toolkit (CCPT) |
| Han, 2013 | Depression care | http://otgateway.com/articles/1 3macarthurtoolkit.pdf | MacArthur Foundation | MDPC (MacArthur Foundation Depression and Primary Care) Depression Toolkit |
| Gray, 2017 | Diabetes care | https://orders.diabetes.ca/products/ building-competency-in-diabetes-education -physical-activity-and-exercise? variant=1443667969 | Canadian Diabetes Association | Building Competency in Diabetes Education: Physical Activity and Exercise |
| Fowles, 2014 | Diabetes care | http://diabetescare.nshealth.ca/ guidelines-resources/professionals-and-patients/professionals/exercise | Canadian Diabetes Association | Diabetes Physical Activity and Exercise Toolkit |
| Chesis, 2015 | Elective delivery | https://www.cmqcc.org/resources-tool -kits/toolkits/early-elective-deliveries-toolkit | California Maternal Quality Care Collaborative (CMQCC), March of Dimes | Elimination of Non-medically Indicated (Elective) Deliveries Before 39 Weeks Gestational Age |
| Alidina, 2015 | Elective delivery | https://www.cmqcc.org/resources -tool-kits/toolkits/early-elective- deliveries-toolkit | California Maternal Quality Care Collaborative (CMQCC), March of Dimes | Elimination of Non-medically Indicated (Elective) Deliveries Before 39 Weeks Gestational Age |
| Wyte-Lake, 2016 | Emergency preparedness | https://www.publichealth.va.gov/ about/vemec/index.asp | Veterans Health Administration | Home-Based Primary Care/Home Health Agency Disaster Preparedness Toolkit |
| Clancy, 2012 | Emergency preparedness | http://www.health.ny.gov/facilities/ hospital/emergency_preparedness /guideline_for_hospitals/docs/ emergency_preparedness_manual.pdf | New York State Department of Health | NY State Department of Health Pediatric and Obstetric Emergency Preparedness Toolkit |
| Henry, 2012 | Emergency/surgery capacity | http://www.who.int/surgery/publications /imeesc/en/ | World Health Organization | Integrated Management of Emergency and Essential Surgical Care (IMEESC) toolkit |
| Cox, 2017 | End-of-life care | https://www.surrey.ac.uk/sites/default /files/End%20of%20life %20care%20toolkit_0.pdf | University of Surrey, Ashford and St. Peter's Hospitals NHS Trust | End of Life Care Toolkit (part of Care Homes and hOspitals Innovating Collaboratively to increase End of life care options [CHOICE] Project) |
| Carroll, 2012 | Fall prevention | http://www.brighamandwomens.org /Medical_Professionals/nursing/ nursinged/Falls2Trial.aspx | Partners Healthcare | Fall TIPS (Tailoring Interventions for Patient Safety) |
| Dykes, 2017 | Fall prevention | http://www.brighamandwomens.org /Medical_Professionals/nursing/nursinged /Falls2Trial.aspx | Partners Healthcare | Fall TIPS (Tailoring Interventions for Patient Safety) Toolkit |
| Fisher, 2013 | Fall prevention | https://www.hospiceuk.org/what-we- offer/clinical-and-care-support /clinical-resources | Help the Hospices | Falls prevention and management toolkit |
| Stalhandske, 2008 | Fall prevention | http://www.patientsafety.va.gov/ professionals/onthejob/falls.asp | Veterans Health Administration | National Falls Toolkit |
| Coe, 2017 | Fall prevention | https://www.cdc.gov/steadi/index.html | Centers for Disease Control and Prevention | Stopping Elderly Accidents, Deaths, and Injuries |
| Ryan, 2013 | Geriatric care | http://giic.rgps.on.ca/toolkit-libraries | Regional Geriatric Programs of Ontario | Geriatrics, Interprofessional Practice, and Interorganizational Collaboration (GiiC) Toolkit |
| Mabachi, 2016 | Health literacy | https://www.ahrq.gov/professionals/ quality-patient-safety/quality-resources /tools/literacy-toolkit/index.html | Agency for Healthcare Research and Quality | Health Literacy Universal Precautions (HLUP) Toolkit |
| Dore, 2013 | Health literacy | http://www.med.unc.edu/tarc/files/ HLUPTRheum.pdf | Agency for Healthcare Research and Quality | Health Literacy Universal Precautions Toolkit for Rheumatology (HLUPTK-R) |
| Koelling, 2006 | Heart failure | http://www.onlinejacc.org/content/ vol46/10_Suppl_B/ | University of Michigan Health System | Guidelines Applied in Practice - Heart Failure (GAP-HF) Tool Kit |
| Perumalswami, 2016 | Hepatitis C care | http://hepcure.org/ | Mount Sinai Medical Center | HepCure (Hepatitis C education and patient engagement) |
| Adams, 2014 | Hospital readmission | http://www.ahrq.gov/professionals/ systems/hospital/red/toolkit/index.html | Agency for Healthcare Research and Quality | Project Re-Engineered Discharge (Project RED) Toolkit |
| Mitchell, 20015 | Hospital readmission | https://www.ahrq.gov/professionals/ systems/hospital/red/toolkit/index.html | Agency for Healthcare Research and Quality | Project Re-Engineered Discharge (Project RED) Toolkit |
| Enfield, 2014 | Hospital-acquired infections | https://www.cdc.gov/hai/pdfs/ cre/cre-guidance-508.pdf | Centers for Disease Control and Prevention | Center for Disease Control and Prevention's Carbapenem-resistant Enterobacteriaceae (CRE) Toolkit |
| Randle, 2006 | Hospital-acquired infections | http://www.who.int/gpsc/ 5may_advocacy-toolkit.pdf?ua=1 | National Patient Safety Agency (UK) | Clean-Your-Hands Campaign Toolkit |
| Septimus, 2016 | Hospital-acquired infections | https://www.ahrq.gov/professionals /systems/hospital/universal_icu _decolonization/index.html | Agency for Healthcare Research and Quality | Universal ICU Decolonization Toolkit: An Enhanced Protocol |
| Haley 2015 | Kidney disease care | http://www.renalmd.org/default.asp? page=CKDToolkit | Renal Physicians Association | Advanced Chronic Kidney Disease (CKD) Patient Management Toolkit |
| Fernald, 2015 | Medical errors | https://ftp.cdc.gov/pub/cliac_meeting_ presentations/pdf/addenda/cliac0314/ 16a_west_cdclabtoolkit2013_handout.pdf | Shared Networks of Colorado Ambulatory Practices and Partners (SNOCAP) | Quality Improvement for Laboratory Testing Processes in Primary Care: Implementation Guide and Toolkit |
| Leape, 2006 | Medical errors | http://www.macoalition.org/reducing_ medication_errors.shtml and http://www.macoalition.org/Initiatives/ CCTRToolkit.shtml | Massachusetts Coalition for the Prevention of Medical Errors | Reconciling Medications (RM) Toolkit and Communicating Critical Test Results (CCTR) Toolkit |
| Mueller, 2013 | Medication management | http://www.hospitalmedicine.org/ about_shm/webformz/form_wfz _imptk_marquis.aspx?iFormSubmission Key=1cf0b471-ecec-49ba-b702-abbf8083ecab | Society of Hospital Medicine, as part of the Multi-Center Medication Reconciliation Quality Improvement Study (MARQUIS) | Medication Reconciliation Implementation toolkit |
| McHugo, 2007 | Mental health | http://store.samhsa.gov/list/series? name=Evidence-Based-Practices-KITs | Center for Mental Health Services of SAMHSA | Evidence-Based Practices Implementation Resource Kits |
| MacDonald-Wilson, 2017 | Mental health decision support | https://store.patdeegan.com/products/ decision-support-toolkit | P.E. Deegan and Associates | Toolkit: Decision Support |
| Miller, 2014 | Multiple sclerosis symptom management | http://www.achlpicme.org/MS/CMEInfo.aspx | Academy for Continued Healthcare Learning | Toolkit: Constructing an Adaptive Care Model for the Management of Disease-Related Symptoms Throughout the Course of Multiple Sclerosis |
| Guillory, 2017 | Newborn screening | https://www.dshs.texas.gov/ newborn/cchdtoolkit/ | Texas Department of State Health and Human Services | Critical Congenital Heart Disease (CCHD) Toolkit |
| Dobbins, 2005 | Nursing best practices | http://rnao.ca/bpg/resources/ toolkit-implementation-best-practice -guidelines-second-edition | Registered Nurses Association of Ontario | Toolkit: Implementation of Best Practice Guidelines |
| Main, 2017 | Obstetric care | https://www.cmqcc.org/resources -tool-kits/toolkits/ob-hemorrhage-toolkit | California Maternal Quality Care Collaborative | California Toolkit to Transform Maternal Care: Improving Health Care Response to Obstetric Hemorrhage Version 2.0 |
| Pulver, 2012 | Pain management | http://www.nps.org.au/health-professionals/cpd/activities/due-for- hospitals/acute-postoperative-pain/apop | National Prescribing Service (Australia) | Acute Postoperative Pain Management (APOP) Toolkit |
| Fine, 2014 | Pain management | http://achlpicme.org/ltc/CMEInfo.aspx | Academy for Continued Healthcare Learning | Toolkit: Enhancing the Management of Neuropathic Pain in the Long-term Care Setting |
| Kuhlman, 2014 | Parental education | http://www.kidsks.org/safe-sleep-toolkit.html | Kansas Infant Death and SIDS (KIDS) Network | Safe Sleep Toolkit |
| Schauberger, 2006 | Patient safety | https://www.healthpartners.com/ ucm/groups/public/@hp/@ public/documents/documents/ vgn_pdf_56420.pdf | Gunderson Lutheran | Ambulatory Patient Safety Toolkit |
| Parkman, 2013 | Patient safety | http://health.mil/Military-Health-Topics/Access-Cost-Quality-and-Safety/Quality-And-Safety-of-Healthcare/Patient-Safety/Patient-Safety-Products-And-Services/Toolkits | Department of Defense | Patient safety |
| Thomason, 2016 | Patient Safety | https://www.queri.research. va.gov/tools/sci-pumt/ | Veterans Health Administration | Spinal Cord Industry Pressure Ulcer Monitoring Tool (SCI-PUMT) Toolkit |
| Lannon, 2008 | Pediatric preventive care | https://brightfutures.aap.org/materials-and-tools/Pages/default.aspx | American Association of Pediatrics | Bright Futures Training Intervention Project toolkit |
| Byrne, 2011 | Perinatal care | https://www.cpqcc.org/qi-tool-kits/antenatal-corticosteroid-therapy | California Perinatal Quality Care Collaborative | California Perinatal Quality Care Collaborative Antenatal Corticosteroid Therapy (ANS) Toolkit |
| Lyndon, 2016 | Perinatal care | https://www.cmqcc.org/resources-tool-kits/toolkits/ob-hemorrhage-toolkit | California Maternal Quality Care Collaborative | Improving Health Care Response to Obstetric Hemorrhage Version 2.0 A California Quality Improvement Toolkit ("Obstetric Hemorrhage Toolkit") |
| Kohler, 2015 | Perinatal care | https://www.cmqcc.org/resources-tool-kits/toolkits/preeclampsia-toolkit | California Maternal Quality Care Collaborative | Improving Health Care Response to Preeclampsia |
| Ezzat, 2017 | Physical therapy | http://physicaltherapy.med.ubc.ca/physical-therapy-knowledge-broker/tendinopathy-toolkit/ | Physiotherapy Association of British Columbia (PABC) | Achilles Tendinopathy Toolkit (ATT) |
| Brown, 2015 | Psychotherapy decision support | https://www.psychoutcomes.org/ DecisionSupportToolkit/WebHome | A Collaborative Outcomes Resource Network (ACORN) | A Collaborative Outcomes Resource Network (ACORN) Clinical Decision Support Toolkit |
| Sopcak, 2016 | Screening | http://www.better-program.ca/resources/ | The Better Project, University of Toronto Family and Community Medicine, University of Alberta, Health Informatics Institute | Building on Existing Tools to Improve Chronic Disease Prevention and Screening in Primary Care (BETTER) |
| Shellhaas, 2016 | Screening, quality improvement | http://ohiogdm.com/Providers/Clinical-Tools | Ohio Gestational Diabetes Postpartum Care Learning Collaborative | Gestational Diabetes Mellitus (GDM) Toolkit |
| Shershneva, 2010 | Smoking cessation | https://register.cmeenterprise.com/8529/ | CS2day initiative, Pfizer | CS2day Toolkit |
| Sarna, 2017 | Smoking cessation | https://tobaccofreenurses.org/projects/rnql-hsq/louisiana | Tobacco Free Nurses, Pfizer Independent Grants for Learning & Change, Smoking Cessation Leadership Center | Registered Nurses Referral to Quitlines - Helping Smokers Quit Louisiana Toolkit |
| Pratt, 2012 | Staff trauma support | http://www.mitsstools.org/store/ p2/Clinician_Support_Tool_Kit_ for_Healthcare.html | Medically Induced Trauma Support Services | Clinician Support Tool Kit for Healthcare |
| Brooks, 2013 | Substance abuse | http://www.tresearch.org/tools/for-clinicians/roadmap-toolkit/ | Treatment Research Institute | RoadMAP Relapse Prevention Group Counseling Toolkit |
| Levy, 2017 | Substance use screening | http://massclearinghouse. ehs.state.ma.us/BSASSBIRTPROG /SA1099.html | Commonwealth of Massachusetts Department of Public Health Bureau of Substance Abuse Services, Massachusetts Child Psychiatry Access Project, Massachusetts Department of Mental Health, Boston Children's Hospital Adolescent Substance Abuse Program | Adolescent Screening, Brief Intervention, and Referral for Treatment for Alcohol and Other Drug Use Toolkit for Providers |
| Nowalk, 2014 | Vaccination | http://4pillarstoolkit.pitt.edu/ | University of Pittsburgh | 4 Pillars Toolkit |
| Zimmerman, 2014 | Vaccination | http://4pillarstoolkit.pitt.edu/ | University of Pittsburgh | 4 Pillars Toolkit for Increasing Childhood Influenza Immunization |
| Nace, 2011 | Vaccination | http://www.paltc.org/product-store /immunizations-long-term-care-setting | American Medical Directors Association | American Medical Directors Association Immunization Toolkit |
| Gibson, 2016 | Weight management | https://mainehealth.org/lets-go/childrens-program/pediatric-family-practices/tools | MaineHealth | 5210 Let's Go! Childhood Obesity Resource Toolkit for Healthcare Professionals |
| Rueda-Clausen, 2014 | Weight management | http://www.obesitynetwork.ca/5As | Canadian Obesity Network | 5As of Obesity Management for Adults |
| Smith, 2011 | Weight management | http://www.aafp.org/dam/AAFP/ documents/patient_care/fitness/ AIMBrochure.pdf | American Academy of Family Physicians | Americans In Motion (AIM) to Change Toolkit |
| Abraham, 2007 | Weight management | http://www.americaonthemove.org/ | America-On-The-Move | America-on-the-Move Toolkit |
| Kinsinger, 2009 | Weight management | http://www.move.va.gov/ referencetools.asp | Veterans Health Administration | MOVE (Managing Overweight/Obesity for Veterans Everywhere) Weight Management Program |
| Sample, 2013 | Weight management | http://www.eatsmartmovemorenc.  com/PediatricObesityTools/Pediatric  ObesityTools.html | Healthy Carolinians of Orange County, Orange County Health Department, NC | Pediatric Obesity Toolkit |
| Stiff, 2014 | Weight management | http://healthvermont.gov/family  /fit/documents/Promoting_Healthier_Weight_toolkit.pdf | Vermont Area Health Education Centers (AHEC) Network and Vermont Department of Health | Promoting Healthier Weight in Adult Primary Care |
| Mulloy, 2008 | Wrong site surgery | https://www.aorn.org/aorn-org/guidelines/clinical-resources/tool-kits/correct-site-surgery-tool-kit | Association of periOperative Registered Nurses | AORN Correct Site Surgery Tool Kit |

## Appendix D: Critical Appraisal QI-MQCS

| **Author, Year** | **Organizational Motivation** | **Intervention Rationale** | **Intervention Description** | **Organizational Characteristics** | **Implementation** | **Study Design** | **Comparator** | **Data Source** | **Timing** | **Adherence / Fidelity** | **Health Outcomes** | **Organizational Readiness** | **Penetration / Reach** | **Sustainability** | **Spread** | **Limitations** |
| --- | --- | --- | --- | --- | --- | --- | --- | --- | --- | --- | --- | --- | --- | --- | --- | --- |
| Abraham, 2007 [[1](#_ENREF_1)] | Not met | Not met | Met | Met | Met | Not met | Not met | Met | Met | Not met | Not met | Met | Not met | Not met | Met | Not met |
| Adams, 2014 [[2](#_ENREF_2)] | Met | Met | Met | Met | Met | Not met | Met | Met | Not met | Met | Not met | Met | Not met | Not met | Met | Met |
| Adsett, 2014 [[3](#_ENREF_3)] | Met | Met | Met | Not met | Not met | Not met | Not met | Met | Not met | Not met | Not met | Not Met | Not met | Not met | Met | Not met |
| Alidina, 2015 [[4](#_ENREF_4)] | Met | Not met | Not met | Not met | Not met | Met | Not met | Not met | Not met | Not met | Not met | Not Met | Not met | Not met | Met | Not met |
| Ashiru-Oredope, 2016 [[5](#_ENREF_5)] | Met | Met | Met | Met | Not met | Not met | Not met | Met | Not met | Met | Not met | Not Met | Met | Not met | Met | Met |
| Bender, 2011 [[6](#_ENREF_6)] | Met | Met | Met | Met | Met | Met | Met | Met | Not met | Met | Not met | Met | Met | Met | Met | Not met |
| Brooks, 2013 [[7](#_ENREF_7)] | Met | Met | Met | Met | Met | Met | Not met | Met | Met | Met | Not met | Not Met | Met | Met | Met | Met |
| Brown, 2015 [[8](#_ENREF_8)] | Met | Met | Not met | Not met | Not met | Not met | Not met | Met | Not met | Met | Met | Not Met | Not met | Not met | Met | Not met |
| Byrne, 2011 [[9](#_ENREF_9)] | Met | Met | Not met | Met | Not met | Not met | Not met | Not met | Met | Not met | Not met | Not Met | Not met | Not met | Met | Not met |
| Callard, 2012 [[10](#_ENREF_10)] | Met | Met | Met | Not met | Not met | Not met | Not met | Not met | Not met | Not met | Not met | Met | Not met | Not met | Met | Not met |
| Carroll, 2012 [[11](#_ENREF_11)] | Met | Met | Met | Met | Met | Not met | Met | Met | Met | Met | Not met | Not Met | Not met | Not met | Met | Met |
| Chesis, 2015 [[12](#_ENREF_12)] | Not met | Not met | Not met | Not met | Met | Not met | Not met | Not met | Met | Not met | Met | Not Met | Not met | Met | Met | Not met |
| Chrisman, 2011 [[13](#_ENREF_13)] | Met | Met | Not met | Met | Met | Met | Not met | Met | Not met | Not met | Not met | Not Met | Not met | Not met | Met | Met |
| Clancy, 2012 [[14](#_ENREF_14)] | Met | Met | Met | Met | Met | Not met | Met | Met | Met | Met | Not met | Met | Met | Met | Met | Met |
| Coe, 2017 [[15](#_ENREF_15)] | Met | Met | Met | Met | Met | Not met | Not met | Met | Met | Not met | Not met | Met | Met | Met | Met | Not met |
| Cox, 2017 [[16](#_ENREF_16)] | Met | Met | Met | Met | Met | Met | Not met | Met | Met | Not met | Not met | Met | Met | Met | Met | Met |
| Dobbins, 2005 [[17](#_ENREF_17)] | Met | Not met | Not met | Not met | Met | Not met | Not met | Met | Not met | Met | Not met | Met | Not met | Not met | Met | Met |
| Dore, 2013 [[18](#_ENREF_18)] | Met | Met | Met | Not met | Met | Not met | Not met | Met | Met | Met | Not met | Met | Not met | Not met | Met | Not met |
| Dykes, 2009 [[19](#_ENREF_19)] | Met | Met | Met | Met | Met | Not met | Met | Met | Not met | Met | Not met | Met | Not met | Met | Met | Met |
| Enfield, 2014 [[20](#_ENREF_20)] | Met | Met | Met | Met | Met | Met | Met | Met | Met | Met | Met | Not Met | Not met | Met | Met | Met |
| Ezzat, 2017 [[21](#_ENREF_21)] | Met | Met | Not met | Met | Met | Not met | Not met | Met | Not met | Met | Not met | Met | Met | Not met | Met | Met |
| Fernald, 2015 [[22](#_ENREF_22)] | Met | Met | Met | Met | Met | Not met | Not met | Met | Not met | Met | Not met | Met | Met | Met | Met | Met |
| Fine, 2014 [[23](#_ENREF_23)] | Met | Met | Met | Met | Met | Met | Not met | Met | Met | Not met | Not met | Not Met | Not met | Not met | Met | Met |
| Fisher, 2013 [[24](#_ENREF_24)] | Met | Met | Not met | Not met | Met | Not met | Met | Met | Met | Not met | Met | Met | Met | Met | Met | Met |
| Fowles, 2014 [[25](#_ENREF_25)] | Met | Met | Met | Met | Met | Met | Met | Met | Met | Not met | Met | Met | Not met | Not met | Met | Met |
| Gibson, 2016 [[26](#_ENREF_26)] | Met | Met | Met | Met | Not met | Not met | Not met | Met | Not met | Met | Not met | Met | Not met | Met | Met | Met |
| Gray, 2017 [[27](#_ENREF_27)] | Met | Met | Met | Met | Met | Not met | Met | Met | Met | Met | Not met | Met | Not met | Not met | Met | Met |
| Guillory, 2017 [[28](#_ENREF_28)] | Met | Met | Met | Met | Met | Met | Met | Met | Met | Met | Not met | Not Met | Met | Met | Met | Met |
| Gulati, 2015 [[29](#_ENREF_29)] | Met | Met | Met | Met | Met | Not met | Not met | Met | Met | Met | Not met | Not Met | Met | Not met | Met | Met |
| Haley, 2015 [[30](#_ENREF_30)] | Met | Met | Met | Met | Met | Met | Met | Met | Not met | Met | Not met | Met | Not met | Met | Met | Met |
| Han, 2013 [[31](#_ENREF_31)] | Met | Met | Met | Met | Not met | Not met | Not met | Met | Not met | Met | Not met | Not Met | Not met | Not met | Met | Met |
| Henry, 2012 [[32](#_ENREF_32)] | Met | Met | Met | Met | Met | Not met | Met | Met | Met | Not met | Not met | Met | Met | Met | Met | Met |
| Kinsinger, 2009 [[33](#_ENREF_33)] | Met | Met | Met | Not met | Met | Met | Not met | Met | Met | Met | Not met | Not Met | Met | Not met | Met | Met |
| Koelling, 2006 [[34](#_ENREF_34)] | Not met | Met | Met | Met | Met | Not met | Not met | Not met | Not met | Not met | Met | Met | Not met | Not met | Met | Not met |
| Kuhlmann, 2014 [[35](#_ENREF_35)] | Met | Met | Met | Not met | Not met | Not met | Not met | Not met | Not met | Not met | Not met | Not Met | Not met | Not met | Met | Met |
| Lannon, 2008 [[36](#_ENREF_36)] | Met | Met | Met | Met | Met | Met | Not met | Met | Met | Met | Not met | Met | Not met | Not met | Met | Met |
| Latsko, 2015 [[37](#_ENREF_37)] | Met | Not met | Met | Not met | Not met | Not met | Not met | Met | Not met | Not met | Not met | Not Met | Not met | Not met | Met | Not met |
| Leape, 2006 [[38](#_ENREF_38)] | Met | Met | Met | Met | Met | Not met | Not met | Not met | Not met | Met | Not met | Met | Met | Met | Met | Not met |
| Levy, 2017 [[39](#_ENREF_39)] | Met | Met | Met | Met | Met | Not met | Met | Met | Not met | Met | Not met | Met | Not met | Not met | Met | Met |
| Lyndon, 2016 [[40](#_ENREF_40)] | Met | Not met | Met | Not met | Met | Met | Not met | Not met | Not met | Met | Not met | Met | Not met | Met | Met | Met |
| Mabachi, 2016, [[41](#_ENREF_41)] | Met | Met | Not met | Met | Met | Not met | Not met | Met | Met | Not met | Not met | Met | Not met | Met | Met | Met |
| MacDonald-Wilson, 2017 [[42](#_ENREF_42)] | Met | Met | Not met | Met | Met | Not met | Not met | Met | Not met | Met | Not met | Met | Not met | Met | Met | Met |
| Main, 2017 [[43](#_ENREF_43)] | Met | Met | Not met | Met | Met | Met | Not met | Met | Met | Met | Met | Not Met | Met | Met | Met | Met |
| McHugo, 2007 [[44](#_ENREF_44)] | Met | Met | Not met | Met | Met | Met | Not met | Met | Met | Met | Not met | Met | Not met | Met | Met | Met |
| Miller, 2014 [[45](#_ENREF_45)] | Met | Met | Met | Met | Met | Not met | Not met | Met | Met | Not met | Not met | Met | Not met | Not met | Met | Not met |
| Mitchell, 2016 [[46](#_ENREF_46)] | Met | Met | Met | Met | Met | Met | Not met | Met | Met | Met | Met | Met | Not met | Met | Met | Met |
| Mueller, 2013 [[47](#_ENREF_47)] | Met | Met | Met | Met | Met | Not met | Not met | Not met | Not met | Not met | Not met | Met | Not met | Not met | Met | Met |
| Mulloy, 2008 [[48](#_ENREF_48)] | Met | Met | Met | Met | Met | Not met | Not met | Met | Met | Met | Met | Met | Not met | Met | Met | Met |
| Nace, 2011 [[49](#_ENREF_49)] | Met | Met | Not met | Met | Met | Not met | Not met | Not met | Met | Not met | Not met | Met | Not met | Met | Met | Met |
| Nicolaidis, 2016 [[50](#_ENREF_50)] | Not met | Met | Met | Not met | Not met | Met | Not met | Met | Met | Not met | Not met | Met | Not met | Not met | Met | Met |
| Nowalk, 2014 [[51](#_ENREF_51)] | Met | Met | Met | Met | Met | Met | Not met | Met | Met | Met | Not met | Met | Not met | Not met | Met | Met |
| Parkman, 2013 [[52](#_ENREF_52)] | Met | Met | Not met | Met | Met | Met | Not met | Met | Met | Met | Not met | Not Met | Not met | Not met | Met | Met |
| Perumalswami, 2016 [[53](#_ENREF_53)] | Met | Met | Met | Not met | Met | Not met | Not met | Not met | Not met | Not met | Not met | Met | Not met | Not met | Met | Not met |
| Pierce, 2016 [[54](#_ENREF_54)] | Met | Met | Not met | Not met | Not met | Met | Met | Met | Not met | Met | Not met | Not Met | Not met | Not met | Met | Not met |
| Pratt, 2012 [[55](#_ENREF_55)] | Met | Met | Not met | Not met | Met | Not met | Met | Met | Not met | Not met | Not met | Not Met | Not met | Not met | Met | Not met |
| Pulver, 2012 [[56](#_ENREF_56)] | Met | Met | Met | Not met | Met | Not met | Met | Met | Met | Met | Not met | Met | Not met | Met | Met | Not met |
| Randle, 2006 [[57](#_ENREF_57)] | Met | Met | Met | Met | Met | Not met | Not met | Met | Met | Met | Not met | Met | Not met | Met | Met | Met |
| Rueda-Clausen, 2014 [[58](#_ENREF_58)] | Met | Met | Met | Met | Met | Met | Not met | Met | Met | Met | Not met | Not Met | Met | Not met | Met | Met |
| Ryan, 2013 [[59](#_ENREF_59)] | Met | Met | Met | Met | Met | Not met | Not met | Not met | Met | Met | Not met | Met | Met | Met | Met | Met |
| Sample, 2013 [[60](#_ENREF_60)] | Met | Met | Met | Not met | Met | Not met | Not met | Met | Met | Met | Not met | Met | Not met | Not met | Met | Not met |
| Sarna, 2017 [[61](#_ENREF_61)] | Met | Met | Not met | Met | Met | Met | Met | Met | Met | Met | Not met | Not Met | Not met | Met | Met | Met |
| Schauberger, 2006 [[62](#_ENREF_62)] | Met | Met | Met | Not met | Met | Not met | Not met | Not met | Met | Not met | Not met | Met | Not met | Met | Met | Met |
| Septimus, 2016 [[63](#_ENREF_63)] | Met | Met | Not met | Met | Met | Met | Not met | Met | Met | Met | Met | Met | Met | Not met | Met | Met |
| Shellhaus, 2016 [[64](#_ENREF_64)] | Met | Met | Met | Met | Met | Not met | Not met | Met | Met | Not met | Not met | Met | Not met | Not met | Met | Met |
| Shershneva, 2010 [[65](#_ENREF_65)] | Met | Met | Met | Not met | Not met | Not met | Not met | Met | Not met | Not met | Not met | Not Met | Not met | Not met | Met | Not met |
| Smith, 2010 [[66](#_ENREF_66)] | Met | Met | Met | Met | Met | Met | Not met | Met | Met | Met | Not met | Not Met | Not met | Not met | Met | Met |
| Sopcak, 2016 [[67](#_ENREF_67)] | Met | Met | Met | Met | Met | Not met | Not met | Met | Met | Not met | Not met | Met | Not met | Met | Met | Met |
| Spruce, 2012 [[68](#_ENREF_68)] | Met | Met | Met | Not met | Met | Not met | Not met | Met | Not met | Not met | Not met | Not Met | Not met | Not met | Met | Not met |
| Stalhandske, 2008 [[69](#_ENREF_69)] | Met | Not met | Not met | Not met | Not met | Not met | Not met | Met | Not met | Not met | Met | Not Met | Not met | Not met | Met | Not met |
| Stiff, 2014 [[70](#_ENREF_70)] | Met | Met | Met | Met | Not met | Met | Not met | Met | Not met | Met | Not met | Not Met | Not met | Not met | Met | Not met |
| Taylor, 2017 [[71](#_ENREF_71)] | Met | Met | Met | Met | Met | Not met | Not met | Met | Met | Not met | Met | Met | Met | Not met | Met | Met |
| Thomason, 2016 [[72](#_ENREF_72)] | Met | Met | Not met | Met | Met | Met | Met | Met | Met | Met | Not met | Met | Met | Met | Met | Met |
| von Kohler, 2015 [[73](#_ENREF_73)] | Not met | Not met | Not met | Not met | Met | Not met | Not met | Not met | Not met | Not met | Not met | Met | Not met | Not met | Met | Not met |
| Wyte-Lake, 2017 [[74](#_ENREF_74)] | Met | Met | Not met | Met | Met | Not met | Not met | Met | Met | Not met | Not met | Met | Met | Not met | Met | Met |
| Zimmerman, 2014 [[75](#_ENREF_75)] | Met | Met | Met | Met | Met | Met | Met | Met | Met | Met | Not met | Met | Not met | Not met | Met | Met |

Notes:

Met: QI-MQCS criterion met, see text

QI-MQCS Domains:

Organizational motivation: Organizational problem, reason or motivation for the intervention stated

Intervention rationale: Rationale provided linking the intervention to its expected effects

Intervention description: Change in organizational or provider behavior described [toolkit content]

Organizational characteristics: Demographics or basic characteristics of the organization provided

Implementation: Temporary activities used to introduce potentially enduring changes described

Study design: Study design and comparator clearly stated

Comparator: Information about comparator care processes provided

Data source: Data sources and outcome definition documented

Timing: Timing of intervention and evaluation clearly outlined

Adherence/fidelity: Adherence to the intervention

Health outcomes: Patient health-related outcomes addressed

Organizational readiness: Barriers and facilitators to readiness in the organization described

Penetration/reach: Penetration/reach of the intervention stated

Sustainability: Sustainability of the intervention addressed

Spread: Ability to be spread or replicated communicated [use of toolkit as a tool for spread]

Limitations: Interpretation of the evaluation discussed

References

1. Abraham A, Stuht J, Emsermann CB, Kutner JS (2007) Impact of distribution of the America-on-the-move toolkit on primary care providers' self-reported exercise and dietary counseling with overweight patients. Journal of General Internal Medicine 22: 105-105.

2. Adams CJ, Stephens K, Whiteman K, Kersteen H, Katruska J (2014) Implementation of the Re-Engineered Discharge (RED) Toolkit to Decrease All-Cause Readmission Rates at a Rural Community Hospital. Qual Manag Health Care 23: 169-177.

3. Adsett JA, Mullins R, Page K, Hickey A (2014) Heart Education Assessment and Rehabilitation Toolkit: HEART Online. Translating research into practice. European Journal of Heart Failure 16: 62-63.

4. Alidina JR, Prieto J, Cole C, Ramer K, Chez-Flood B (2015) Decreasing Early Term Elective Deliveries: One Hospital's Implementation of the 39 Week Toolkit and Quality Improvement Measurements. Reproductive Sciences 22: 261A-261A.

5. Ashiru-Oredope D, Budd EL, Bhattacharya A, Din N, McNulty CAM, et al. (2016) Implementation of antimicrobial stewardship interventions recommended by national toolkits in primary and secondary healthcare sectors in England: TARGET and Start Smart Then Focus. Journal of Antimicrobial Chemotherapy 71: 1408-1414.

6. Bender BG, Dickinson P, Rankin A, Wamboldt FS, Zittleman L, et al. (2011) The Colorado Asthma Toolkit Program: a practice coaching intervention from the High Plains Research Network. J Am Board Fam Med 24: 240-248.

7. Brooks AC, Carpenedo CM, Fairfax-Columbo J, Clements NT, Benishek LA, et al. (2013) The RoadMAP Relapse Prevention Group Counseling Toolkit: counselor adherence and competence outcomes. J Subst Abuse Treat 45: 356-362.

8. Brown GS, Simon A, Cameron J, Minami T (2015) A collaborative outcome resource network (ACORN): Tools for increasing the value of psychotherapy. Psychotherapy (Chic) 52: 412-421.

9. Byrne J, Govindaswami B, Jegatheesan P, Jelks A, Kunz L, et al. (2011) Perinatal core measure: antenatal steroid performance improvement following a preterm birth risk assessment decision model and perinatal QI toolkit. American Journal of Obstetrics and Gynecology 204: S193-S193.

10. Callard L, Williams A (2012) The 15 steps challenge: a toolkit for good care. Nurs Manag (Harrow) 19: 14-18.

11. Carroll DL, Dykes PC, Hurley AC (2012) An electronic fall prevention toolkit: effect on documentation quality. Nurs Res 61: 309-313.

12. Chesis N (2015) A Quality Improvement Project to Reduce the Incidence of Nonmedically Indicated Elective Deliveries Before 39 Weeks...Proceedings of the 2015 AWHONN Convention. JOGNN: Journal of Obstetric, Gynecologic & Neonatal Nursing 44: S49-50.

13. Chrisman SP, Schiff MA, Rivara FP (2011) Physician concussion knowledge and the effect of mailing the CDC's "Heads Up" toolkit. Clin Pediatr (Phila) 50: 1031-1039.

14. Clancy KA, Kacica MA (2012) Ready for our children? Results from a survey of upstate New York hospitals' utilization of Pediatric Emergency Preparedness Toolkit guidance. Disaster Med Public Health Prep 6: 138-145.

15. Coe LJ, St John JA, Hariprasad S, Shankar KN, MacCulloch PA, et al. (2017) An Integrated Approach to Falls Prevention: A Model for Linking Clinical and Community Interventions through the Massachusetts Prevention and Wellness Trust Fund. Front Public Health 5: 38.

16. Cox A, Arber A, Bailey F, Dargan S, Gannon C, et al. (2017) Developing, implementing and evaluating an end of life care intervention. Nurs Older People 29: 27-35.

17. Dobbins M, Davies B, Danseco E, Edwards N, Virani T (2005) Changing nursing practice: evaluating the usefulness of a best-practice guideline implementation toolkit. Nurs Leadersh (Tor Ont) 18: 34-45.

18. Dore A, Dye J, Hourani L, Hackney B, Criscione-Schreiber LG, et al. (2013) Incorporating The Health Literacy Universal Precautions Toolkit Quick Start In Academic Rheumatology Practices: Carolina Fellows Collaborative. Arthritis and Rheumatism 65: S414-S414.

19. Dykes PC, Carroll DL, Hurley A, Gersh-Zaremski R, Kennedy A, et al. (2009) Fall TIPS: strategies to promote adoption and use of a fall prevention toolkit. AMIA Annu Symp Proc 2009: 153-157.

20. Enfield KB, Huq NN, Gosseling MF, Low DJ, Hazen KC, et al. (2014) Control of Simultaneous Outbreaks of Carbapenemase-Producing Enterobacteriaceae and Extensively Drug-Resistant Acinetobacter baumannii Infection in an Intensive Care Unit Using Interventions Promoted in the Centers for Disease Control and Prevention 2012 Carbapenemase-Resistant Enterobacteriaceae Toolkit. Infection Control and Hospital Epidemiology 35: 810-817.

21. Ezzat AM, Schneeberg A, Huisman ES, White LD, Kennedy C, et al. (2017) A cross-sectional evaluation examining the use of the Achilles tendinopathy toolkit by physiotherapists in British Columbia, Canada. Disabil Rehabil 39: 671-676.

22. Fernald D, Hamer M, James K, Tutt B, West D (2015) Launching a Laboratory Testing Process Quality Improvement Toolkit: From the Shared Networks of Colorado Ambulatory Practices and Partners (SNOCAP). J Am Board Fam Med 28: 576-583.

23. Fine PG, Bradshaw DH, Cohen MJ, Connor SR, Donaldson G, et al. (2014) Evaluation of the Performance Improvement CME Paradigm for Pain Management in the Long-Term Care Setting. Pain Medicine 15: 403-409.

24. Fisher S (2013) The development of a falls prevention and management toolkit for hospices. Int J Palliat Nurs 19: 244-249.

25. Fowles JR, Shields C, Barron B, McQuaid S, Dunbar P (2014) Implementation of Resources to Support Patient Physical Activity Through Diabetes Centres in Atlantic Canada: The Effectiveness of Toolkit-Based Physical Activity Counselling. Can J Diabetes.

26. Gibson SJ (2016) Translation of clinical practice guidelines for childhood obesity prevention in primary care mobilizes a rural Midwest community. J Am Assoc Nurse Pract 28: 130-137.

27. Gray E, Shields C, Fowles JR (2017) Building Competency and Capacity for Promotion of Effective Physical Activity in Diabetes Care in Canada. Can J Diabetes 41: 491-498.

28. Guillory C, Gong A, Livingston J, Creel L, Ocampo E, et al. (2017) Texas Pulse Oximetry Project: A Multicenter Educational and Quality Improvement Project for Implementation of Critical Congenital Heart Disease Screening Using Pulse Oximetry. Am J Perinatol 34: 856-860.

29. Gulati A, Harwood CA, Rolph J, Pottinger E, McGregor JM, et al. (2015) Is an online skin cancer toolkit an effective way to educate primary care physicians about skin cancer diagnosis and referral? J Eur Acad Dermatol Venereol 29: 2152-2159.

30. Haley WE, Beckrich AL, Sayre J, McNeil R, Fumo P, et al. (2015) Improving care coordination between nephrology and primary care: a quality improvement initiative using the renal physicians association toolkit. Am J Kidney Dis 65: 67-79.

31. Han C, Voils C, Williams J (2013) Uptake of Web-Based Clinical Resources from the MacArthur Initiative on Depression and Primary Care. Community Mental Health Journal 49: 166-171.

32. Henry JA, Orgoi S, Govind S, Price RR, Lundeg G, et al. (2012) Strengthening surgical services at the soum (first-referral) hospital: the WHO emergency and essential surgical care (EESC) program in Mongolia. World J Surg 36: 2359-2370.

33. Kinsinger LS, Jones KR, Kahwati L, Harvey R, Burdick M, et al. (2009) Design and Dissemination of the MOVE! Weight-Management Program for Veterans. Preventing Chronic Disease 6.

34. Koelling T (2006) Study: fewer heart deaths when QI efforts are made. Healthcare Benchmarks Qual Improv 13: 21-22.

35. Kuhlmann ZC, Ahlers-Schmidt CR, Kuhlmann S, Schunn C, Rosell J (2014) To improve safe sleep, more emphasis should be placed on removing inappropriate items from cribs. Obstet Gynecol 123 Suppl 1: 115S.

36. Lannon CM, Flower K, Duncan P, Moore KS, Stuart J, et al. (2008) The Bright Futures Training Intervention Project: implementing systems to support preventive and developmental services in practice. Pediatrics 122: e163-171.

37. Latsko J, Dennison B, Houk A, Chisolm S, Gerds A, et al. (2015) USE OF THE APLASTIC ANEMIA AND MDS INTERNATIONAL FOUNDATION'S TREATING MDS TOOLKIT CAN INCREASE THE FREQUENCY OF MDS EDUCATION AND SIDE EFFECTS BY ONCOLOGY NURSES. Oncology Nursing Forum 42: E216-E216.

38. Leape LL, Rogers G, Hanna D, Griswold P, Federico F, et al. (2006) Developing and implementing new safe practices: voluntary adoption through statewide collaboratives. Qual Saf Health Care 15: 289-295.

39. Levy S, Ziemnik RE, Harris SK, Rabinow L, Breen L, et al. (2017) Screening Adolescents for Alcohol Use: Tracking Practice Trends of Massachusetts Pediatricians. J Addict Med.

40. Lyndon A, Cape V (2016) Maternal Hemorrhage Quality Improvement Collaborative Lessons. MCN Am J Matern Child Nurs 41: 363-371.

41. Mabachi NM, Cifuentes M, Barnard J, Brega AG, Albright K, et al. (2016) Demonstration of the Health Literacy Universal Precautions Toolkit: Lessons for Quality Improvement. J Ambul Care Manage 39: 199-208.

42. MacDonald-Wilson KL, Hutchison SL, Karpov I, Wittman P, Deegan PE (2017) A Successful Implementation Strategy to Support Adoption of Decision Making in Mental Health Services. Community Ment Health J 53: 251-256.

43. Main EK, Cape V, Abreo A, Vasher J, Woods A, et al. (2017) Reduction of severe maternal morbidity from hemorrhage using a state perinatal quality collaborative. Am J Obstet Gynecol 216: 298.e291-298.e211.

44. McHugo GJ, Drake RE, Whitley R, Bond GR, Campbell K, et al. (2007) Fidelity outcomes in the national implementing evidence-based practices project. Psychiatric Services 58: 1279-1284.

45. Miller AE, Cohen BA, Krieger SC, Markowitz CE, Mattson DH, et al. (2014) Constructing an adaptive care model for the management of disease-related symptoms throughout the course of multiple sclerosis-performance improvement CME. Multiple Sclerosis Journal 20: 18-23.

46. Mitchell SE, Martin J, Holmes S, van Deusen Lukas C, Cancino R, et al. (2016) How Hospitals Reengineer Their Discharge Processes to Reduce Readmissions. J Healthc Qual 38: 116-126.

47. Mueller SK, Kripalani S, Stein J, Kaboli P, Wetterneck TB, et al. (2013) A toolkit to disseminate best practices in inpatient medication reconciliation: multi-center medication reconciliation quality improvement study (MARQUIS). Jt Comm J Qual Patient Saf 39: 371-382.

48. Mulloy DF (2008) Evaluation of implementation of the AORN Correct Site Surgery Tool Kit and the universal protocol for wrong site surgery: University of Massachusetts Boston. 166 p p.

49. Nace DA, Perera S, Handler SM, Muder R, Hoffman EL (2011) Increasing influenza and pneumococcal immunization rates in a nursing home network. J Am Med Dir Assoc 12: 678-684.

50. Lees M, Logan B, Theodoropoulos GK (2006) Agents, games and HLA. Simulation Modelling Practice and Theory 14: 752-767.

51. Nowalk MP, Nolan BA, Nutini J, Ahmed F, Albert SM, et al. (2014) Success of the 4 Pillars Toolkit for Influenza and Pneumococcal Vaccination in Adults. J Healthc Qual 36: 5-15.

52. Parkman CA (2013) Evaluation of an educational intervention on perceptions of a patient safety culture among staff in acute care nursing units: University of Nevada, Las Vegas. 154 p p.

53. Perumalswami PV, Vu T, Wyatt B, Parrella K, Rogers J, et al. (2016) Implementing HepCure - An Innovative Web- based Toolkit for Hepatitis C to Train Primary Care Providers and Increase Patient Engagement. Hepatology 64: 379A-379A.

54. Pierce C, McGinn K, Mulherin DW, Gonzales J (2016) A MULTICENTER STUDY COMPARING PROTOCOL IMPLEMENTATION WITH AND WITHOUT THE SCCM PROTOCOL TOOLKIT. Critical Care Medicine 44: 1.

55. Pratt S, Kenney L, Scott SD, Wu AW (2012) How to develop a second victim support program: a toolkit for health care organizations. Jt Comm J Qual Patient Saf 38: 235-240, 193.

56. Pulver LK, Oliver K, Tett SE (2012) Innovation in hospital quality improvement activities--acute postoperative pain management (APOP) self-help toolkit audits as an example. J Healthc Qual 34: 45-59.

57. Randle J, Clarke M, Storr J (2006) Hand hygiene compliance in healthcare workers. J Hosp Infect 64: 205-209.

58. Rueda-Clausen CF, Benterud E, Bond T, Olszowka R, Vallis MT, et al. (2014) Effect of implementing the 5As of obesity management framework on provider-patient interactions in primary care. Clin Obes 4: 39-44.

59. Ryan D, Barnett R, Cott C, Dalziel W, Gutmanis I, et al. (2013) Geriatrics, Interprofessional Practice, and Interorganizational Collaboration: A Knowledge-to-Practice Intervention for Primary Care Teams. Journal of Continuing Education in the Health Professions 33: 180-189.

60. Sample DA, Carroll HL, Barksdale DJ, Jessup A (2013) The Pediatric Obesity Initiative: Development, implementation, and evaluation. Journal of the American Academy of Nurse Practitioners 25: 481-487.

61. Sarna L, Bialous SA, Wells M, Brook J (2017) Impact of a webcast on nurses' delivery of tobacco dependence treatment. J Clin Nurs.

62. Schauberger CW, Larson P (2006) Implementing patient safety practices in small ambulatory care settings. Jt Comm J Qual Patient Saf 32: 419-425.

63. Septimus E, Hickok J, Moody J, Kleinman K, Avery TR, et al. (2016) Closing the Translation Gap: Toolkit-based Implementation of Universal Decolonization in Adult Intensive Care Units Reduces Central Line-associated Bloodstream Infections in 95 Community Hospitals. Clin Infect Dis 63: 172-177.

64. Shellhaas C, Conrey E, Crane D, Lorenz A, Wapner A, et al. (2016) The Ohio Gestational Diabetes Postpartum Care Learning Collaborative: Development of a Quality Improvement Initiative to Improve Systems of Care for Women. Matern Child Health J.

65. Shershneva MB, Harper PL, Elsinger LM, Olson CA (2010) Facilitating multiorganizational smoking cessation knowledge translation through on-line toolkit for educators and clinicians. J Contin Educ Health Prof 30: 149-150.

66. Smith PD, O'Halloran P, Hahn DL, Grasmick M, Radant L (2010) Screening for obesity: clinical tools in evolution, a WREN study. WMJ 109: 274-278.

67. Sopcak N, Aguilar C, O'Brien MA, Nykiforuk C, Aubrey-Bassler K, et al. (2016) Implementation of the BETTER 2 program: a qualitative study exploring barriers and facilitators of a novel way to improve chronic disease prevention and screening in primary care. Implement Sci 11: 158.

68. Spruce LR, Sanford JT (2012) An intervention to change the approach to colorectal cancer screening in primary care. J Am Acad Nurse Pract 24: 167-174.

69. Stalhandske E, Mills P, Quigley P, Neily J, Bagian JP (2008) Advances in Patient Safety

VHA's National Falls Collaborative and Prevention Programs. In: Henriksen K, Battles JB, Keyes MA, Grady ML, editors. Advances in Patient Safety: New Directions and Alternative Approaches (Vol 2: Culture and Redesign). Rockville (MD): Agency for Healthcare Research and Quality (US).

70. Stiff L, Vogel L, Remington PL (2014) Evaluating the implementation of a primary care weight management toolkit. WMJ 113: 28-31.

71. Taylor YJ, Tapp H, Shade LE, Liu TL, Mowrer JL, et al. (2017) Impact of Shared Decision Making on Asthma Quality of Life and Asthma Control among Children. J Asthma: 0.

72. Thomason SS, Powell-Cope G, Peterson MJ, Guihan M, Wallen ES, et al. (2016) A Multisite Quality Improvement Project to Standardize the Assessment of Pressure Ulcer Healing in Veterans with Spinal Cord Injuries/Disorders. Adv Skin Wound Care 29: 269-276.

73. Kohler C, Beck D, Villarreal CL, Trial JL (2015) Interprofessional Participation in a Statewide Collaborative to Recognize and Treat Hypertension in Pregnancy...Proceedings of the 2015 AWHONN Convention. JOGNN: Journal of Obstetric, Gynecologic & Neonatal Nursing 44: S50-S50.

74. Wyte-Lake T, Claver M, Der-Martirosian C, Davis D, Dobalian A (2017) Developing a Home-Based Primary Care Disaster Preparedness Toolkit. Disaster Med Public Health Prep 11: 56-63.

75. Zimmerman RK, Nowalk MP, Lin CJ, Hannibal K, Moehling KK, et al. (2014) Cluster randomized trial of a toolkit and early vaccine delivery to improve childhood influenza vaccination rates in primary care. Vaccine 32: 3656-3663.
